# Random Survival Forest Versus Elastic-Net Regularized Cox Regression for Survival Prediction in Acute Myeloid Leukemia at Distinct Treatment Time Points: Model Performance Comparison Study

**DOI:** 10.2196/75678

**Published:** 2026-04-29

**Authors:** Oisín Brady, Sean Johnson, Peter Giles, Caroline Alvares, Joanna Zabkiewicz, Carolina Fuentes

**Affiliations:** 1 School of Computer Science and Informatics, Cardiff University, Abacws, Senghennydd Road, Cardiff, CF24 4AG, United Kingdom, 44 (0)29 2087 4812; 2 School of Medicine, Cardiff University, Cardiff, United Kingdom

**Keywords:** acute myeloid leukaemia, AML17, time-to-event, survival prediction, digital twin, random survival forest, cox proportional hazard regression, elastic net

## Abstract

**Background:**

Risk group stratification based on the prediction of survival of patients with acute myeloid leukemia (AML) is complex. Despite common risk group categorization guidelines, the overall prognosis remains poor. Machine learning techniques have been shown to provide more accurate risk group stratification than conventional approaches using trial data. However, many time-to-event (TTE) models do not use training sets constrained to specific time windows, instead using aggregations of trial data.

**Objective:**

This study aimed to evaluate the performance of (1) random survival forest (RSF) and (2) Cox proportional hazard regression with elastic net regularization (CoxNet) for survival prediction of patients with AML within a censoring window trained with available data recorded at discrete time points during the United Kingdom National Cancer Research Institute Acute Myeloid Leukaemia 17 randomized controlled trial (AML17).

**Methods:**

For each stage in the AML17 trial, separate models were trained for each exhaustive k-choice combination of available AML17 data subsets. Data combinations for each model were further constrained according to the respective trial stage to avoid data leakage. Preliminary Pearson correlation methods were used to remove directly correlating features with the TTE prediction (time-to-death/5-y censoring point). Repeated k-fold stratified cross-validation was used on each dataset ablation to find candidate models. Permutation importance and elastic net regularization were used to monitor stability across validation folds and reduce the feature set of the highest performing stage RSF and Cox proportional hazard regression models, respectively. Finally, selected ablated models were re-evaluated using the nested, k-fold, stratified sampling cross-validation method with bootstrapping.

**Results:**

Concordance index ranked the best models for data constricted up to the end of induction (RSF=0.68, CoxNet=0.67), stages 1 (RSF=0.69, CoxNet=0.68), 2 (RSF=0.68, CoxNet=0.66), and 3 (RSF=0.69, CoxNet=0.63) of the trial.

**Conclusions:**

This study details the high prediction accuracy for time-to-survival-event predictions when training sets of CoxNet and RSF models, which are sequentially constricted to data measured up to the end of respective AML17 trial stages. The performance of these sequential TTE models is intended to justify their use as part of a wider digital twin system simulating multiple TTE outcomes for patients with AML.

## Introduction

Cancer is an enigmatic issue with its pervasiveness seemingly as wide as the depth of its biological origins. As a group of diseases, cancer is highly variable in form, with roughly 200 types according to the National Cancer Institute [1]. One such type of cancer, acute myeloid leukemia (AML), occurs from genetic abnormalities in precursory cells responsible for differentiating into mature platelets, white blood cells, and red blood cells. Genetically, AML is highly stratified, with multiple potential mutation points in the lineage of precursory cells responsible for mature blood cell types, such as within the earliest hematopoietic progenitor cell stage, or by mutation of immature intermediate blast cells [2]. Ultimately, AML causes uncontrolled proliferation of immature or nonfunctional blood cells, leading to systemic immune dysfunction and organ complications. AML is often subcategorized into primary (or de novo) AML and secondary AML; secondary AML is further subdivided into therapy-related AML or acute myeloid leukemia derived from antecedent hematological disorders, such as myelodysplastic syndrome [3]. Multiple genetic and environmental factors contribute to the cause and progression of AML, such as trisomy 21 (Down syndrome) [4], Fanconi anemia [5], Nucleophosmin 1 (NPM1) [6], or FMS-like (Feline McDonough Sarcoma [7]) tyrosine kinase 3 (FLT3) [8] mutations. External factors associated with AML include prolonged exposure to benzene [9], history of smoking [10], or cancer therapy–induced AML [11]. This heterogeneous nature of AML attributes to its difficulty to precisely diagnose and treat effectively [12].

Deriving accurate survival predictions of patients with AML is an important foundational step in establishing risk groups upon which accurate treatment methods can be produced. Resultingly, there is a great emphasis on risk group stratification of patients with AML as a precursory step for optimized resource allocations and identification of biomarkers contributing to treatment response, as seen in the European LeukemiaNet (ELN) project [13] or the World Health Organization (WHO) risk grading of AML [14]. Within recent years, the availability of genomic data through next-generation sequencing (NGS) [15] techniques combined with randomized controlled trial datasets, such as “the United Kingdom National Cancer Research Institute Acute Myeloid Leukaemia 17 (AML17) trial” [16-18], offers a wealth of information to make inferences on the disease and improved treatments. Indeed, 5-year overall survival rates for AML have improved, from 13% in 1970 to 55% (*P*<.001) in 2010 for patients younger than the age of 60 years at the MD Anderson Cancer Center hospital and from 8% in 1970 to 17% for those older than 60 years [19]. The Surveillance, Epidemiology, and End Results program based in the United States estimates the 5-year overall survival rate of patients with AML to be 31.9% based on survival data between 2014 and 2020, and estimates 20,800 new cases of the disease, constituting approximately 1% of new cancer cases in the United States in 2024, with 11,220 AML-related deaths [20].

Despite improvements in outcome, it is apparent that traditional hierarchical approaches for risk grouping are not able to capture the full complexity involved in stratification of AML [21], shown by the still dismal net survival rate of 13.6%, 5 years after diagnosis in England [22]. With the sheer quantity of data now available from NGS and randomized controlled trial databases, alternative machine learning (ML) techniques used within oncology [23] have been shown to capture complex features stratifying patients with AML [24,25]. The United Kingdom National Cancer Research Institute (UK-NCRI) AML17 trial contains detailed records of clinical, longitudinal minimal residual disease (MRD) reports and genetic sequence mutation profiles of approximately 3142 patients with AML younger than 60 years between 2007 and 2014. Such a large, time-based dataset offers an ideal training set for ML models to capture complex risk stratification of the disease. The original AML17 protocol used standard statistical methods, such as the log-rank test for time-to-event (TTE) outcomes (survival for all randomizations), Mantel-Haenszel tests for dichotomous outcomes, and Wilcoxon rank-sum and *t* tests for resource usage data. It has more recently been shown that data from AML17 can be used for highly accurate ML risk group stratification based on survival prediction. Tazi et al [26] applied several ML models trained on demographic, diagnostic, and genetic variables from several UK-NCRI AML trials, including AML17. By fitting models to predict overall survival via TTE of patient death up to censoring points, patients could be stratified by predicted survival risk measurements and separated into distinct groups based on delineating features. When compared with the ELN guideline, this new framework restratified 1 in 4 patients, with significantly improved prognostic accuracy. Another study using the following AML18 trial [27] used a random survival forest (RSF) model to update risk group stratification categories based on overall survival using age, sex, white blood cell count, gene mutations, and cytogenetic abnormalities of patients. Subsequently, numerous patients were restratified from risk groups in the standard 2022 ELN guideline, which could be used to retrospectively identify more optimal treatment paths [28].

Several ML models are specifically designed or adapted for TTE outcome prediction using right-censored data [29,30] as seen in AML trial datasets.

One such ML model, an adaptation of the random forest algorithm [31], RSF [32], as previously mentioned, has been used for time-dependent survival predictions, which excludes the proportional hazards assumption of statistical Cox proportional hazard regression (CPHR) models. CPHR is a statistical model commonly used across multiple scientific domains, including cancer research [33] for TTE predictions. In the case of AML, where multiple interacting and time-dependent biomarkers affect survival outcome [12], collinear features can negatively impact prediction accuracy when the independent features and proportional hazards assumptions of CPHR models are violated [34]. In such cases, the standard CPHR model can be adapted using regularization techniques such as the “Elastic Net” method (also known as “CoxNet” [Cox proportional hazard regression with elastic net regularization]) [35].

Performance between the 2 models varies depending on the datasets and implementation. Several instances in literature show that RSF prediction is comparable with or even outperforms CPHR models [36-38]. However, the converse is also referenced [39-41], suggesting that the application of these models is highly dependent on initial training datasets, preprocessing, model building methodologies, and the overall complexity of the predicted outcome. Pickett et al [42] conclude that RSF performs best when leveraging its nonlinear nature with multiple, longitudinal data points, many of which have unknown levels of significance.

None of the ML-based studies reviewed involved static and longitudinal training sets that were constricted according to trial time frames and sequentially exposed to more data, instead using an aggregation of data. This study seeks to investigate the predictive performance of individual TTE models, beginning with 5-year survival status, which are sequentially exposed only to data available up to the conclusion of major time points in the AML17 trial. RSF and CPHR with Elastic Net have been chosen as TTE predictive models given their previous application in this context. A pipeline involving necessary data preprocessing, feature selection, and hyperparameter tuning used to build each will be detailed. Finally, after evaluation using the primary concordance index (c-index) metric alongside additional dynamic area under the receiver operating characteristic curve (also known as dynamic AUC) and Brier loss scores, the optimal models for survival prediction at select trial stages will be selected for future analysis. Future studies will analyze select model feature importance and significance with respect to state-of-the-art literature on AML risk stratification. The generalized pipeline will also serve as a template that can be adapted for additional TTE predictions other than death status. In the wider context of a “digital twin” [43] system, this multiple time-constrained model approach could provide accurate simulations of a wide variety of patient outcomes, not solely focused on risk stratification but also patient quality of life (QoL) and optimized care for additional comorbidities during treatment.

## Methods

### AML17 Trial Data

Data are sourced from the AML17 [17] drug trial for patients younger than 60 years, which includes 3142 clinical records. Patient clinical records are combined with MRD (n=2587), NGS mutation profiles (n=3579), and a separate collection of *NPM*1 [6] and *FLT*3 specific mutation profiles (n=3142) [44]. A pseudonymization process converted patient trial IDs into dummy IDs before data access, ensuring compliance with participant privacy and data protection regulations. Access to the pseudonymized dataset was stored on the Cardiff University Research Data Store [45] with access restricted to authorized researchers. Clinical records contain measures at induction, including previous blood disorders, height, weight, the French-American-British 8-category AML classification [46], WHO and Eastern Cooperative Oncology Group performance status [47], cytogenetic, karyotype, ethnic background, and more. Metadata references to all used data are available in Multimedia Appendices 1-4.

After early diagnostic and comorbidity measurements during the induction stage, the proceeding 4 stages contain longitudinal records on periodic treatment, response, toxicity, and supportive care. The MRD subset includes quantitative polymerase chain reaction on peripheral blood and bone marrow samples, and multiparameter flow cytometry using the leukemia-associated immunophenotype and “different from normal” techniques. MRD measurements are longitudinal; the trial protocol involved readings at the end of each major trial stage investigated in this study. AML17 collected FLT3 and NPM1 mutation profiles with automated flow cytometry and manual bone marrow and peripheral blood cytology measurements at days 2‐3 from patient induction to the trial.

### Ethical Considerations

Access to data from the UK-NCRI AML17 clinical trial was provided by the Cardiff University Centre for Trials Research [48], which curates and governs the trial database.

All data were pseudonymized before being released to the research team. No direct patient identifiers were included in the dataset. All analyses were conducted on secure Cardiff University computing infrastructure. Ethical approval for the use of these data was granted by the Cardiff University School of Computer Science and Informatics Research Ethics Committee on February 28, 2025 (approval COMS/Ethics/2024/014). The research used secondary analysis of previously collected clinical trial data and did not involve direct contact with participants. Participants in the original AML17 trial provided written informed consent for their data to be used for research purposes. No compensation was provided to participants for this study, as it involved secondary analysis of previously collected trial data.

### Data Cleaning

#### Overview

Paper Case Report Forms recorded data throughout the AML17 trial. An initial screening process of longitudinal data found value errors in the exported dataset, predominantly within date fields. The following sections detail the conditions for sample exclusions based on erroneous record entries.

#### Erroneous Record Removals

Most detectable erroneous values are date records, leading to the exclusion of 85 patient records with date values written outside of the trial time bounds between 2007 and 2014 (excluding annual follow-up dates that proceed after the official trial end date, ie, July 31, 2014) or with nonsequential or otherwise nonchronological trial stage entry times, likely due to data entry errors or outstanding queries with sites at trial closure. The exclusion of nonsequential course start dates removed 19 patient records from the study. After all initial exclusions, 3057 AML patients remained eligible for model training.

#### Feature Removals Before Feature Selection

The pseudonymized dummy ID was dropped before model training to avoid spurious correlation from potential protocol batch induction bias. Other exclusions include nonstandardized clinician notes, making them highly varied text fields, which are not immediately processable by RSF and CPHR models. Traditional preprocessing methodologies, such as dummy encoding, would introduce many Boolean representations of these features, most of which, given their variability, would have occurrences recorded seldomly, increasing data sparsity and potentially increasing model risk of overfitting [49]. Consequently, categorical or continuous features from these columns cannot be immediately cataloged. Information held in these fields is of potential clinical and ML model importance. However, additional preprocessing techniques are needed to scrape the potentially multiple continuous and categorical features existing in a single record. Data capture would also need to handle the detection of differently written versions of the same category (eg, syntactic, spelling, or grammatical variations). Data mining using ML techniques, such as the usage of natural language processors [50], may be explored for text classification of these fields in future studies. A breakdown of such excluded features is available in Multimedia Appendix 5.

### Preprocessing

#### Overview

Clinical, NGS, FLT-3, and MRD status records initially existed as separate pseudonymized comma-separated values (csv) files exported from the original AML17 trial database within the Cardiff University Centre for Trials Research. Each file was merged using the pseudonymized “DummyID” of each patient to produce unique patient records of all exported csv data. The training set was initialized using the Python “Pandas” [51] library “DataFrame” object [51], storing merged patient records. The data type was specified for each column and programmatically converted individual records that violate the expectation, if possible; otherwise, the patient record was dropped. This ensured that each feature vector is readable to the applied CoxNet and RSF models. The following sections define the data preparation steps necessary for model training.

#### Feature Set Data Representations

The AML17 dataset contains 2 general data types—continuous and categorical. Continuous features were scaled using Sci-Kit Learn’s “StandardScaler” function [52], which computes a standard score of each sample based on its variance from the mean of the feature vector. The standardized continuous Unix Epoch time is used to represent all instances of date fields. Unix Epoch time denotes the total nonleap seconds elapsed since 00:00:00 UTC on January 1, 1970 [53]. While scaling is not a requirement for tree-based ensemble models, such as RSF, whose results are insensitive to the transformation [54], applying it to continuous variables avoids scenarios where features that are orders of magnitude higher than others influence the objective function disproportionately within CPHR models. This also standardizes data representations for training sets of both models. Dummy encoding is used to convert categorical features into discrete Boolean features for each level, which is readable to the CPHR model.

Given that the AML17 dataset includes many categorical features, dummy encoding drastically increases the total number of features fed into both models, increasing the potential complexity of the model and potentially introducing overfitting. Feature reduction techniques are used to attenuate this. For RSF, the permutation importance [55] technique is used to quantify feature importance. This involves randomly shuffling feature values a set number of times and measuring the effect on model performance each time through the c-index evaluation. Degradation of performance when changing these values indicates the RSF’s relative reliance on a particular feature. The combination of 2 regularization methods, known as elastic net, was used to reduce the CPHR feature set. This combines ℓ1and ℓ2 regularization methods [35], simultaneously handling issues of collinearity within the dataset as well as feature reduction.

#### TTE Predictor Variable

CoxNet, RSF, and similar models used for time-based prediction, such as support vector machines for survival [56,57], use a target variable known as the “time-to-event” (TTE) variable, formalized by Equation 1 :


(1) $$y=\min(t,c)=\left\{ \begin{array}{ll} t & \text{if }\delta =1,\\ c & \text{if }\delta =0 \end{array}\right.$$

where δ is a Boolean value representing event occurrence (in this instance, patient death), *t* being the time range from patient trial induction to the event, and *c* being the time range from patient trial induction to the censoring threshold.

Equation 1 is modified to shift the censoring window of patient records to 5 years from induction, formalized as Equation 2:


(2) $$\begin{array}{c} y=\left\{ \begin{array}{ll} t & \text{if}\delta =1\wedge t<c,\\ c & \text{otherwise} \end{array}\right. \end{array}$$

This accounts for instances of patients who have died on either side of a 5-year censoring window, a threshold seen in follow-up analysis of AML17 [58]. A 5-year cutoff point provided a more even distribution of noncensored patients than lower thresholds for TTE prediction models. Clinical variables define the TTE variable as a tuple: (δ,(t|c)), with inclusion of t or c depending on the censoring status of the event as described in equation 2. Both the ablation and final model tuning methods stratify each fold to ensure that the distributions of TTE indicators are approximately equally distributed with respect to the entire cohort to avoid nonrepresentative sampling issues.

#### Trial-Stage Sensitive Ablation Study

To determine what broad groups of initial training datasets were most important for each model, we conducted an ablation study that selected possible combinations of NGS, FLT3 and NPM1, clinical, and MRD data subsets. C-index evaluations of tuned and validated models ranked the combination relative importance. Permutation importance [55] and nonzero coefficient values determined individual feature importance for RSF and CoxNet models, respectively. Multiple RSF and CoxNet models predicted the TTE target after being trained on subsets of patients surviving up to the end of each AML17 course stage, that is, induction, C1, C2, and C3. We trained models at each stage on varying degrees of information based on precomputed data subset combinations. The data pulled from clinical and MRD subsets were constrained such that each model only had access to features recorded up to the end of their respective course stage to avoid potential data leakage and provide predictions using data measurements only available up to specific trial time points. We define the set of training data combinations as all possible K-choice, non–order-specific, nonrepeating items at each trial stage, formalized as:


(3) $$\sum_{c=1}^{5}\sum_{k=1}^{3}\frac{3!}{\left(3-k\right)!k!}=35$$

where c represents the current trial stage (induction, C1, …, and C3), and k the number of selected data sources.

The protocol recorded FLT3, NPM1, and a broader collection of NGS mutation panel measurements for patients 2‐3 days after their randomized induction to the trial; therefore, not necessitating further constriction, as all-time points are set after recording of these data.

Direct Pearson linear correlations to death status determined the exclusion of features from all data combinations. Likewise, we excluded categorical features with options that inferred patient death status. Excluded features are detailed in Multimedia Appendices 1-3. In total, with 4 time point stages (induction, C1, C2, and C3), the total k-choice combinations across each stage equaled 35 training sets. These trained a combined total of 70 CoxNet and RSF models.

### Model Building

#### Overview

The model building process is divided into four major phases, that are (1) preprocessing; (2) ablation study; (3) final evaluation, based on the highest performing candidate ablations for RSF and CoxNet models at each trial stage; and (4) a baseline risk model comparison, which compares RSF and CoxNet c-indices against a standard Cox model used within the trial protocol to stratify patients post stage 1.

#### Preprocessing Phase

The following defines the overall steps involved in the preprocessing pipeline on data that are made consistent between both of the following phases. Cleaning methods detailed in earlier sections have been excluded for simplicity (refer to Data Cleaning section). The preprocessing pipeline is called and fit to data only available within the scope of the fold used within cross-validation (CV) of the ablation study and final model evaluation phases.

1. Drop all features with total missing entries >95%.
2. Create missing indicator features for each feature with at least 1 occurrence of a missing value.
3. Flatten the NGS data subset of gene mutation entries, dummy encode, and merge with the rest of the combined dataset (clinical, MRD, FLT3, and NPM1 subsets). Encoding is based only on available samples within the fold to avoid potential leakage of nonfold sample gene mutation entries.
4. Dummy encode karyotype features, labeling rare entries (n<5) to a “rare_class” category to avoid dimensionality explosions.
5. Dummy encode all other standard categorical features.
6. Preserve the ordinality of the identified ordinal record by keeping them as individual features, using predefined integer mappings from the protocol. Missing features are labeled with the sentinel value −999 consistently outside of all ordinal ranges.
7. Scale identified numerical features by removing the mean and scaling to unit variance (using Sci-Kit Learn’s StandardScaler)

#### Ablation Study Phase

The goal of this phase is to act as a sensitivity analysis of the major data sources available from the AML17 trial. By using every possible combination of these data sources (refer to equation 3), the most influential data can be determined using the average c-index performance. This, in turn, acts as a feature reduction step and ensures selected ablations for downstream analysis are using features of importance relative to the specific model and respective trial stage.

The following steps define the preliminary ablation study phase. For each ablation at each trial stage, the cohort applies repeated (n=3), stratified, 5-fold CV using a consistent random state seed for reproduction.

1. Feature preprocessing phase pipeline fit to the training set and transformed on the train and validation set within the fold scope.
2. Train a baseline RSF model on the fold’s training set.
3. Apply permutation importance on the baseline model (using 150 ensemble estimators and a consistent randomized state seed), recording feature stability per repeated fold.
4. Measure the c-index and inverse probability of censoring weights (IPCW) c-index of the baseline ablation RSF model.
5. For the same fold repetition, train a baseline CoxNet model and record feature stability using model coefficient values.

#### Final Evaluation Phase

The following steps define the final model-building and evaluation phase:

1. At each stage, select a CoxNet and RSF ablation model with the highest recorded average c-index across all repeated CV folds from the ablation study.
2. For each selected model, using their respective ablated dataset and trial cohort, use nested k-fold, stratified CV, with sample shuffling and the same randomization seed used consistently across all experiments.
  - The outer loop is reserved for unbiased performance estimation across 10 folds of the training set.
  - The inner loop is reserved for hyperparameter tuning across 3 folds. Validation samples are never included in model training in their respective loop, ensuring strict separation between training and validation data to avoid bias or overfitting. Grid search spaces for hyperparameter tuning of the models are:
    - RSF: ‘n_estimators’ = [500, 750, 1000, 1250, 1500]
    - RSF: ‘max_features’ = [‘sqrt,’ ‘log2,’ 0.33, 0.5]
    - RSF: ‘max_features’ = [3, 5, 10, 15]
    - CoxNet: ‘l1_ratio’ = [0.01, 0.25, 0.5, 0.75, 0.8, 0.85, 0.9, 0.95, 0.99, 1.0]
  - Record fold’s bootstrapped performance estimates across 250 bootstrapped samples (250 samples chosen as a compromise between sample variance for CI precision and processing time constraints with the already expensive nested CV operations). Metrics include c-index, IPCW c-index, dynamic AUC, and dynamic Brier loss.

As fold event times vary across bootstraps, dynamic AUC and Brier loss metrics for fold bootstrap samples are interpolated to the nearest predefined “universal” time points for consistency between selected samples across the validation process.

#### Baseline Risk Model Comparison Phase

For comparison with the Cox linear regression model used in the AML17 trial protocol for risk assessment used on the cohort ending their first stage of treatment, an additional *non-nested* grid search CV assessment on a 90%:10% train-test split with 1000 bootstrapped samples was conducted. We recorded dynamic AUC, Brier loss, IPCW c-index, and c-index scores of both models at this stage. The purpose of this analysis is to suggest improved c-index performance of the respective CoxNet and RSF stage models against the protocols model. The rationale for using larger sample sizes for this assessment was to provide a more realistic indication of the model’s performance relative to the protocol’s model when trained with a sample size closer to real-world cases. As the test set is smaller and overlaps with hyperparameter tuning, this assessment is inherently optimistic. Therefore, it remains that the nested CV results with bootstrapped CIs illustrated in the Final Evaluation Phase section remain as the primary, more sensitive, and pessimistic evaluation of generalized model performance.

### Model Evaluations

#### Overview

The c-index evaluated model performance from a held-out test set not used in previous training. The standard (Harrel) c-index used for survival model evaluation is dependent on the distribution of the censored events. Therefore, to avoid potential bias, we recorded an alternative adapted form of c-index based on the IPCW. However, likely due to the inner handling of censoring by the models, differences in IPCW c-index and standard c-index were none, or at most minuscule, so standard c-index remained the primary performance metric for model assessments. Both standard and IPCW adapted c-index results for all final models have been included for transparency. Secondary performance metrics involved:

1. Dynamic AUC [59] assessed predictive performances across patients selected at discrete time points from the end of the model’s respective trial stage to the 5-year censoring point.
2. A time-dependent Brier loss score, measuring mean square difference between predicted and real TTEs at iterative time points, indicated the models’ calibration.

#### Cumulative-Dynamic AUC

This performance metric assesses the model’s ability to discriminate between patients who experience an event before a specific time period (t), and those who experience an event after [59]. AUC ranges from 0 to 1 inclusively, with higher values indicating better discrimination between patient events before and after t.

#### Dynamic Brier Loss Score

This performance metric assesses how well a model is calibrated, evaluating how closely model predictions match the real labeled TTE variable of a patient at time point t, typically referred to as the “ground truth.” This is done by evaluating the difference in mean square predicted event times and the ground truth TTE at t. Brier loss at t  is measured between 0, for models with perfect accuracy, and 1, for perfect inaccuracy. The integrated Brier loss score can also be measured to evaluate overall model calibration throughout the 5-year period since patient induction.

### Pipeline Summary

**Figure 1.**
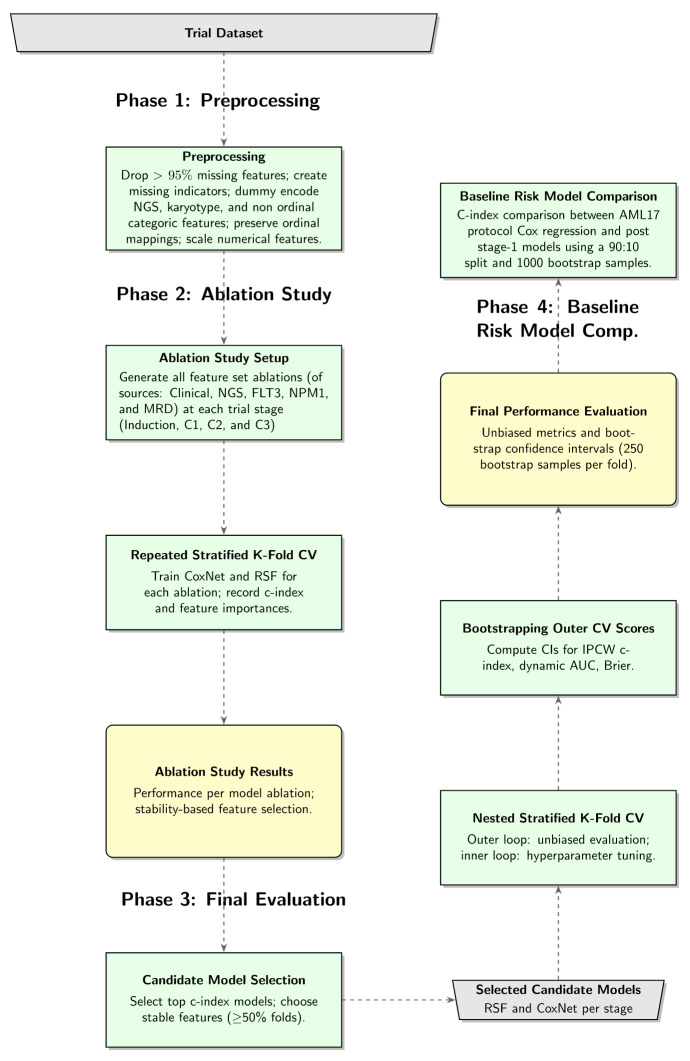
Summary of the 4-phase pipeline process for survival time-to-event prediction model building at the AML17 trial stages. AML17: United Kingdom National Cancer Research Institute Acute Myeloid Leukaemia 17 randomized controlled trial; AUC: area under the receiver operating characteristic curve; c-index: concordance index; CoxNet: Cox proportional hazard regression with elastic net regularization; CV: cross-validation; FLT3: Feline McDonough sarcoma-Like Tyrosine kinase 3; MRD: minimal residual disease; NGS: next-generation sequencing; NPM1: Nucleophosmin 1; RSF: random survival forest.

A diagrammatic summary of the model training pipeline, briefly describing the 4-phase process detailed before, is presented in Figure 1. Descriptions of CoxNet and RSF are detailed in Multimedia Appendix 6 and Multimedia Appendix 7. Code for relevant experiments is available in Multimedia Appendix 8.

## Results

### Overview

Results are composed of, first, event (patient death within censoring window) status and timing distributions are measured, describing the spread of target events across cohorts and time for RSF and CoxNet models. Second, a Kaplan-Meier survival curve is shown for each cohort, including the full AML17 cohort, after cleaning for erroneous TTE indicators described in the Data Cleaning section. These curves provide a visual summary of the baseline survival patterns between cohorts, which the stage-specific survival models are tasked with capturing. Third, feature missingness correlations are visualized using heatmaps for data sources across AML17 cohorts, highlighting potential missingness mechanisms and motivating the usage of missing indicator variables for models. Fourth, Table 1 reports feature set sizes before and after reduction steps for RSF and CoxNet models, showing how reduction methods remove redundant features, focusing on the most informative and stable predictors quantitatively selected during phase 3 of the methodology. Fifth, c-index, dynamic AUC, and dynamic Brier quantify ranking accuracy, time-dependent discrimination, and overall predictive accuracy for each stage, providing a suite of metrics for cross-reference with similar work and reproducibility. Finally, feature importance is visualized using Venn diagrams of overlapping top-ranked features between stage-specific RSF and CoxNet models, and vertical bar charts illustrate the relative importance of the top 30 highest-ranking predictors in each model.

**Table 1.** Feature set reductions for each RSF^a^ and CoxNet^b^ model at their corresponding trial stage.

Trial stage	Feature reduction (n before, n after, n after encoding)
Post induction	RSF: 78, 70, 392 CoxNet: 56, 45, 205
Post-C1	RSF: 156, 126, 479 CoxNet: 115, 85, 225
Post-C2	RSF: 228, 173, 596 CoxNet: 228, 168, 463
Post-C3	RSF: 290, 142, 478 CoxNet: 211, 174, 452

a RSF: random survival forest.

b CoxNet: Cox proportional hazard regression with elastic net regularization.

### Target Class Distributions

TTE (event=death status) of both models is the predictor variable of both models. Class imbalances influence the overall performances of a model [60]. There were no major class distribution imbalances for each of the 5 trial stages used for model training. No training sets had minority class percentages <41% and the average minority class percentage was approximately 44% (induction=42.8%, C1=46.9%, C2=48.9%, C3=41.3%, C4=42.9%). In literature, there is no definitive threshold at which imbalances are considered severe enough to affect the performance of ML models. A rule of thumb is that an imbalance is considered “moderate” when minority classes are 1%‐20% of the dataset [61]. Since this was not the case, the use of over- or undersampling techniques or synthetic data generation techniques such as the Synthetic Minority Oversampling Technique [62] was deemed not necessary.

### Event Time Distributions

**Figure 2.**
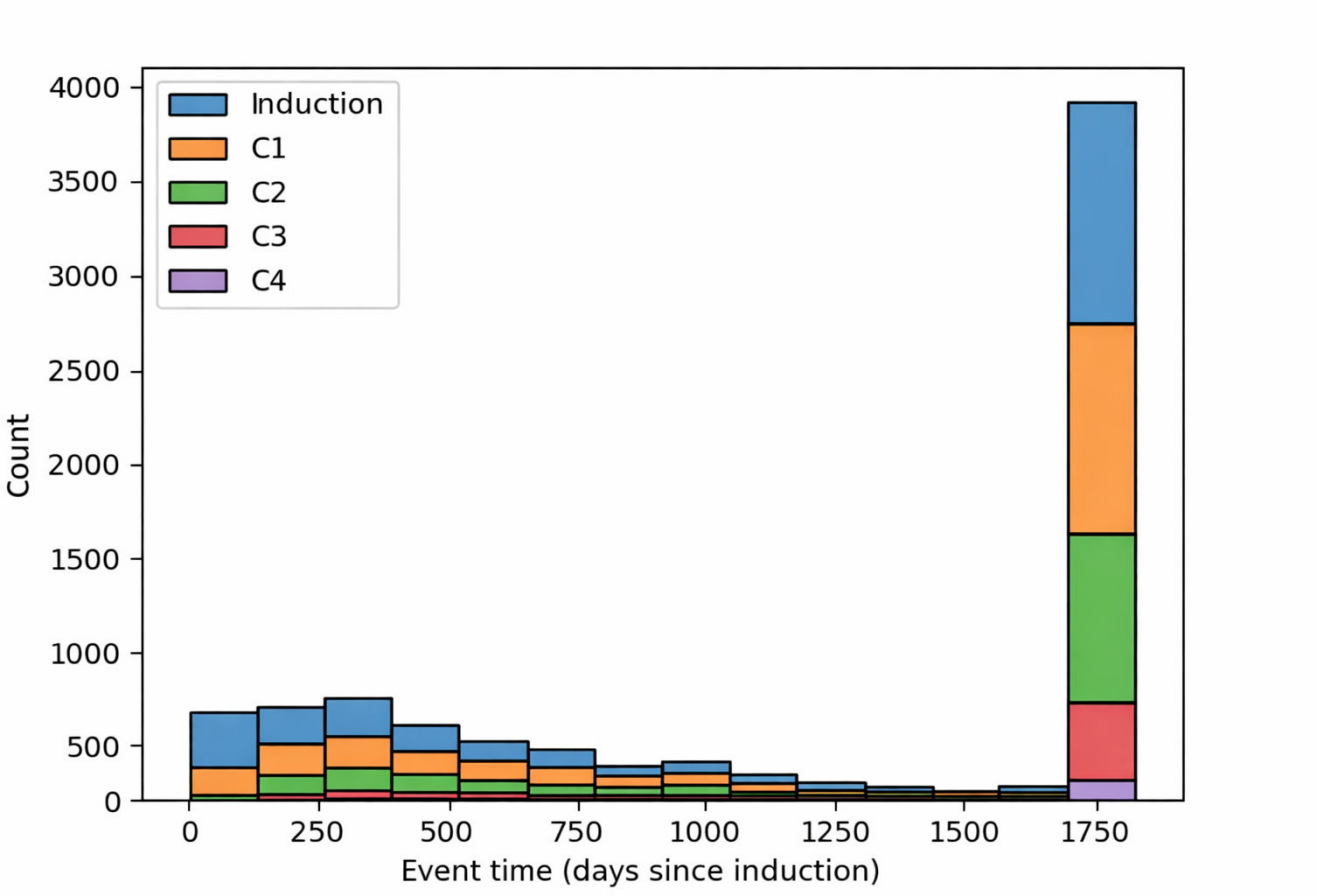
Distribution of time-to-event target variables in each of the four trial stages with censoring 5 years from patient induction. The distribution is irregular for all stages with the final histogram bin of induction, C1, C2, and C3 stages (C4 was excluded from further experiments because of small sample size concerns [n=177]).

Analysis of event time distributions in Figure 2 shows a disproportionate number of right-censored events for patient sets used for each of the 5 selected trial stages. This initially justified the usage of a c-index based on IPCW, which is specifically adapted for this situation [63] rather than the standard c-index, which is dependent on TTE distributions. However, it was found that differences between c-index and adapted IPCW c-index readings of CoxNet and RSF models were identical for stage models (eg, postinduction stage mean c-index=0.6760, mean IPCW c-index=0.6760). Full performance metric results across all stage ablation models are included in Multimedia Appendix 9.

### Cohort Kaplan-Meier Survival Curves

**Figure 3.**
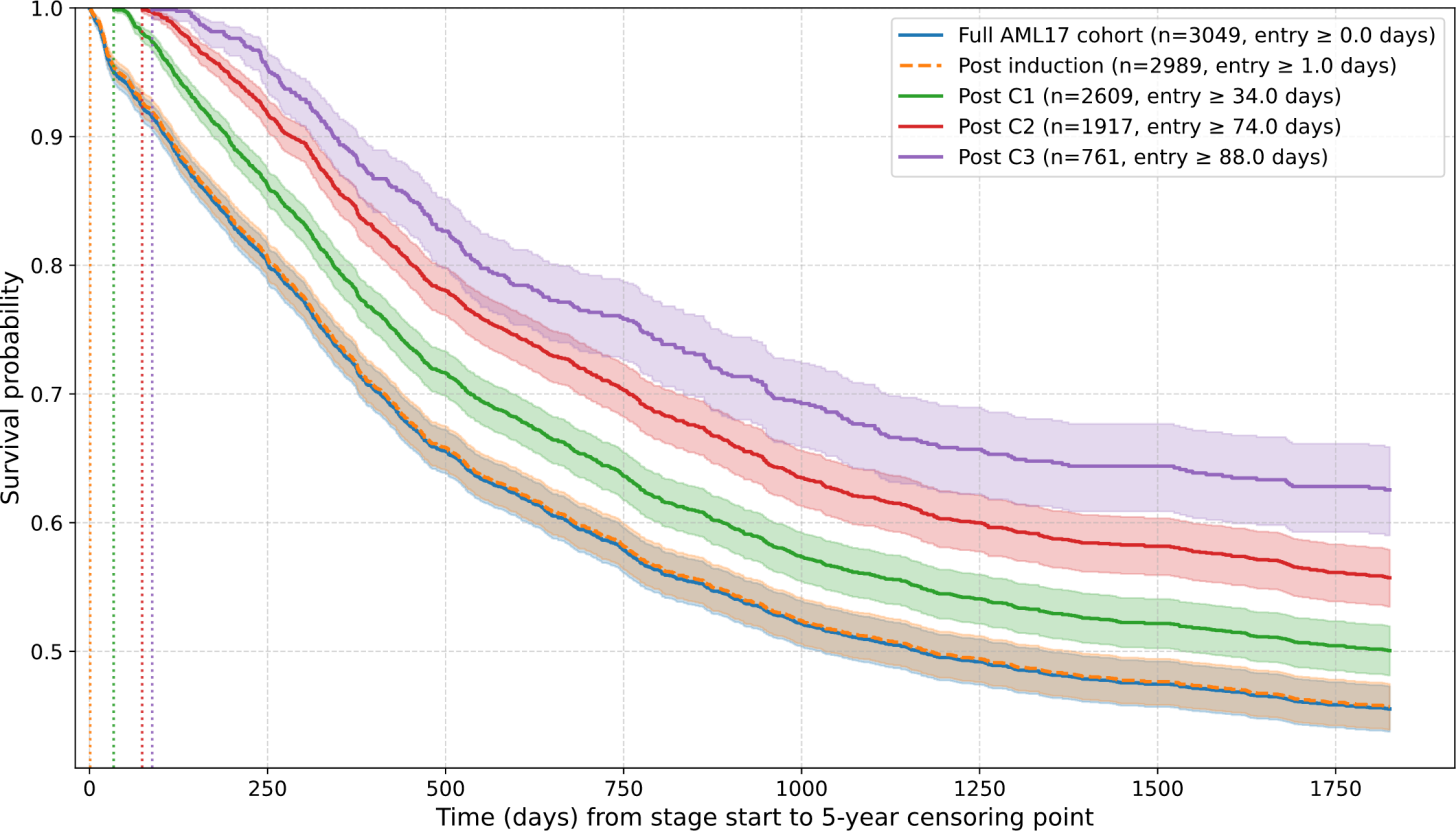
Kaplan-Meier survival curves for each cohort used by random survival forest and Cox proportional hazard regression with elastic net regularization models after data cleaning. The full AML17 cohort (post data cleaning) is also shown (blue). Shaded regions represent upper and lower CIs, solid lines (made dashed orange for postinduction for readability) represent average survival probability at the time point. Vertical dashed lines show trial stage cohort earliest start times since induction, with exact values shown in the legend.

Kaplan-Meier survival curves for each patient cohort for modeling show distinct survival patterns between trial stage cohorts, excluding the full cohort upon trial entry and the postinduction stage, as it began within 1‐3 days according to the AML17 protocol. This gives an indication of the distinguishable baseline survival patterns that vary between AML17 stage cohorts, which each model is tasked to predict. For visual clarity, the posttreatment stage 4 cohort was excluded from Figure 3, as it overlaps across many cohorts. Note that the posttreatment stage 4 was excluded from further modeling due to small sample size concerns (n=177).

### Data Missingness

Data missingness, nonexistent trial records, per trial stage cohort, and full AML17 cohort (post–data cleaning record exclusions) have been visualized using heatmaps for missingness correlation between features and frequency missingness plots for overall record missingness. Given the volume of total features, each figure has been further divided by data subset (eg, Clinical or NGS). The following matrix shows the missingness correlation between all clinical variables across the whole AML17 cohort used in this study. This provides justification for the usage of the missingness indicator features supplied as additional predictors for each model, which can be used to assess the informativeness of missingness and additional follow-up analysis. All additional figures have been included in Multimedia Appendix 10.

**Figure 4.**
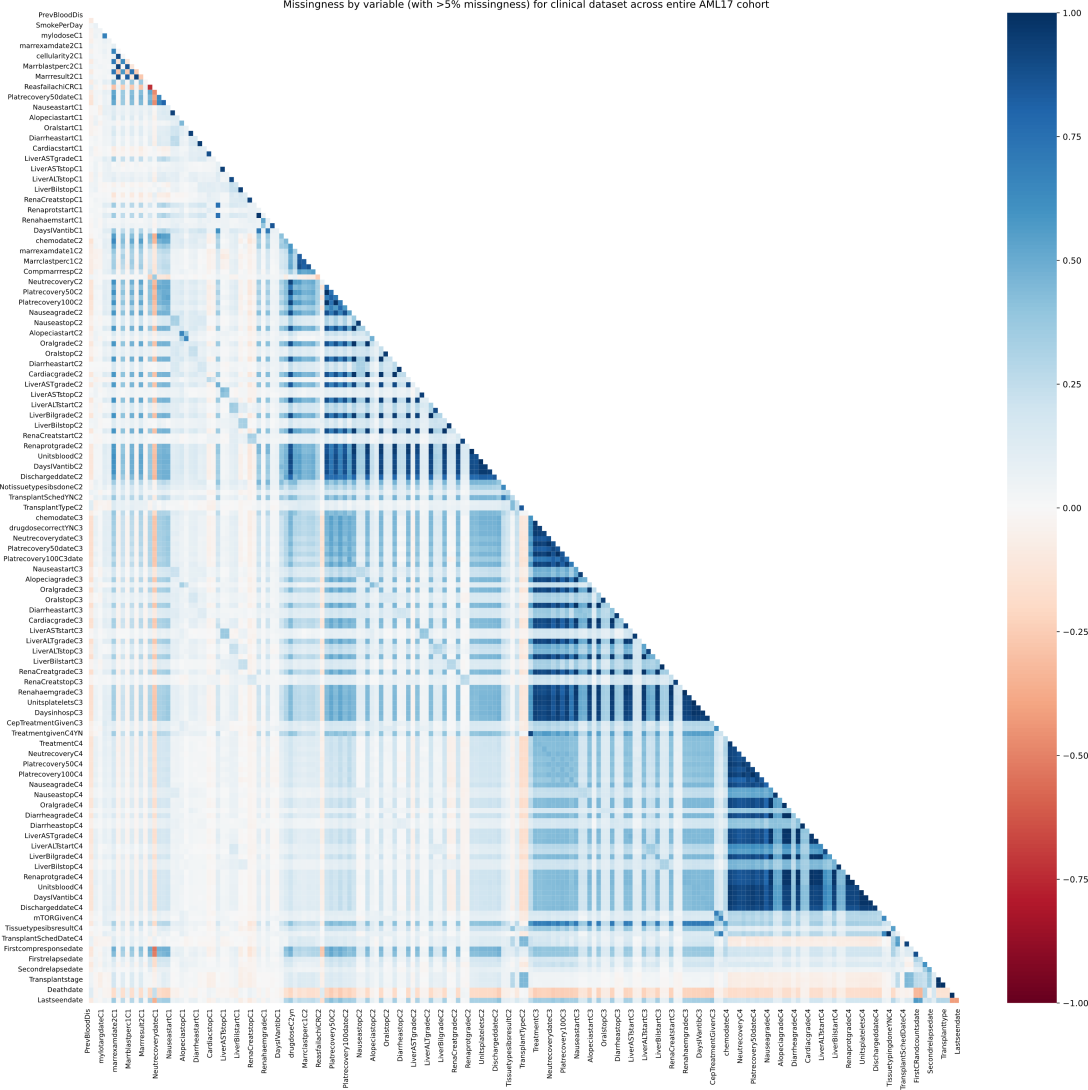
Missingness correlation per feature across the full AML17 cohort for the clinical data subset.

Across the entire AML17 cohort, the clinical subset has strong missingness correlations, suggesting a possible missing not at random mechanism for clinical data recorded at specific trial stages, indicated by the dark blue blocks of longitudinal features recorded at each trial stage in Figure 4. This is expected for any patients failing to proceed to a stage due to death or exclusion criteria.

When restricted to individual stage cohorts, the missingness correlation of clinical data shows a less pronounced block of strong positive relations, as this stage excludes patients who died beforehand (and thus have missing records) or who were otherwise no longer eligible based on trial protocols. Correlations that remain often are clinically explainable, for instance, records for comorbidity timings, such as those indicating nausea durations (“NauseastartC1” and “NauseastopC1”), are obviously both missing if the patient did not have such a symptom. However, to avoid possible assumptions, such variables, even if frequently missing across entire cohorts, were investigated for informativeness to model survival predictions by including an additional missing indicator variable for each; informativeness was then identified through stable feature importance analysis of final models illustrated in the Feature Importances section.

### Final Optimized Trial Stage Model Measurements

The highest performing models selected via c-index and feature stability selection defined in phase 3 (refer to Final Evaluation Phase in Methods section) were retrained for hyperparameter tuning under nested CV and re-evaluated according to c-index. Key statistics, such as training sample size, the ablation components, and feature set pre- and postreduction, are measured. Feature reduction was model-specific. RSF pruned features with average relative permutation importance. Nonzero feature coefficients determined by elastic net regularization remained in the CPHR model. Tables1  2 show feature reductions, best performing data source ablations, training sample sizes, and average c-index with 95% CIs of each stage-specific model. Raw JSON metric files can be found in Multimedia Appendix 11.

RSF shows higher c-index readings across all stages. However, CoxNet had smaller CIs across all stages, suggesting it may be more generalizable, which requires further analysis using an external dataset. Given that the bootstrapping across each nested fold was set to 150, which was used as a compromise between precision and computational runtimes, it may be the case that a higher number of sample iterations could yield more precise and potentially smaller intervals in both models. All recorded evaluation metrics for final models trained with nested CV are available in Multimedia Appendix 11.

**Table 2.** The highest-performing ablation components, training set sample sizes, and respective c-indices for each model after average performance from the ablation analysis phase. Subscripts below longitudinal ablation components indicate the cohort data subset is additionally constrained to only recorded prior to the respective stage.

Trial stage	Ablation components	Training sample size	Model average c-index (95% CI)
Postinduction	RSF^a^: Clinical_i_, MRD_i_^b^, NGS^c^, *FLT3^d^*, and *NPM1^e^* CoxNet^f^: Clinical_i_, *FLT3*, and *NPM1*	2989	RSF: 0.68 (0.62‐0.74) CoxNet: 0.67 (0.62‐0.72)
Post-C1	RSF: Clinical_C1_, MRD_C1_, *FLT3*, and *NPM1* CoxNet: Clinical_C1_, *FLT3*, and *NPM1*	2609	RSF: 0.69 (0.63‐0.76) CoxNet: 0.68 (0.62‐0.74)
Post-C2	RSF: Clinical_C2_, MRD_C2_, *FLT3*, and *NPM1* CoxNet: Clinical_C2_, MRD_C2_, *FLT3*, and *NPM1*	1917	RSF: 0.68 (0.61‐0.74) CoxNet: 0.66 (0.58‐0.74)
Post-C3	RSF: Clinical_C3_, MRD_C3_, *FLT3*, and *NPM1* CoxNet: Clinical_C3_, *FLT3*, and *NPM1*	761	RSF: 0.69 (0.56‐0.81) CoxNet: 0.63 (0.49‐0.77)

a RSF: random survival forest.

b MRD: minimal residual disease.

c NGS: next-generation sequencing.

d FLT3: Feline McDonough sarcoma-Like Tyrosine kinase 3.

e NPM1: Nucleophosmin 1.

f CoxNet: Cox proportional hazard regression with elastic net regularization.

### Evaluation of the AML17 Protocol Model

Using the full posttrial stage C1 cohort, the AML17 trial protocol risk assessment Cox linear regression model was compared with the RSF and CoxNet models at the same stage using a 0.9:0.1 train-test split and 1000 bootstrapped samples by c-index.

These results show the potential performance gain between trial risk-based models, such as the one used in AML17 for patient treatment stratification, and the RSF and CoxNet models used within this study. The reader is reminded that this specific protocol comparative analysis was exploratory and excluded a nested CV process, unlike those used to produce results highlighted in Table 2. The larger training sample set used for these specific models, while suggesting nonpessimistic performance with more realistic training set sizes, is at risk of overfitting against an external dataset and should be interpreted with caution. It should also be noted that CIs between all models overlap. Future work will include obtaining additional trial data from subsequent AML18 and AML19 trials as an external validation source.

### Dynamic AUC

Dynamic AUC was computed for each stage-specific model (RSF and CoxNet) from the beginning of the stage to the 5-year censoring point. Dynamic AUC quantifies a model’s discriminative ability at a given point in time *t*, representing how well the model distinguishes between patients who experience an event before *t* and those who remain event-free beyond *t*. AUC scores are bounded between values 0 and 1 inclusively, with higher values indicating better discrimination. A value of 0.5 corresponds to random chance, where a model cannot distinguish between patients. Values below 0.5 indicate a model makes predictions in the opposite ordering, effectively inverting the risk estimate. Note that an AUC of 0 indicates that the model perfectly ranks patients in the reverse order of risk; in such cases, inverting predicted risk scores would yield an AUC of 1, corresponding to perfect discrimination.

Following the ablation study, candidate models were evaluated by their mean c-index across all repeated, stratified folds of the CV process. At each trial stage, the highest performing RSF and CoxNet models were selected for a final, more robust nested CV with additional bootstrapping per fold for performance estimates. As observed, event time points differ across bootstrapping samples, a universal set of equally intervaled time points was first predefined. Each fold bootstrap sample AUC measurement was then interpolated to its nearest universal time point.

AUC shows an initial period of instability immediately after the stage’s baseline. This behavior was expected—early time points will have a smaller cumulative observed event count than the remaining time window of the trial stage. Additionally, risk distributions shift rapidly in these early time windows as patients transition into new treatments based on the existing protocol risk assessment and randomized allocation. As time progresses, all models tend to a more stabilized AUC score, shown by the plateau of the mean AUC and narrowing of CIs.

**Figure 5.**
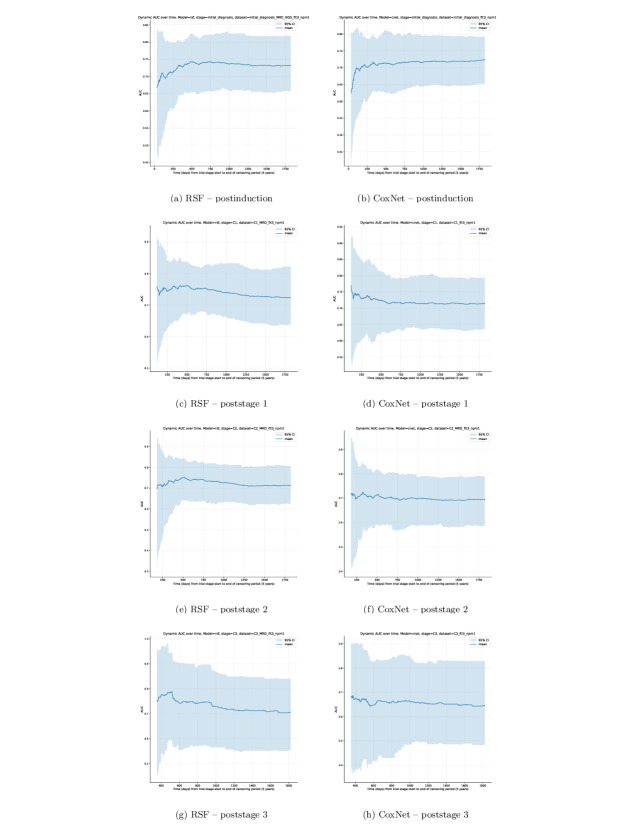
Dynamic AUC performance of each stage-specific model plotting average dynamic AUC performance as a dark blue line and 95% CI as the light blue shaded region. AUC: area under the receiver operating characteristic curve.

Later stages, (particularly poststage 3) exhibit visibly wider CIs and flatter trajectories. This reflects the considerably lower sample sizes of this stage (“Post-C3” n=761, Table 2) and higher censoring proportions (Figure 2) at this deeper trial stage. Consequently, this limits statistical power and increases uncertainty in time-dependent discrimination estimates. Therefore, dynamic AUC curves at poststage 3 (Figures 5G and 5H) should be interpreted with greater caution.

### Dynamic Brier Loss

Dynamic Brier loss was computed for each stage-specific RSF and CoxNet model selected via the ablation study to evaluate prediction accuracy over time. At each time point *t*, the Brier score measures the mean square difference between the predicted survival probability at *t* and the observed event status. Score values are bounded between 0 and 1 inclusively, where 0 represents a perfectly accurate model and 1 a completely inaccurate model.

To enable consistent comparison across CV fold bootstrapped samples, dynamic Brier loss curves were evaluated over the same set of predefined, equally intervaled time points used for the dynamic AUC assessment.

Across all stages, dynamic Brier loss scores begin with very low loss values and narrow CIs. This behavior is expected—a smaller proportion of events has been observed at these baseline windows, most patients remain event-free, resulting in highly accurate short-term predictions at low variance. As time progresses throughout each stage model, the Brier loss score increases and the CIs widen. This reflects both the increasing difficulty of long-term survival prediction and the gradual decrease of the at-risk population contributing to the estimate (as the number of patients experiencing an event before *t* increases, decreasing the remaining subset of at-risk patients available from *t*).

**Figure 6.**
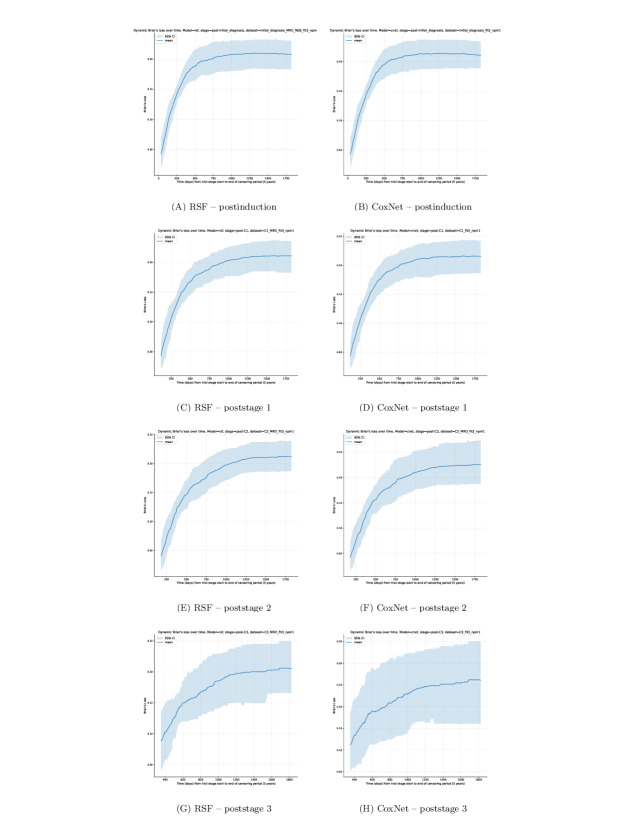
Dynamic Brier loss of each stage-specific model plotting average Brier’s loss as a dark blue line and 95% CI as the light blue shaded region.

Similarly, to dynamic AUC results, later stages (particularly poststage 3) show visibly wider CIs. This pattern arises from the reduced sample size available within the cohort (761 patients; Table 2) and higher censoring proportions (Figure 2), which limit the precision of time-dependent accuracy estimates and increase variance at later *t* evaluation points. Therefore, poststage 3 (Figure 6G and 6H) should be interpreted with caution.

Full resolution dynamic Brier loss and AUC plots are available in Multimedia Appendix 11.

### Feature Importances

The following figures show the feature importance of the best-performing tuned models at each select stage of the trial. Importance was ranked according to relative importance scores calculated from permutation importance for RSF and by coefficient values from CoxNet. For postinduction, post-C1, post-C2, and post-C3 models, RSF’s feature selection method included more features in the final model. Given the small difference in c-index and dynamic AUC performance between RSF and CoxNet during trial stage predictions, it is unlikely that the increased feature pool holds strong predictive candidates, suggesting that the increased RSF feature dimensionality from the feature selection process could pose a risk to overfitting and lack of generalizability.

**Figure 7.**
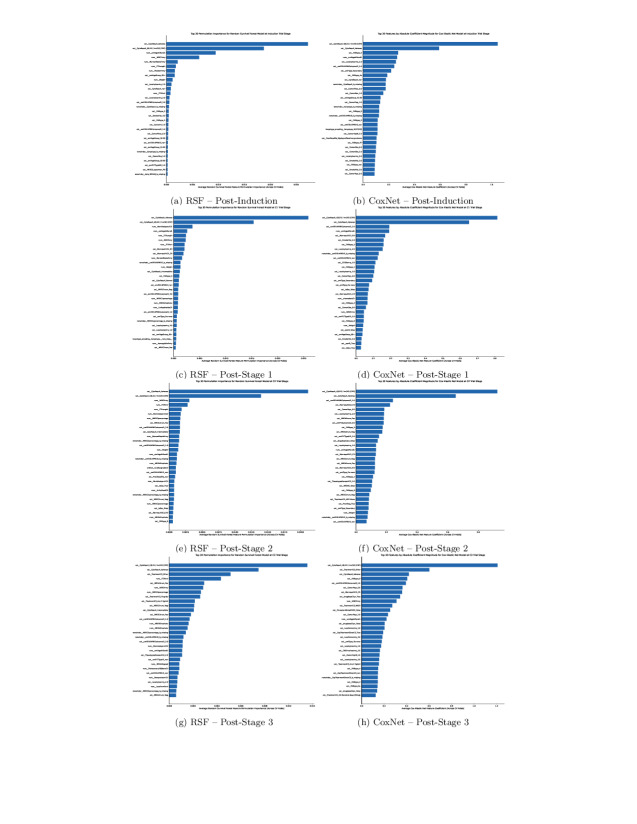
Top 30 ranked relative feature importance of trial stage specific models.

Feature importance for each trial stage model has been ranked to highlight the strongest predictors. For figure readability below, only the top 30 highest-ranked features are included; the total number of features for all stage models exceeds this number, as illustrated in Table 1. While the principal goal of this study concerns the performance of time-sequential TTE prediction models through the trial, future works investigating and explaining generalizations of these models are necessary. Therefore, as a precursory step for future work, we have highlighted the most important features of the best models for each stage in Figure 7. Features that consistently mapped over all or the majority of (often excluding the early postinduction stage as longitudinal records did not exist at this time point) trial stage models include known AML prognosticators—age, white blood cell count, marrow blast values, cytogenetic risk groups (such as the core-binding factor t(8;21)/inv(16)), *NPM1*, and *FLT3* markers [13,64,65]. There are also several consistently important predictors unreferenced as biological risk factors. Missing indicators (eg, blast marrow, *FLT3* mutation type, and internal tandem duplication length value entries) suggest predictive informativeness of missing measurements, which may reflect selection bias, measurement practices from clinical sites, or the severity of a patient’s condition. Other predictors include administrative timing fields, such as marrow blast test dates or chemotherapy timings, which, while not strictly biological risk factors, suggest the importance of treatment timing or intensity. It is possible that more precise dosage levels and treatment timings could be recommended from such a system on a per-patient basis. Predictors measured as important but not referenced within the literature most likely reflect measurement practice bias or even data leakage, not necessarily causal disease biology, and warrant further analysis.

## Discussion

### Principal Findings

The primary performance metric, c-index, of the best models for each trial stage shows that RSF outperforms CoxNet throughout all major stages of the trial, with differences in performance becoming more apparent throughout successive trial stages. This suggests the optimal simulation of TTE outcome prediction would incorporate RSF or additional nonlinear model types that can capture nonlinear, complex relationships.

Brier loss of stage models appears similar in mean square error trends for all stage models except at stage 3, likely due to having the smallest sample size for training. In the instance of stage 3, the difference in accuracy is larger between RSF and its lower accuracy counterpart, CoxNet. However, it must be noted that the sample size for training of this model is the lowest, as final trial arm branches become increasingly fractional, using 761 samples. Therefore, overfitting is increasingly more likely at this stage, and conclusions made for the outperforming model should be made with caution.

Cumulative AUC shows that both models tend to perform similarly throughout time, being most unstable at the initial stages of the model’s available prediction window before censoring (which spans from the end of the respective stage to the 5-year censoring point since patient induction). Notable inflection points occur at similar early time frame windows, which eventually plateau into a stable time-dependent prediction. The degree of change during the initial window of each stage model before stabilization appears more drastic for both models, showing instances of under- and overperformance relative to their mean AUC score. This indicates that the initial periods of all trial stage models are the most sensitive zones in terms of predictive performance. While it cannot necessarily be concluded that the large performance differences before plateaus are solely a result of inadequate sample sizes, there are naturally fewer total event instances in the earliest sample periods with respect to the aggregate of events throughout the entire 5-year window. With low event counts at the earliest AUC time points, variability may be higher, resulting in an overestimate of performance as seen across dynamic AUC plots of all stage models. This would explain the noticeable fluctuations before stabilization of the mean AUC value for each stage model. This initial window would also be the most dynamic point in time with respect to patient treatment selection, where the trial protocol outline determined the treatment stratification of patients into one of multiple arms and so was highly influential on overall survival. This initial unstable period approximately coincides with the worst-case trial length for patients from induction to the end of stage 4, roughly 230 days [16]. Aside from effective sample sizes, it seems intuitive that the most sensitive prediction period of models trained on longitudinal data would be the initial time window after their measurements, as they more accurately reflect the real state of the patient, in the sense that there has been less time for longitudinal measurements to differ from their latest recorded value.

The ablation study stresses the importance of data held in the clinical dataset as well as *FLT3* and *NPM1* and longitudinal MRD records (metadata available in Multimedia Appendices 1-3), which were used for all the highest-ranked RSF models for each stage. The differences in ablated data sources will provide contextual pointers for future analysis of model features with traditional techniques (such as survival curves) or ML techniques (such as clustering), prioritizing features ranked most important by their respective model (Figure 1). Surprisingly, the NGS subset was only included in the postinduction stage RSF model, indicating that the strongest mutational markers came from the FLT3 and NPM1 dataset and karyotype information held in the clinical dataset, both of which remained consistently important across all stage CoxNet and RSF models, as seen in Table 2. NGS mutational biomarkers remained sparse per cohort and most likely effectively acted as noise to both models. This suggests further sensitivity analysis with a wider range of available features via composite NGS feature engineering. The preprocessing of NGS data in these experiments only used available gene mutation indicators to avoid catastrophic explosions in feature set dimensionality by the creation of composite variables. Some excluded features include tumor variant allele frequency and gene mutation base start and end locations, which are candidates for future analysis. A list of all available NGS features can be found in Multimedia Appendices 1-3.

Performance analysis of models constrained to data available up to trial stages approximately equates to performances in literature for a Cox proportional hazard model used for risk group stratification based on aggregate non–time point constrained data [26]. In the wider context of digital twins [43], the results in performance in this study, particularly at the earlier stage time points, suggest that it is possible to provide accurate simulations on survival risk based on models trained at iterative stages of treatment. A digital twin would simulate multiple AML patient outcomes that are relevant to patient well-being; this invites further study on other time-based outcome predictions using the generalized method of this study, such as for QoL or comorbidity prediction.

Approaching a digital twin system with a core prediction layer using ML models should have access to longitudinal data with a temporal resolution, optimal for AML-based predictions. Given longitudinal records, such as MRD, were used by the highest performing stage models (excluding the initial postinduction stage), future work should also involve sensitivity analyses on model prediction accuracy using more frequent, intrastage measurement. This is a practically challenging area of research as trial-based data, such as AML17, even with longitudinal records, is often restricted to stage-wise updates which last several weeks or more depending on chemotherapy regimen. Such a system also demands an automated, digitalized data collection scheme, which is not the norm in trial protocols, where many records are paper-based. Currently existing ML models trained on stage-wise model, such as those shown in these experiments, indicate the promising practicality of their usage in clinical environments when used as the core stratification method within a digital twin system. However, the surrounding architecture necessary to feed models with accurate patient data with high temporal resolution is lacking. The total man-hours to preprocess trial-specific records and validate models with comparable features across trial or real-world data sources for such a system would greatly compromise its applicability, unless a standardized data capture process which records data in an ML model–friendly manner (eg, with improved error handling – particularly for critical date-time entries, mandatory classification levels instead of clinician written note fields, stricter numerical field unit standardizations, a higher volume of QoL records, and higher resolution of longitudinal record entries) is developed for patients with AML. While RSF can model complex nonlinear relationships, they do not explicitly account for sequential dependencies; when higher resolution longitudinal data are available, researchers should evaluate the performance and feasibility of ML models designed to exploit temporal structures, such as recurrent or long short-term memory neural networks.

In the context of an AML digital twin, the presented RSF and CoxNet models here would act as the core prediction layer, updating patient-specific risk estimates whenever new measurements become available. As AML data are collected at discrete protocol-defined intervals (per trial treatment stages), this framework does not make use of real-time *streamed* data (which typically do not exist in trial-based datasets) but instead “real-time upon update” recalculation of risk as new clinical or MRD entries are recorded for a patient. Generalizability across clinical sites would be supported by a standardized preprocessing pipeline like what has been developed in this study. The monitoring of feature distribution drift (eg, via Population Stability Index) would allow for precise trigger points for model retraining when necessary. The planned external validation of RSF and CoxNet models as core digital twin predictive layers on AML18 and 19 datasets will further quantify cross-site robustness.

### Run Times

Model training and validation were computed on the Cardiff University “Hawk” high-performance computing cluster. Jobs were submitted via Simple Linux Utility for Resource Management, using 1 node, 1 task, and 14 CPU cores with the high-throughput partition. Processing time measurements show that RSF is much more intensive to validate than CoxNet, primarily due to the time complexity of the exhaustive feature importance method used—permutation importance. For example, when comparing runtimes of the largest cohort and ablation set—postinduction stage, with ablation components (clinical, MRD, NGS, *FLT3*, and *NPM1*)—averaged across the 3 repeated, k=5 split CV loop, RSF took on average 147 seconds per fold, while CoxNet on average took 1 second. This difference is made more apparent after the more robust internal nested CV process, where the same RSF and CoxNet models and ablations took 7.9 hours and 1.4 hours, respectively, for the *entire* validation process across all folds. A caveat arises, particularly with the usage of the RSF model in clinical applications with large sample sizes; while predictions from trained models are near instantaneous, the full training and validation times of such models may be costly for critical patients relying on fast treatment delivery. In practice, it may be necessary to use CoxNet (or a similar “fast” baseline model) for predictions and counterfactual simulations as a preliminary tool while more sensitive, albeit slower models, such as RSF, are trained. With respect to identifying when models should be retrained, a quantifiable approach to determine the threshold should be calculated. For example, the Population Stability Index can be used to measure if there is a significant drift in a feature between the reference (trained) sample set and the new dataset. This would minimize redundancy and processing constraints in retraining by otherwise using an arbitrary threshold for determining when a model should be retrained, in turn minimizing potential performance or generalizability loss.

### Comparisons With Previous Work

Using a Cox proportional hazards model with ridge regression, Tazi et al [26] recorded an IPCW c-index of approximately 0.7 with a combined total of 26 clinical, demographic, FLT3 (ITD), and other molecular class features aggregated from UK-NCRI, such as AML11, 12, 14, 15, 16, and 17 trials. Models constrained to data at each trial stage in this study have comparable IPCW c-index scores of around 0.69 using RSF. The number of features retained in this study after model reduction processes is variable per stage, highlighting the significance of specific features at select time points for TTE predictions. Many features consistently shared across stages are noted in existing ELN risk classification, and those not referenced indicate the potential importance of timing and informativeness from both administrative and testing fields. The total number of retained features is higher for each stage model than in the study by Tazi et al [26], which used 26 features, as opposed to over 160 features across all RSF stage models. Future analysis will involve all features used at each stage in comparison with existing literature and guidelines for risk stratification, in addition to external validation to assess model generalizability.

RSF and CoxNet models outperform the AML17 protocol’s Cox model for risk stratification based on c-index (“Evaluation of AML17 Protocol Model” in Table 3, showing c-index readings of AML17 Protocol Cox Linear Regression=0.66, CoxNet=0.68, and RSF=0.70), suggesting both implementations can more accurately predict TTE. However, CIs overlap, suggesting larger datasets may be necessary to conclude an absolute improvement in generalized performance.

**Table 3.** C-indices for each model trained on a larger cohort sample size of data available from post stage.

Model	C-index (95% CI)
AML17^a^ Protocol Cox Linear Regression	0.66 (0.63‐0.68)
CoxNet^b^	0.68 (0.63‐0.73)
RSF^c^	0.70 (0.64‐0.76)

a AML17: United Kingdom National Cancer Research Institute Acute Myeloid Leukaemia 17 randomized controlled trial.

b CoxNet: Cox proportional hazard regression with elastic net regularization.

c RSF: random survival forest.

### Limitations

Since the completion of the AML17 trial, substantial progress has been made in patient therapy options through the identification of prognostic, predictive, and targetable molecular abnormalities [19]. While the analysis of models used in this study provides insight into survival prediction performance using longitudinally restricted data, future work would benefit from the inclusion of more recent trial datasets using newly approved first-line treatment options, such as Midostaurin and CPX-351. Additionally, AML17 patient-reported outcomes, including QoL, were excluded from model training due to concerns on overall sample size within trial stage time points. The future inclusion of datasets with larger pools of patient-reported outcomes data is of particular interest for the prediction of additional outcome responses that may reflect on the social and psychological health of patients at different stages of disease treatment and progression. Further research into additional outcome predictions can be integrated as part of a generalized AML digital twin that can inform patients and provide accurate recommendations to health care practitioners, particularly where survival risk between treatments is marginal, but there are clear differences in secondary predictions, such as patients’ QoL.

### Conclusion

This study shows the practicality of time-to-survival-event predictions when training sets of CoxNet and RSF models, which are sequentially constricted to data measured up to the end of respective AML17 trial stages. The performance of these sequential TTE models is intended to justify their use as part of a wider digital twin system simulating multiple TTE outcomes for patients with AML. The primary c-index metric shows comparable scores to the literature that uses similar models on aggregate sets of similar trial data. Consistent and stable important features relative to each stage-specific model are supported by ELN literature on AML classification, and additional nonreferenced predictors suggest the importance of stage-specific administrative and timing fields. Additional cumulative-dynamic AUC and Brier loss metrics have been provided. The most immediate future work includes feature analysis of the best models at each stage, further comparison with existing risk group stratification guidelines such as ELN, external validation with follow-up AML18 and 19 trial programs, the implementation of a minimally adapted pipeline for different outcome measurements, and the inclusion of patient self-assessed QoL form records alongside a broader collection of longitudinal MRD data, which have been recorded after trial treatment stages as detailed in the AML17 protocol and follow-up AML18 and 19 trials.

## Supplementary material

10.2196/75678 Multimedia Appendix 1 Clinical data dictionary.

10.2196/75678 Multimedia Appendix 2 MRD data dictionary.

10.2196/75678 Multimedia Appendix 3 FLT3NPM1 data dictionary.

10.2196/75678 Multimedia Appendix 4 NGS metadata.

10.2196/75678 Multimedia Appendix 5 Feature exclusion summary.

10.2196/75678 Multimedia Appendix 6 Cox proportional hazard regression and regularization techniques.

10.2196/75678 Multimedia Appendix 7 Random survival forest.

10.2196/75678 Multimedia Appendix 8 All relevant Python files used for preprocessing, building, and validation of RSF/CoxNet models.

10.2196/75678 Multimedia Appendix 9 Feature importance values and c-index scores for each ablated model across the repeated k-fold cross-validation from the ablation study phase.

10.2196/75678 Multimedia Appendix 10 Missingness correlation per feature heatmaps and missingness frequency bar charts across AML17 treatment stage cohorts for each data source subset used to train RSF and CoxNet models.

10.2196/75678 Multimedia Appendix 11 Contains (1) feature importance results for final treatment stage-wise model builds both pre- and postfeature reduction, (2) run-time logs of final CoxNet and RSF models for each fold of nested cross-validation, (3) full resolution dynamic AUC and dynamic Brier loss plots for final stage models, and (4) A JSON file of average values across all bootstrapped samples of each fold for all evaluation metrics: c-index, IPCW c-index, dynamic Brier loss, and dynamic AUC.

## Abbreviations

AML: acute myeloid leukemia
AML17: United Kingdom National Cancer Research Institute Acute Myeloid Leukaemia 17 randomized controlled trial
AUC: area under the receiver operating characteristic curve
c-index: concordance index
CoxNet: Cox proportional hazard regression with elastic net regularization
CPHR: Cox proportional hazard regression
CV: cross-validation
ELN: European LeukemiaNet
FLT3: Feline McDonough sarcoma-Like Tyrosine kinase 3
IPCW: inverse probability of censoring weights
ML: machine learning
MRD: minimal residual disease
NGS: next-generation sequencing
NPM1: Nucleophosmin 1
QoL: quality of life
RSF: random survival forest
TTE: time-to-event
UK-NCRI: United Kingdom National Cancer Research Institute
WHO: World Health Organization
