# Supplementary material for: Random Survival Forest Versus Elastic-Net Regularized Cox Regression for Survival Prediction in Acute Myeloid Leukemia at Distinct Treatment Time Points: Model Performance Comparison Study
Source: JMIR Bioinform Biotechnol. 2026 Apr 29;7:e75678. doi: 10.2196/75678 (PMC13128161; doi:10.2196/75678)
Supplement: Multimedia Appendix 4 [file bioinform-v7-e75678-s004.docx]

A metadata description of the NGS mutation profile dataset is not yet available, a brief list outlining the features are as follows:

1. Mutation type (Deletion, substitution, insertion, partial tandem duplications, and insertion-deletions): **String**
2. Chromosome: **String**
3. Mutation base start index: **Integer**
4. Mutation base end index: **Integer**
5. Wild type (wt), the "“standard"” base sequence occurring in a normal population at the mutant locus identified: **String**
6. Mutant type (mt), the mutated variation of wt: **String**
7. Mutant gene: **String**
8. Mutant protein: **String**
9. Tumor variant allele frequency: **Integer**
10. Tumor mutation base sequence depth: **Integer**
