# Supplementary material for: Random Survival Forest Versus Elastic-Net Regularized Cox Regression for Survival Prediction in Acute Myeloid Leukemia at Distinct Treatment Time Points: Model Performance Comparison Study
Source: JMIR Bioinform Biotechnol. 2026 Apr 29;7:e75678. doi: 10.2196/75678 (PMC13128161; doi:10.2196/75678)
Supplement: Multimedia Appendix 6 [file bioinform-v7-e75678-s006.docx]

## Cox Proportional Hazard Regression and Regularisation Techniques

Cox Proportional Hazard Regression is a semi-parametric model which constructs a hazard function for a patient at a given point in time:

$$h\left( t_{i}|X_{i} \right)=h_{0}\left( t_{i} \right)\exp\left( X_{i}\beta\right)$$

Where, $h\left( t_{i}|X_{i} \right)$ is the overall hazard function of a patient at time-point $t_{i}$ given a set of covariates (feature variables), $X_{i}$. $h_{0}\left( t_{i} \right)$ is the baseline hazard function, which is not explicitly estimated but assumed as the baseline risk of a patient at a given point in time if all covariates $X_{i}$ are zero. $\exp\left( X_{i}\beta\right)$ measures the risk (relative to the baseline hazard function) of each covariate according to their respective coefficient from the vector of coefficients $\beta$.

Although popular in survival analysis, traditional Cox Proportional Hazard Regression models can fail to converge when a large matrix of feature variables contains collinearity. Regularisation methods are common practice to attenuate this problem [1] by iteratively reducing coefficient values. "Elastic Net" [2] is one such method of a "penalised" Cox model. Elastic Net applies two penalising constraints to the coefficient values of features in the Cox model by using two penalty terms from separate regularisation techniques:

- LASSO, $\mathcal{l}_{1}$, shrinks coefficients to zero, effectively acting as feature selection by removing non-important features. However, this approach may result in the selection of just one feature from a set of highly correlated features arbitrarily which in turn may reduce model interpretability and performance [1].
- Ridge, $\mathcal{l}_{2}$, alternatively, shrinks coefficients *towards* but not including zero, retaining all feature variables whilst avoiding collinearities causing model failure in convergence. However, the retaining of feature variables can negatively affect model performance by including non-predictive features, making the model overly-complex [1]**.**

Elastic Net combines both $\mathcal{l}_{1}$ and $\mathcal{l}_{2}$ where penalty strengths can be iteratively tuned according to model performance (via c-index) to find an ideal ratio for performance:

$$\text{Penalty}=\lambda_{1}\sum_{j=1}^{p} \left| \beta_{j} \right|+\lambda_{2}\sum_{j=1}^{p} \beta_{j}^{2}$$

Where, $\beta_{j}$ is the coefficient of a feature. $\lambda_{1}$ controls the degree of the Ridge Regression penalty and $\lambda_{2}$ controls the degree of the LASSO penalty. $p$ is the total number of features.

Benner et. Al [1] provides an introduction and detailing of the methodology for LASSO, Ridge, and Elastic Net, as well as several advantages and disadvantages of each in application.

## Bibliography

[1] ‘High‐Dimensional Cox Models: The Choice of Penalty as Part of the Model Building Process - Benner - 2010 - Biometrical Journal - Wiley Online Library’. Accessed: Oct. 12, 2024. [Online]. Available: https://onlinelibrary.wiley.com/doi/10.1002/bimj.200900064

[2] H. Zou and T. Hastie, ‘Regularization and Variable Selection Via the Elastic Net’, *J. R. Stat. Soc. Ser. B Stat. Methodol.*, vol. 67, no. 2, pp. 301–320, Apr. 2005, doi: 10.1111/j.1467-9868.2005.00503.x.
