# Supplementary material for: Random Survival Forest Versus Elastic-Net Regularized Cox Regression for Survival Prediction in Acute Myeloid Leukemia at Distinct Treatment Time Points: Model Performance Comparison Study
Source: JMIR Bioinform Biotechnol. 2026 Apr 29;7:e75678. doi: 10.2196/75678 (PMC13128161; doi:10.2196/75678)
Supplement: Multimedia Appendix 7 [file bioinform-v7-e75678-s007.docx]

## Random Survival Forest

Random Survival Forests are an adaptation of Breiman’s Random Forest algorithm [1] which can use several splitting rules within the binary decision tree construction phase. This Sci-Kit library implementation [2] used the log-rank splitting rule. The log-rank splitting rule determines the cut-off threshold value at an individual node such that the survival differences between its left and right child nodes are maximized. The log-rank rule succinctly described by Liu et. Al [3] is as follows:

$$L\left( X_{j},c \right)=\frac{\sum_{k=1}^{K} \left( d_{k,l}-Y_{k,l}\frac{d_{k}}{Y_{k}} \right)}{\sqrt{\sum_{k=1}^{K} \frac{Y_{k,l}}{Y_{k}}\left( 1-\frac{Y_{k,l}}{Y_{k}} \right)\left( \frac{Y_{k}-d_{k}}{Y_{k}-1} \right)d_{k}}}$$

Where, $d_{k}$ is the number of deaths at time-point $k$ of the parent node. $Y_{k}$, the number of patients "at-risk" (patients who are alive) at time-point $k$. $Y_{k,l}$ and $d_{k,l}$ represent the partition of dead and at-risk patients respectively at time-point $k$ that are fed into the left child node. $X_{j}$ is a randomly selected feature vector from a set of candidate features, $p$ (the size of $p$ is determined during hyper-parameter tuning; the RandomSurvivalForest implementation used defaults $p$ to the total number of features in the training set). $c$ is the cut-off value determining what patient proceeds into left and right child nodes according to the value of $X_{j}$ at time point $k$. The objective is to find the value $c^{*}$ and feature $X_{j}^{*}$ that maximises the difference in proportional patient survival of those partitioned between left and right nodes.

An in-depth explanation of Random Forest and the Random Survival Forest adaptation for time-to-event predictions are explained by Liu et. Al [3].

## Bibliography

[1] L. Breiman, ‘Random Forests’, *Mach. Learn.*, vol. 45, no. 1, pp. 5–32, Oct. 2001, doi: 10.1023/A:1010933404324.

[2] ‘sksurv.ensemble.RandomSurvivalForest — scikit-survival 0.23.0’. Accessed: Oct. 12, 2024. [Online]. Available: https://scikit-survival.readthedocs.io/en/v0.23.0/api/generated/sksurv.ensemble.RandomSurvivalForest.html

[3] Y. Liu, S. Zhou, H. Wei, and S. An, ‘A comparative study of forest methods for time-to-event data: variable selection and predictive performance’, *BMC Med. Res. Methodol.*, vol. 21, no. 1, Art. no. 1, Dec. 2021, doi: 10.1186/s12874-021-01386-8.
