# Supplementary figures and images for: Random Survival Forest Versus Elastic-Net Regularized Cox Regression for Survival Prediction in Acute Myeloid Leukemia at Distinct Treatment Time Points: Model Performance Comparison Study
Source: JMIR Bioinform Biotechnol. 2026 Apr 29;7:e75678. doi: 10.2196/75678 (PMC13128161; doi:10.2196/75678)

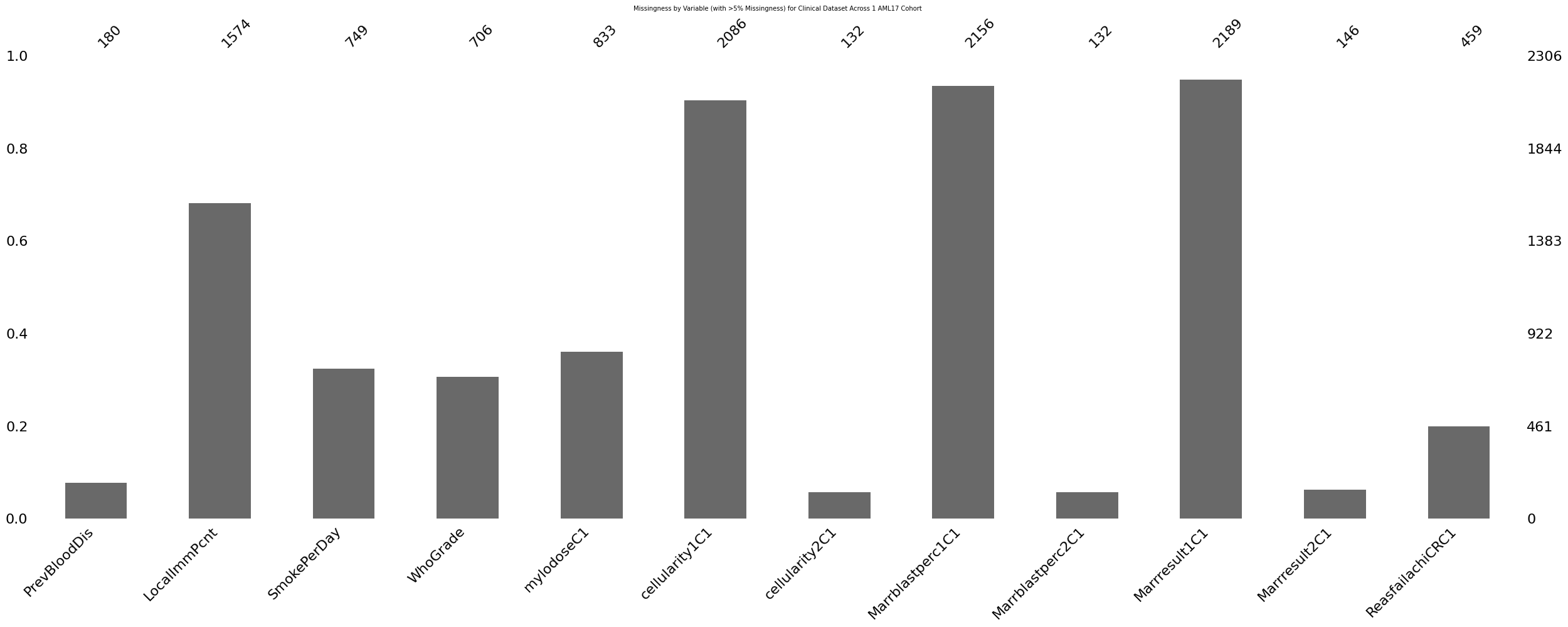

Supplement: Multimedia Appendix 10 [file bioinform-v7-e75678-s010.zip › missingness_visualisation/C1/c1_missingness_Clinical_bar.png]

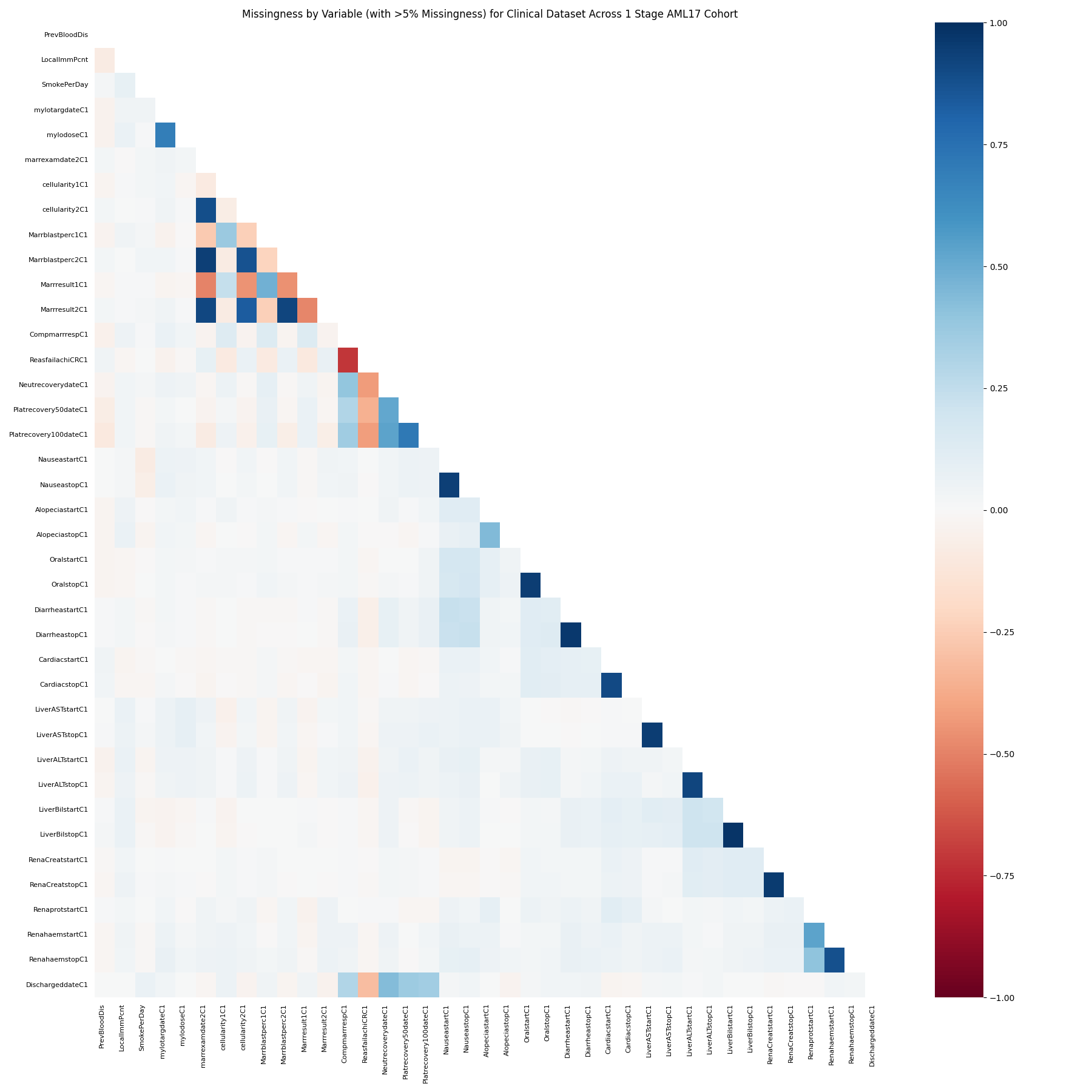

Supplement: Multimedia Appendix 10 [file bioinform-v7-e75678-s010.zip › missingness_visualisation/C1/c1_missingness_Clinical_heatmap.png]

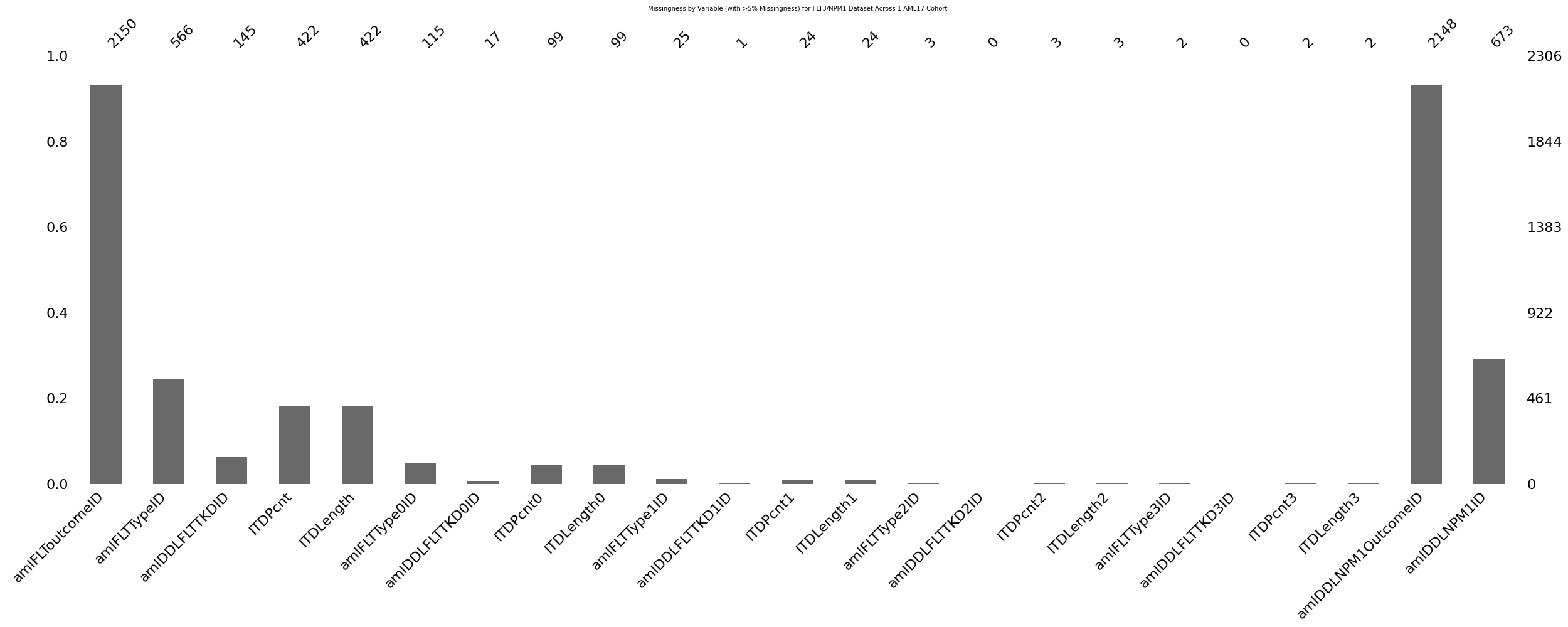

Supplement: Multimedia Appendix 10 [file bioinform-v7-e75678-s010.zip › missingness_visualisation/C1/c1_missingness_FLT3_NPM1_bar.png]

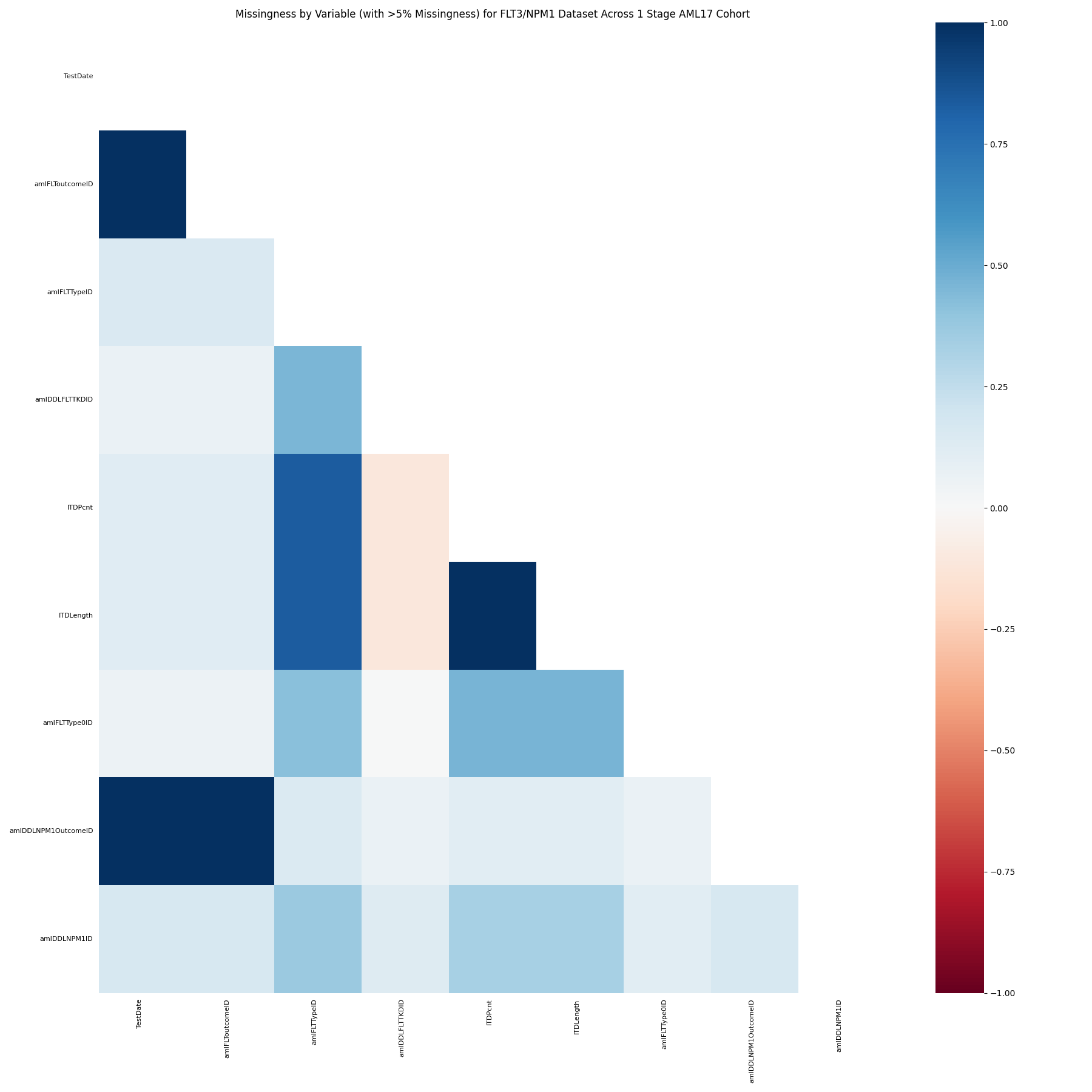

Supplement: Multimedia Appendix 10 [file bioinform-v7-e75678-s010.zip › missingness_visualisation/C1/c1_missingness_FLT3_NPM1_heatmap.png]

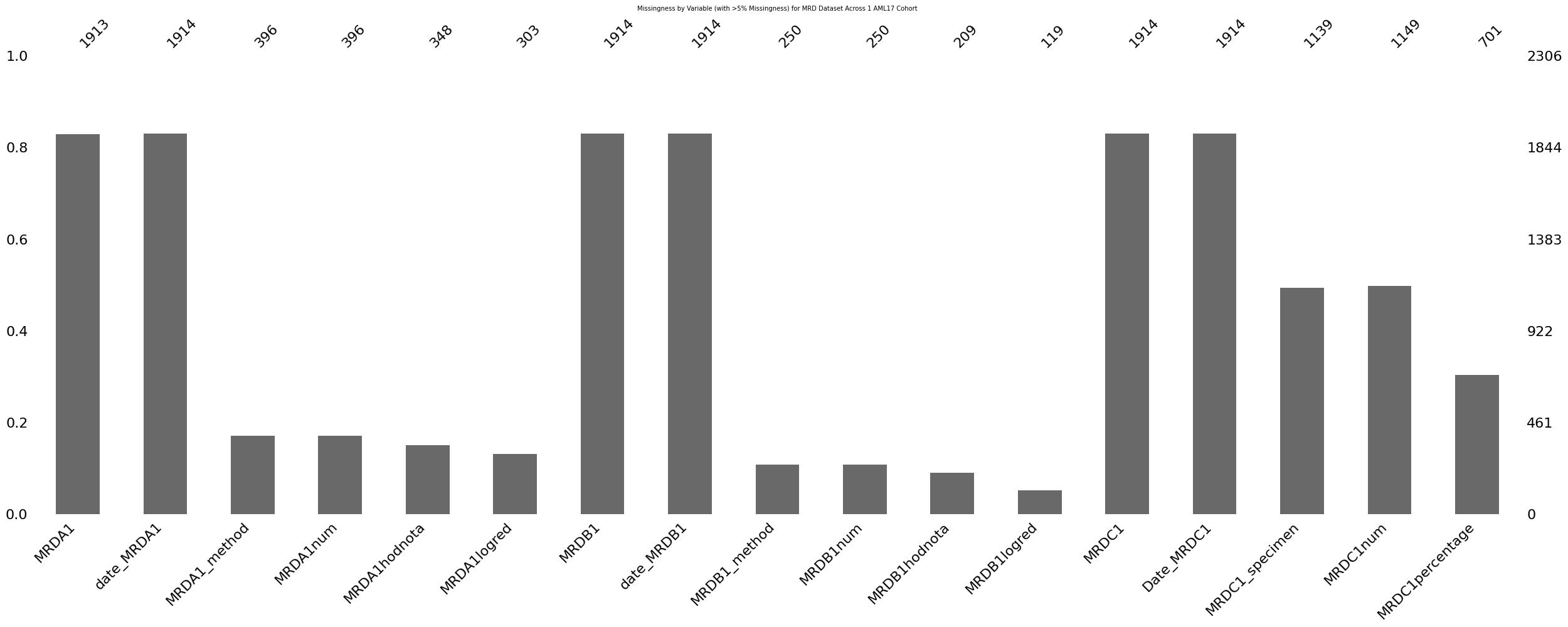

Supplement: Multimedia Appendix 10 [file bioinform-v7-e75678-s010.zip › missingness_visualisation/C1/c1_missingness_MRD_bar.png]

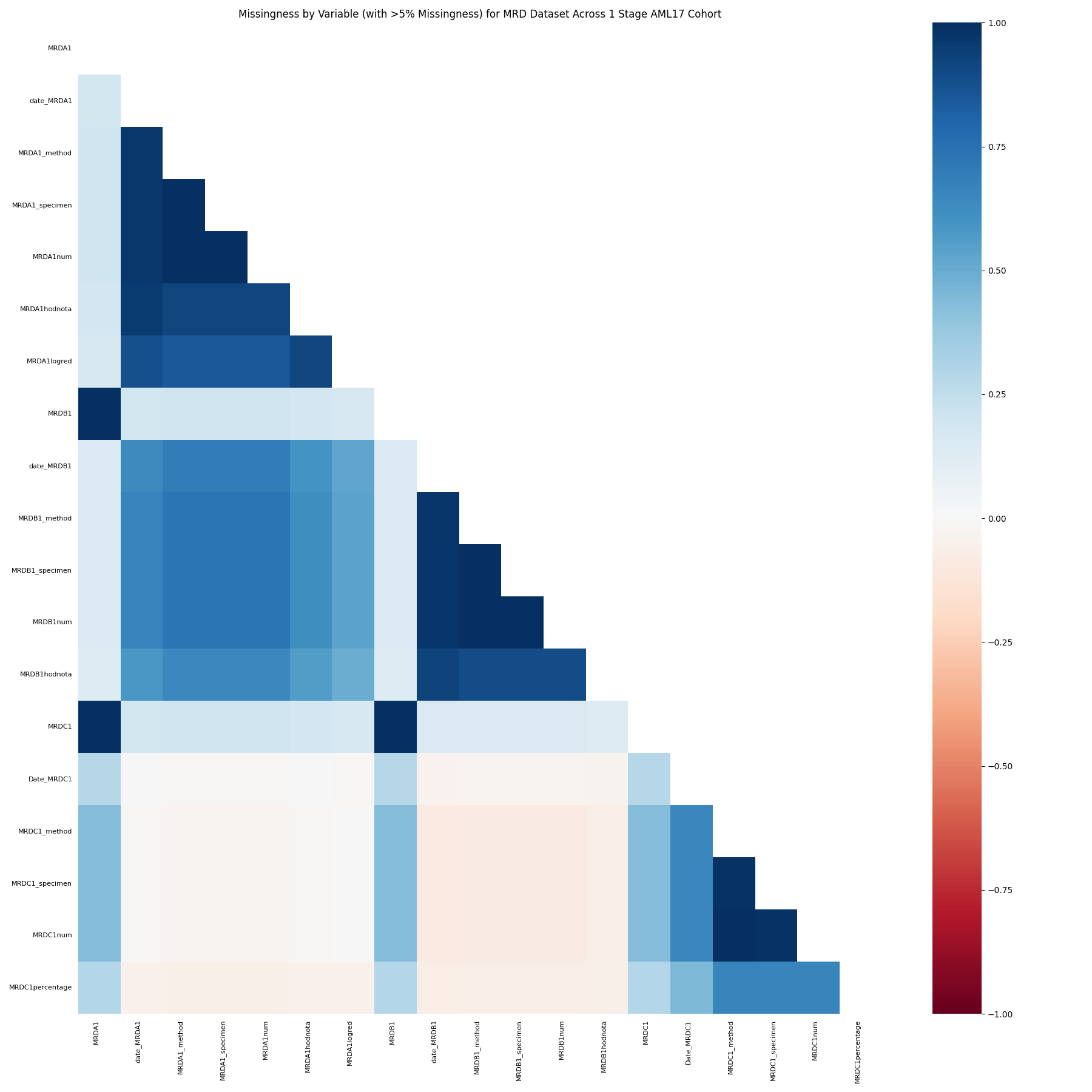

Supplement: Multimedia Appendix 10 [file bioinform-v7-e75678-s010.zip › missingness_visualisation/C1/c1_missingness_MRD_heatmap.png]

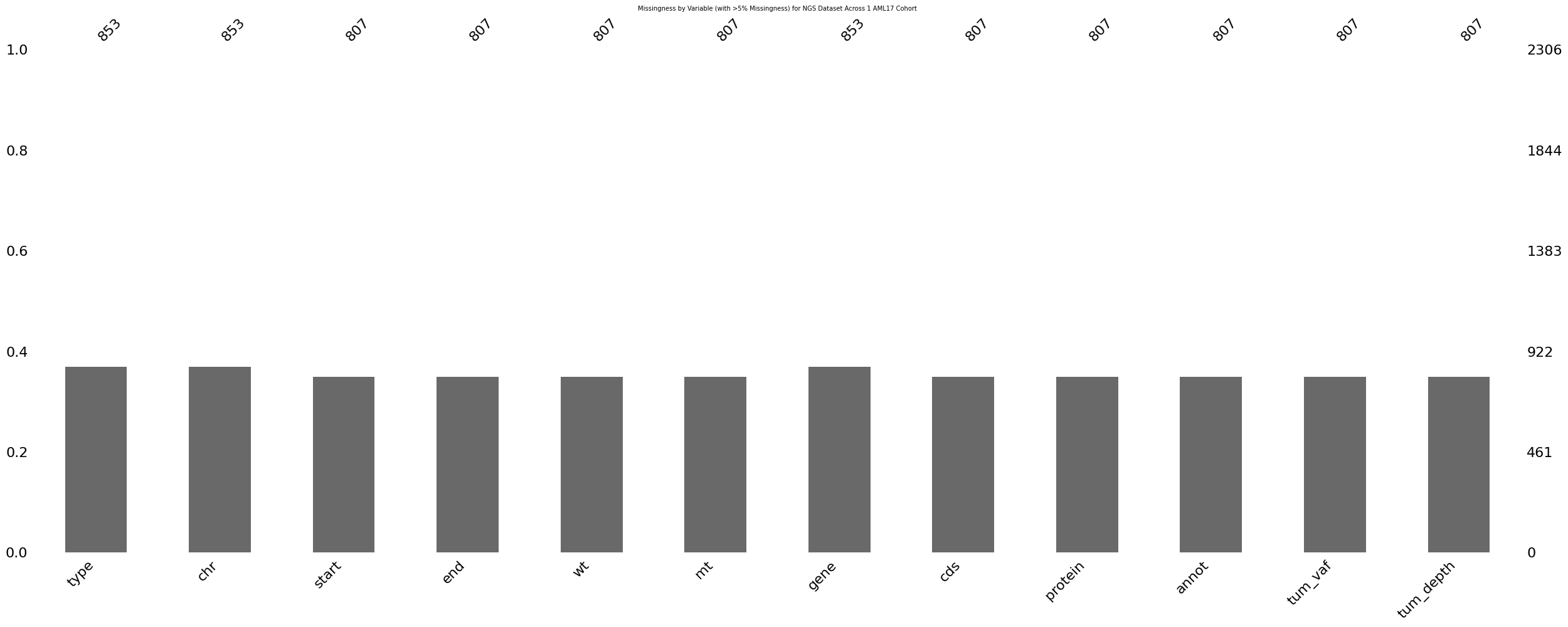

Supplement: Multimedia Appendix 10 [file bioinform-v7-e75678-s010.zip › missingness_visualisation/C1/c1_missingness_NGS_bar.png]

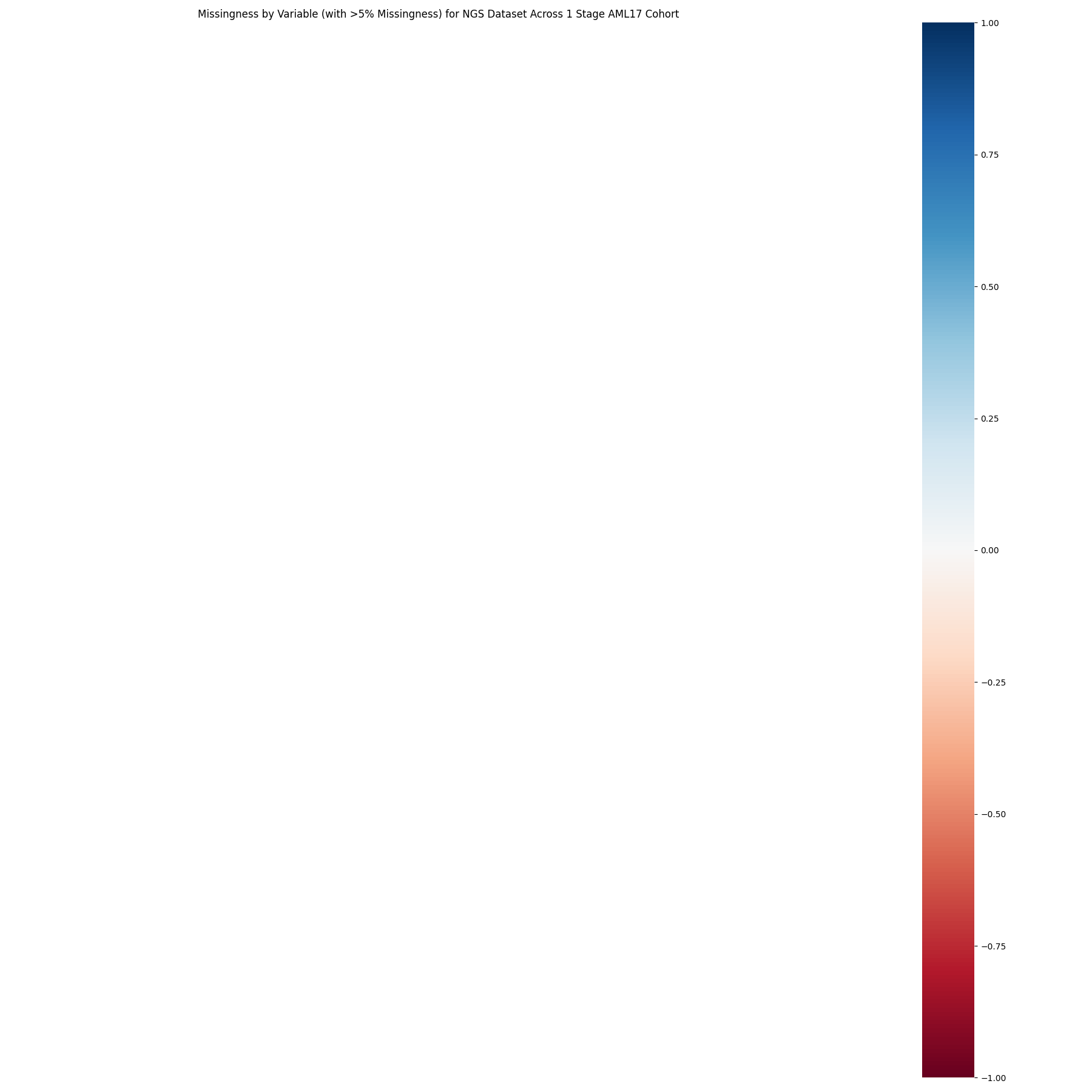

Supplement: Multimedia Appendix 10 [file bioinform-v7-e75678-s010.zip › missingness_visualisation/C1/c1_missingness_NGS_heatmap.png]

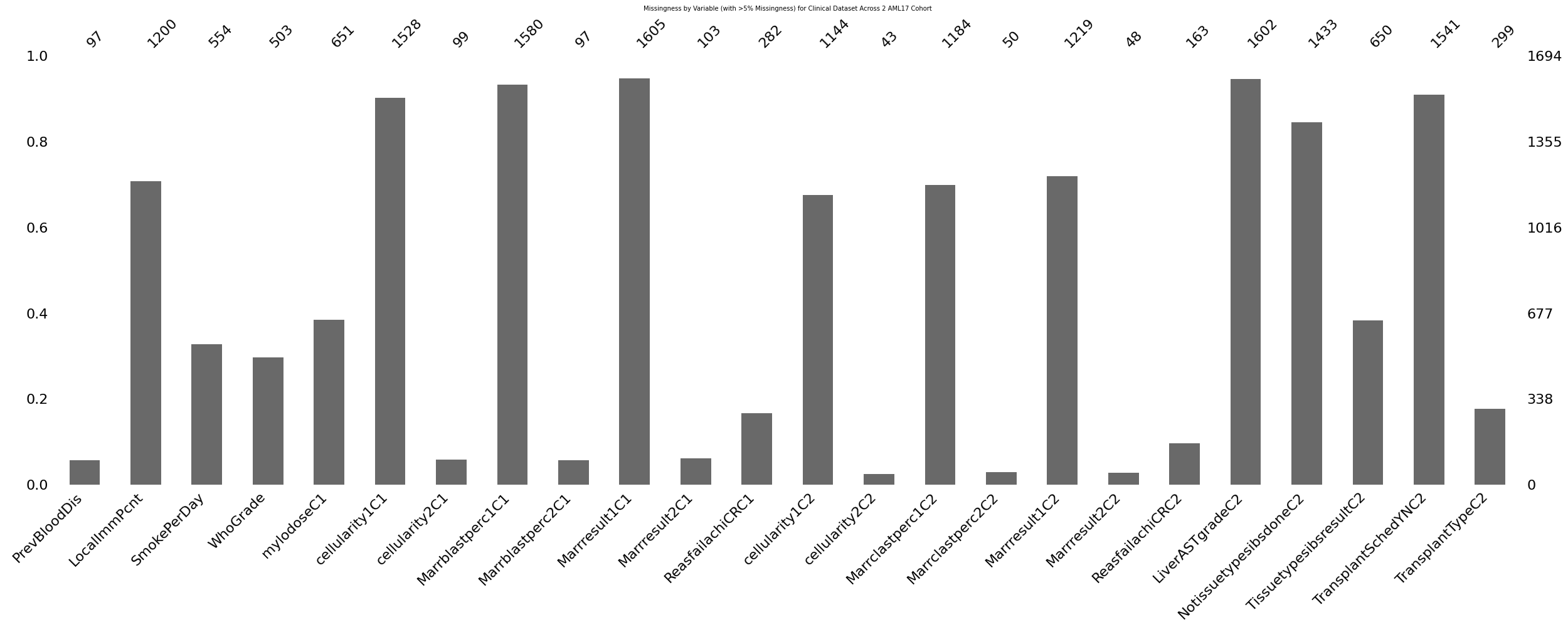

Supplement: Multimedia Appendix 10 [file bioinform-v7-e75678-s010.zip › missingness_visualisation/C2/c2_missingness_Clinical_bar.png]

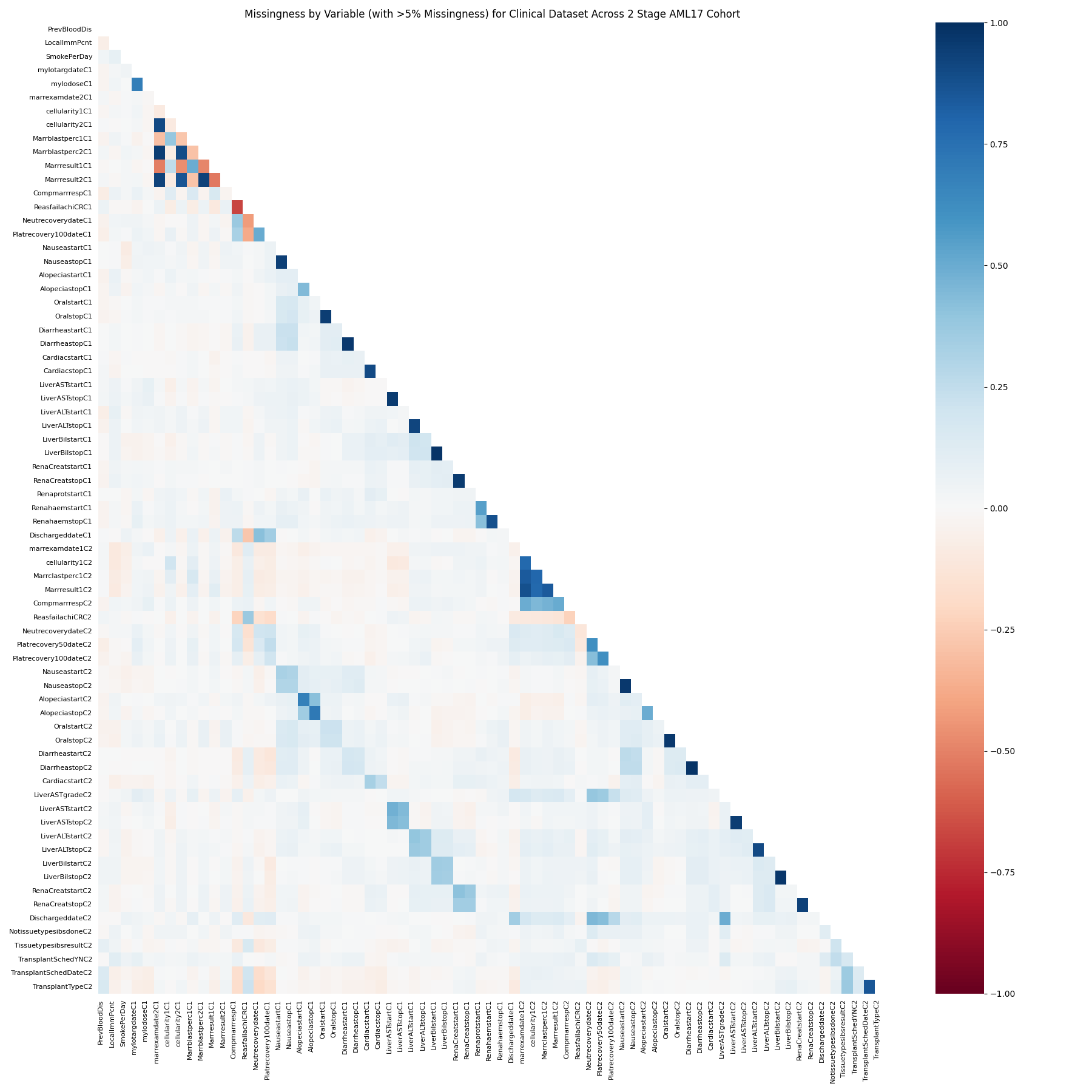

Supplement: Multimedia Appendix 10 [file bioinform-v7-e75678-s010.zip › missingness_visualisation/C2/c2_missingness_Clinical_heatmap.png]

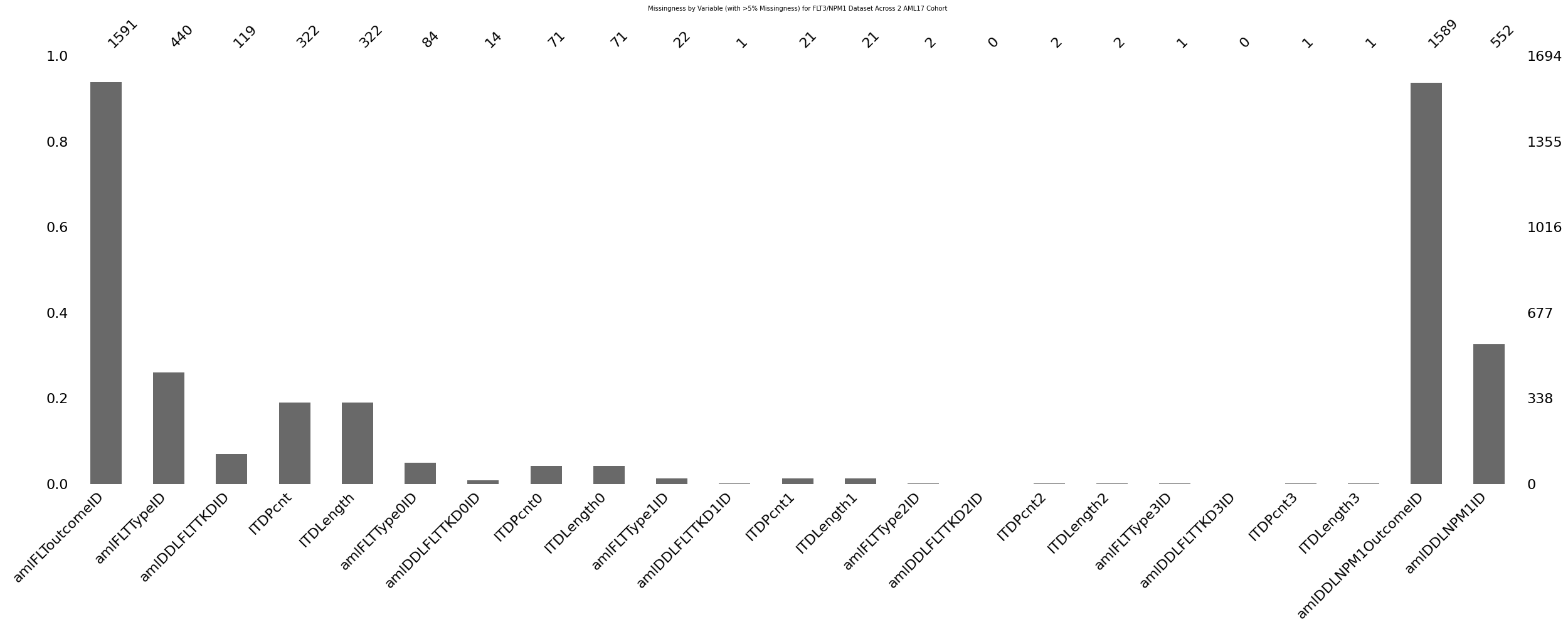

Supplement: Multimedia Appendix 10 [file bioinform-v7-e75678-s010.zip › missingness_visualisation/C2/c2_missingness_FLT3_NPM1_bar.png]

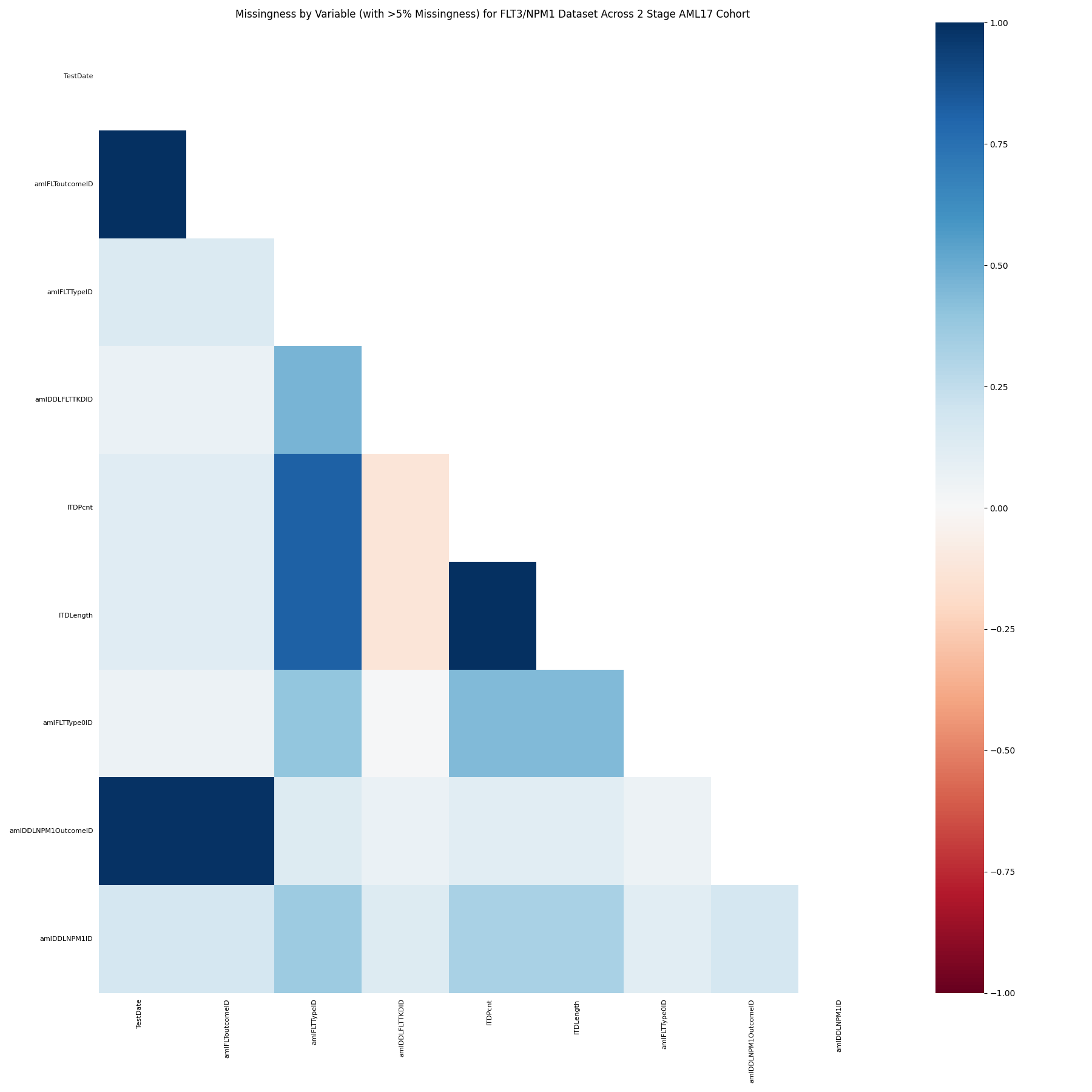

Supplement: Multimedia Appendix 10 [file bioinform-v7-e75678-s010.zip › missingness_visualisation/C2/c2_missingness_FLT3_NPM1_heatmap.png]

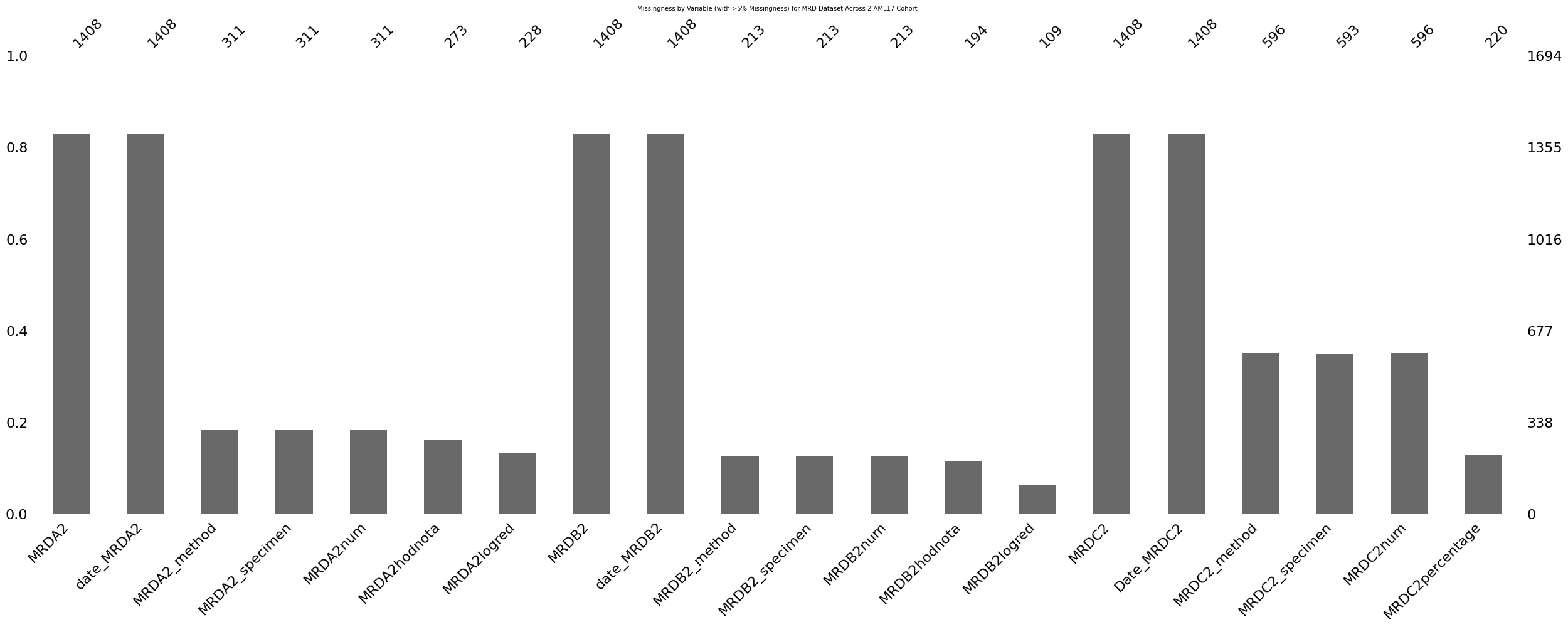

Supplement: Multimedia Appendix 10 [file bioinform-v7-e75678-s010.zip › missingness_visualisation/C2/c2_missingness_MRD_bar.png]

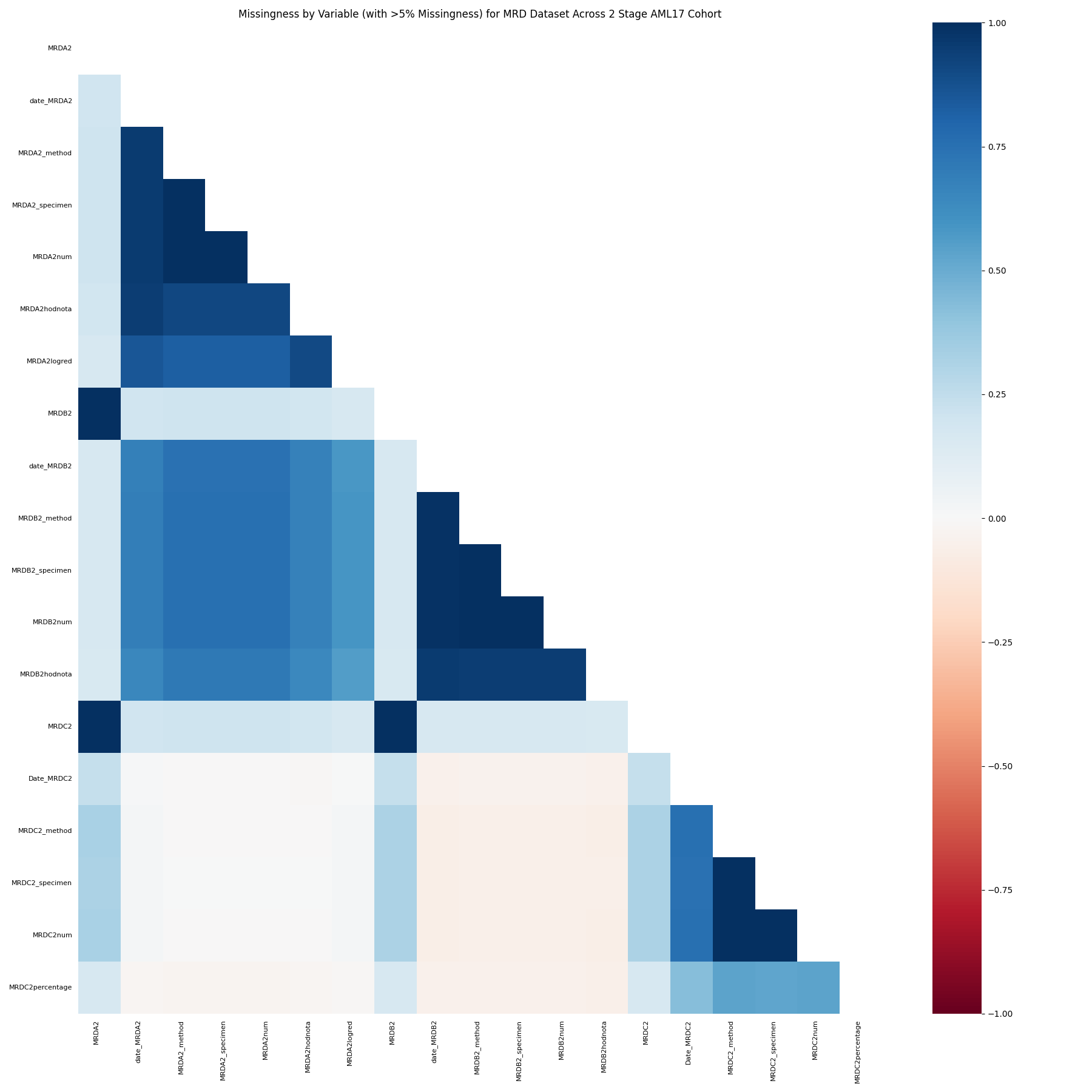

Supplement: Multimedia Appendix 10 [file bioinform-v7-e75678-s010.zip › missingness_visualisation/C2/c2_missingness_MRD_heatmap.png]

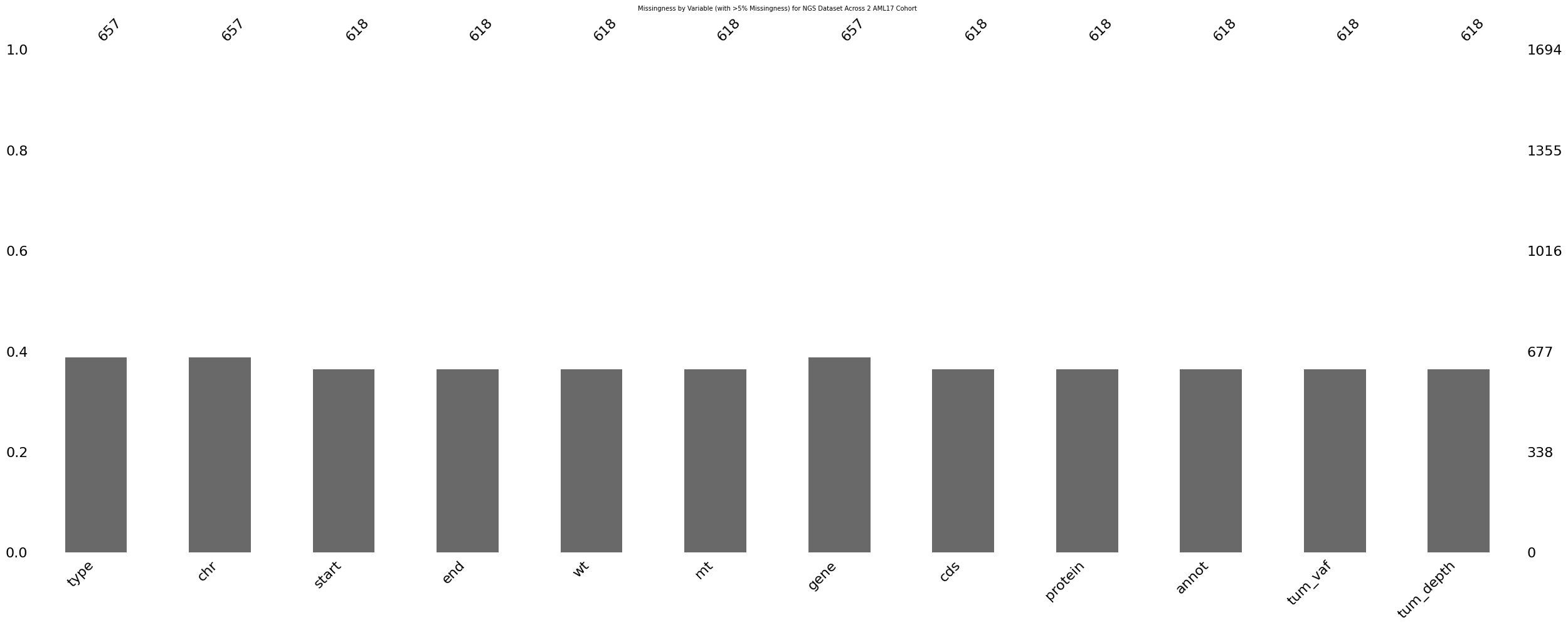

Supplement: Multimedia Appendix 10 [file bioinform-v7-e75678-s010.zip › missingness_visualisation/C2/c2_missingness_NGS_bar.png]

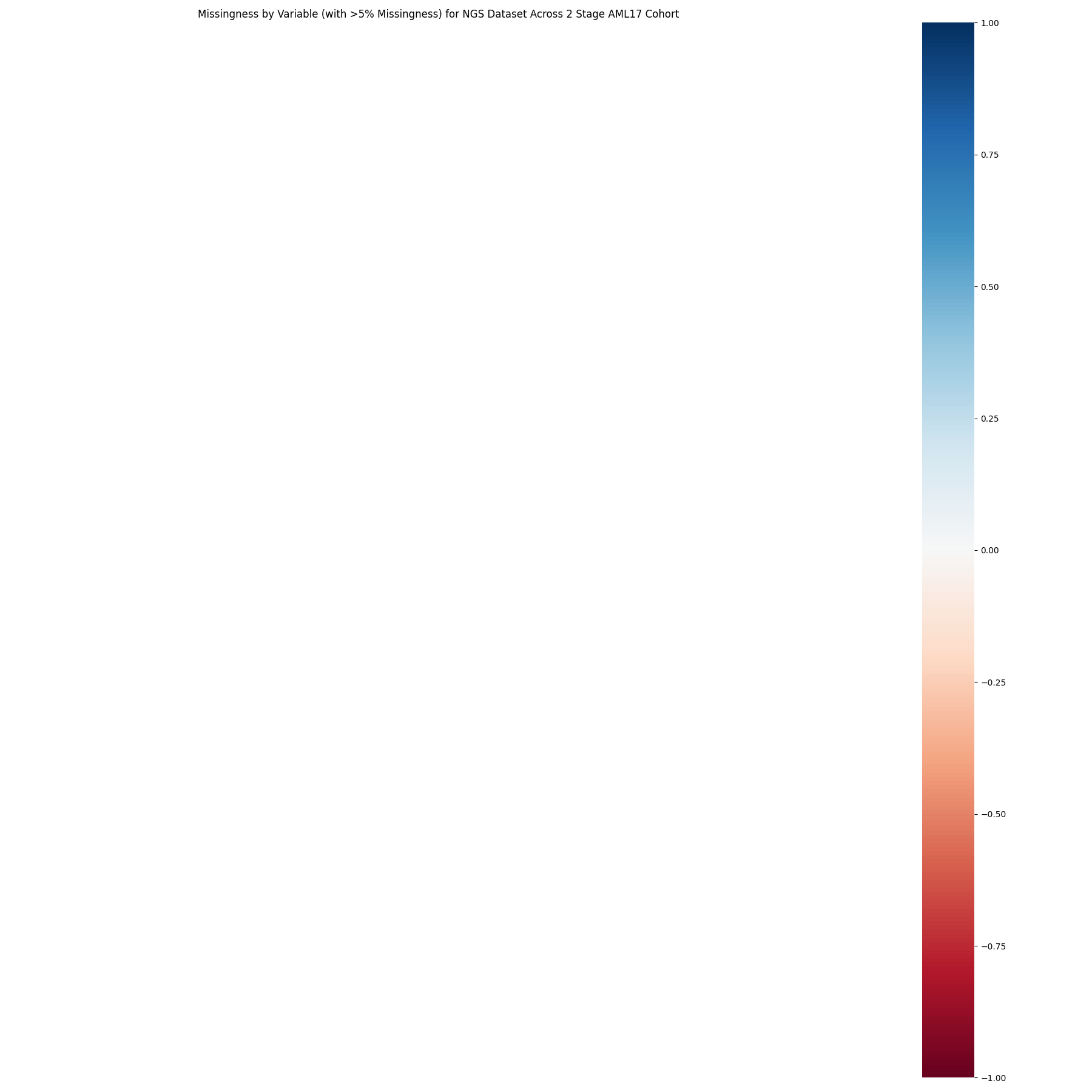

Supplement: Multimedia Appendix 10 [file bioinform-v7-e75678-s010.zip › missingness_visualisation/C2/c2_missingness_NGS_heatmap.png]

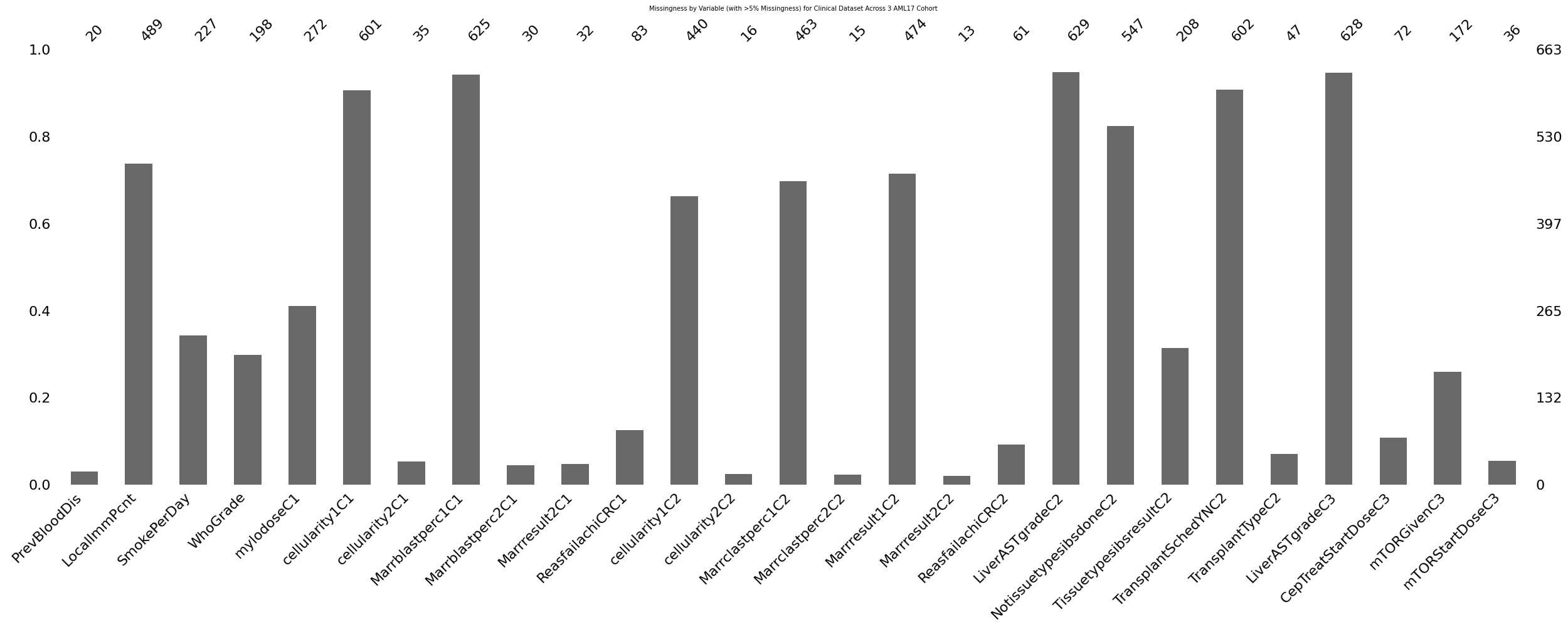

Supplement: Multimedia Appendix 10 [file bioinform-v7-e75678-s010.zip › missingness_visualisation/C3/c3_missingness_Clinical_bar.png]

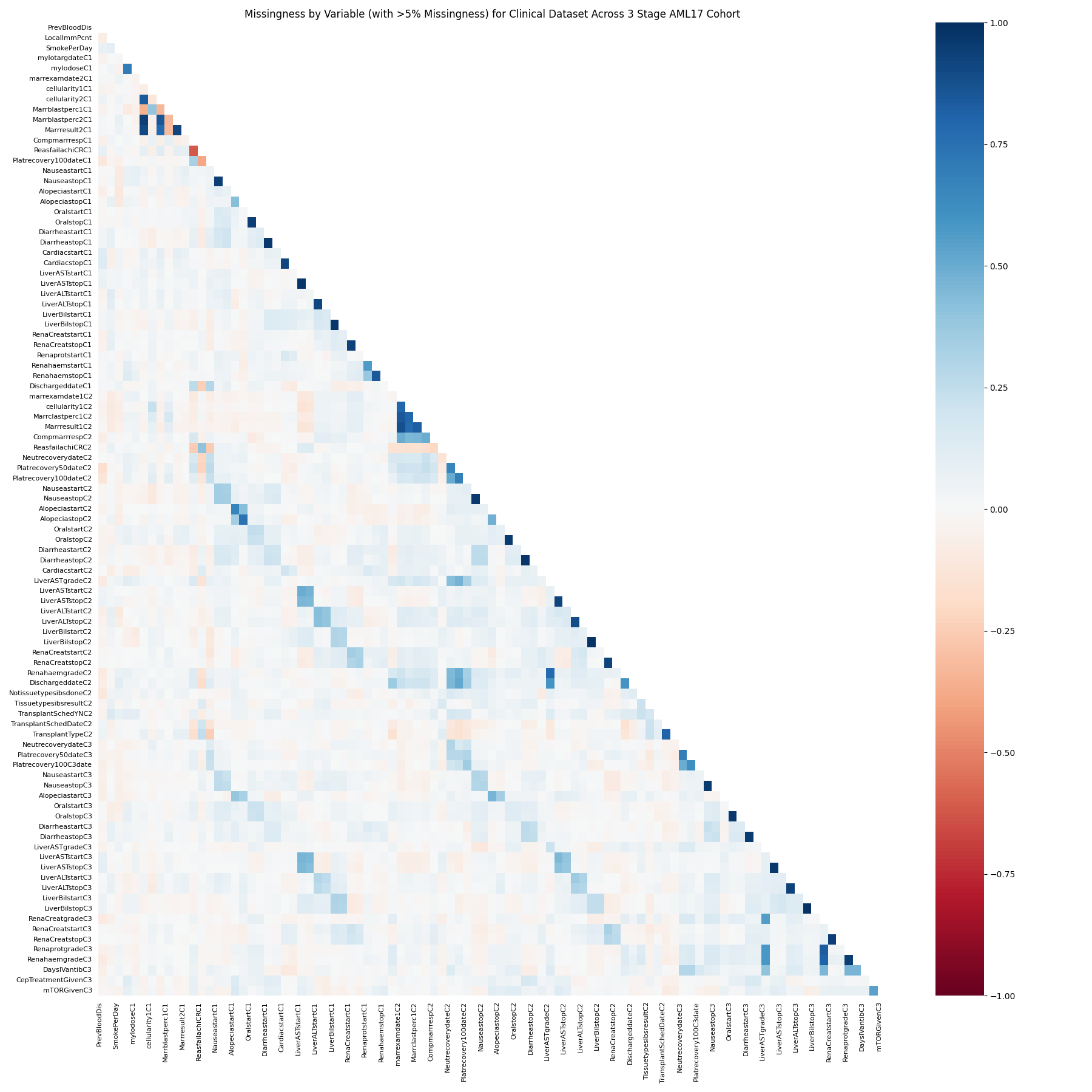

Supplement: Multimedia Appendix 10 [file bioinform-v7-e75678-s010.zip › missingness_visualisation/C3/c3_missingness_Clinical_heatmap.png]

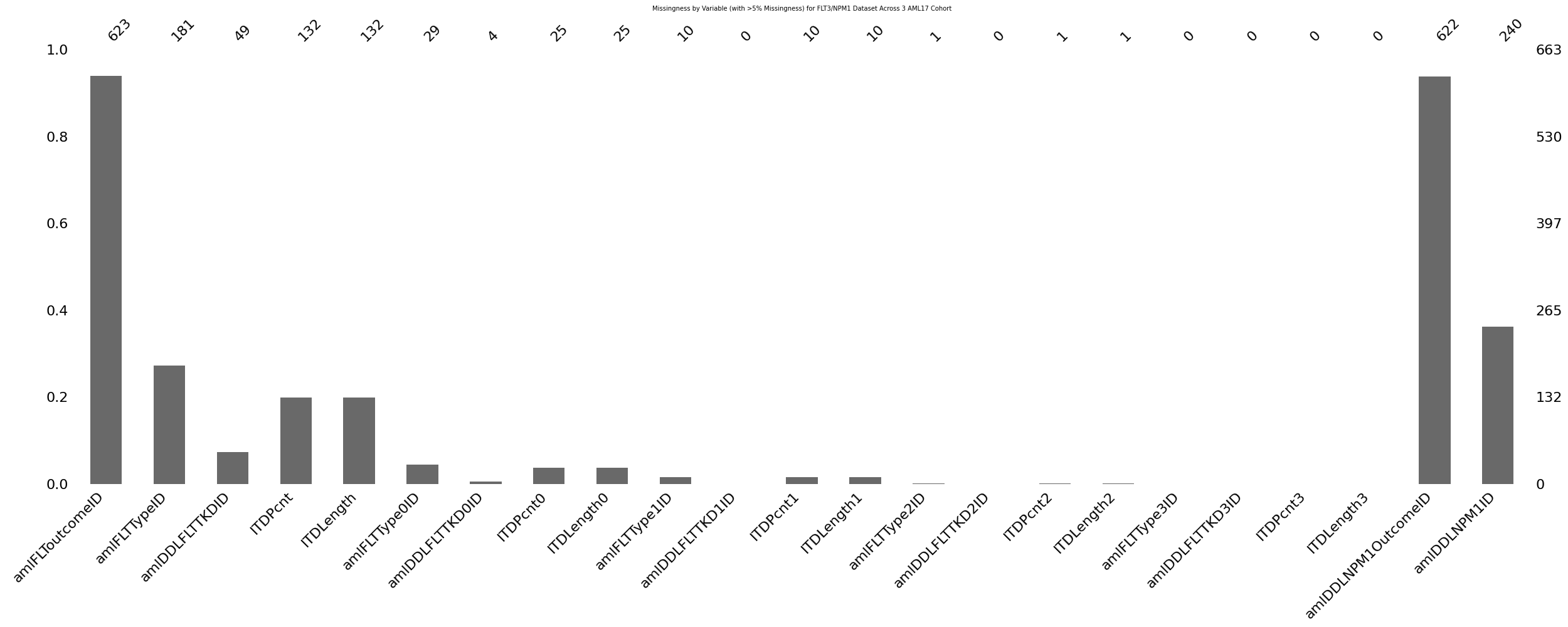

Supplement: Multimedia Appendix 10 [file bioinform-v7-e75678-s010.zip › missingness_visualisation/C3/c3_missingness_FLT3_NPM1_bar.png]

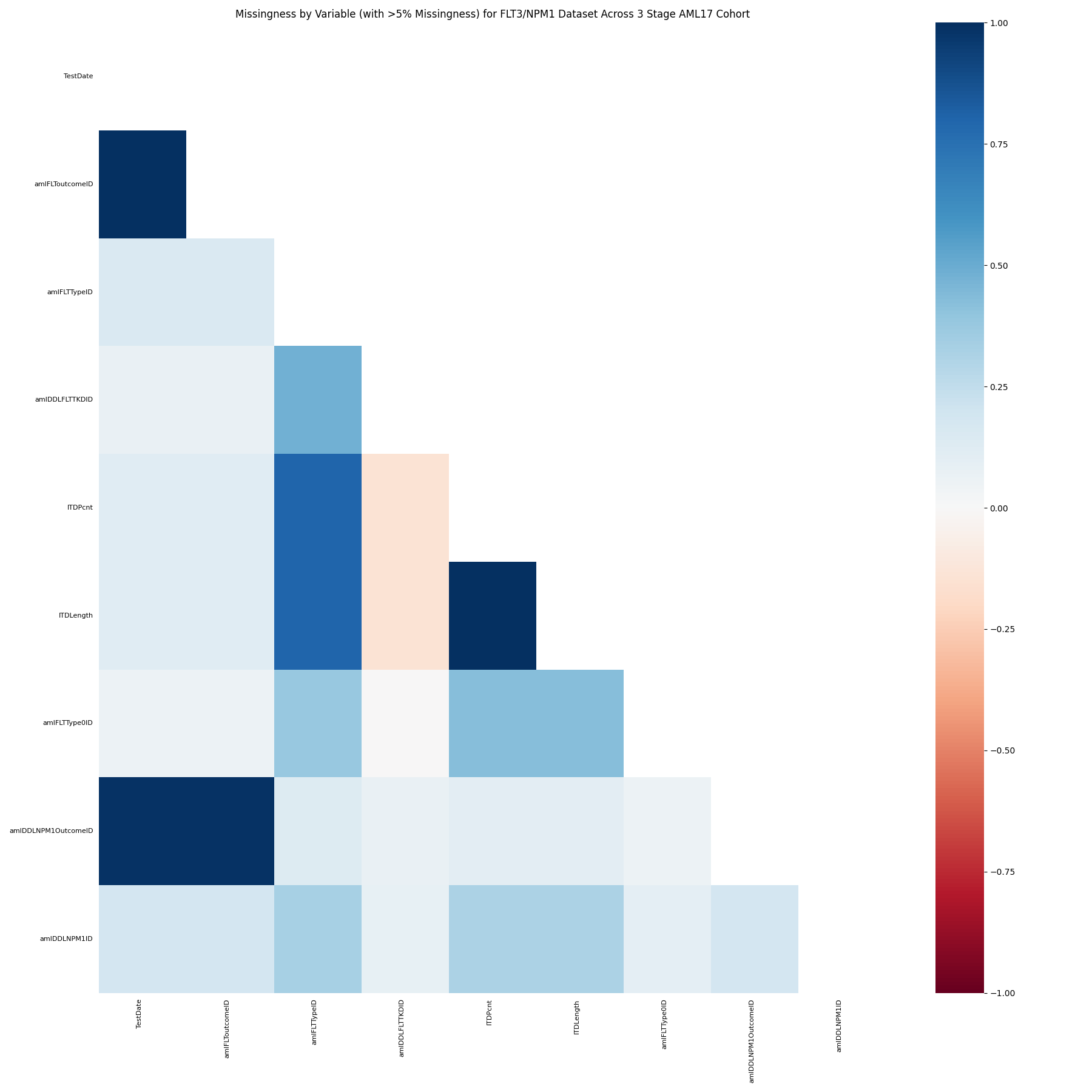

Supplement: Multimedia Appendix 10 [file bioinform-v7-e75678-s010.zip › missingness_visualisation/C3/c3_missingness_FLT3_NPM1_heatmap.png]

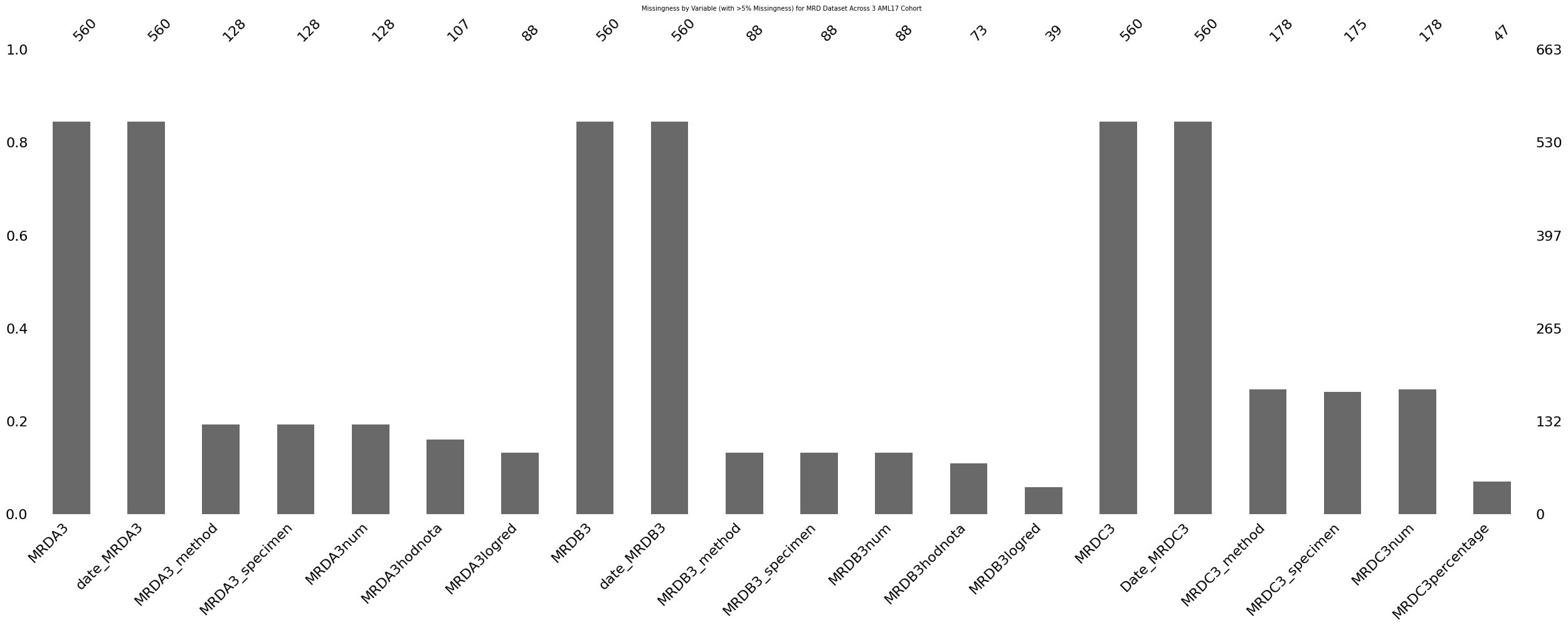

Supplement: Multimedia Appendix 10 [file bioinform-v7-e75678-s010.zip › missingness_visualisation/C3/c3_missingness_MRD_bar.png]

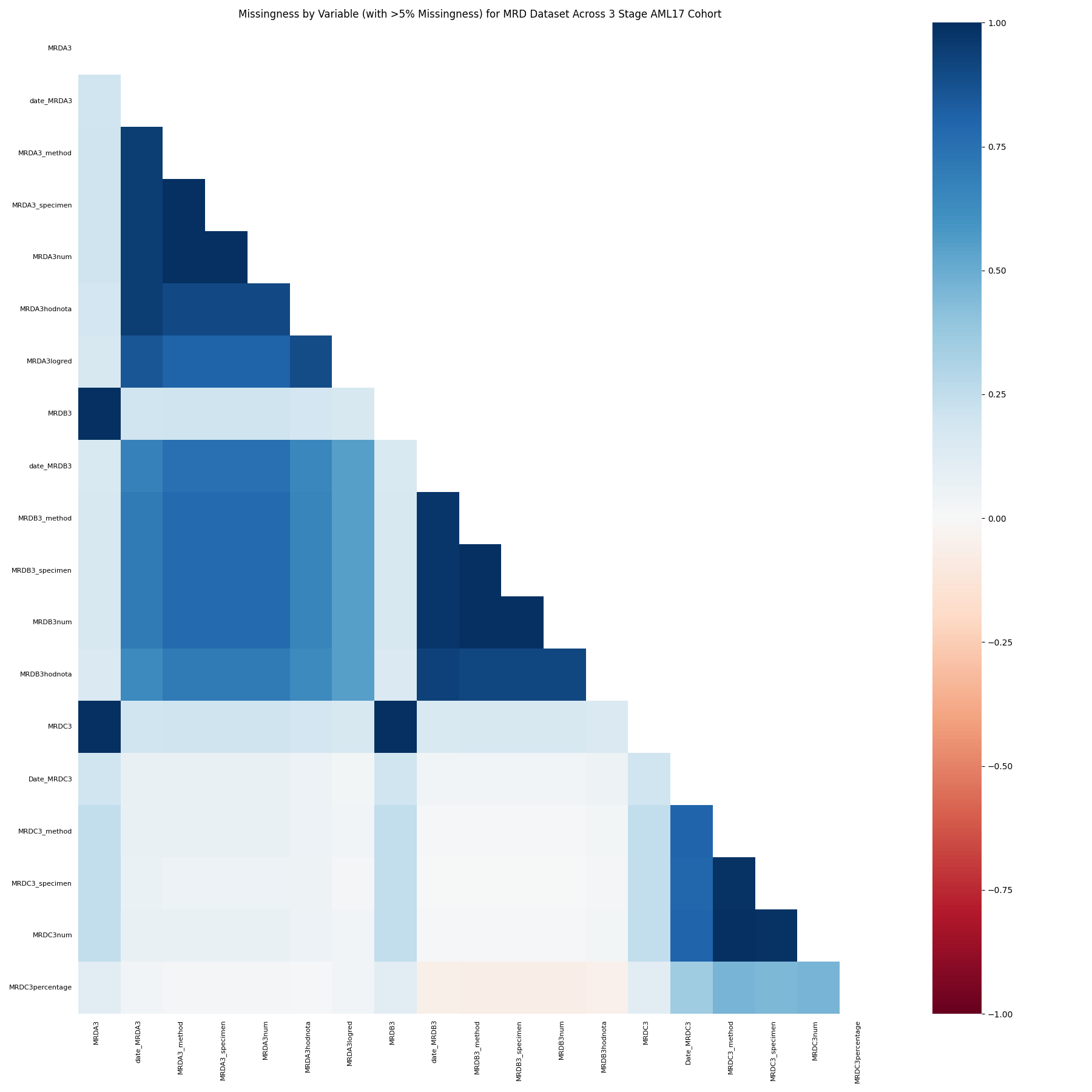

Supplement: Multimedia Appendix 10 [file bioinform-v7-e75678-s010.zip › missingness_visualisation/C3/c3_missingness_MRD_heatmap.png]

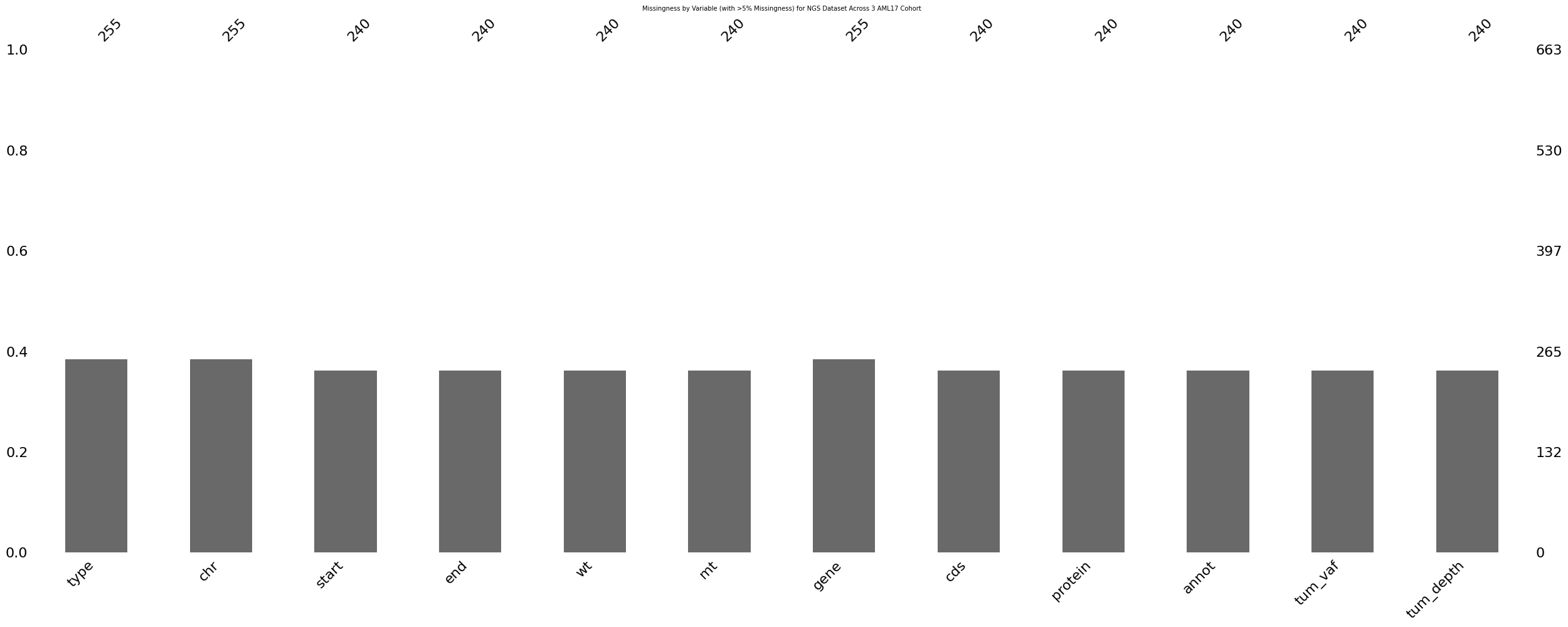

Supplement: Multimedia Appendix 10 [file bioinform-v7-e75678-s010.zip › missingness_visualisation/C3/c3_missingness_NGS_bar.png]

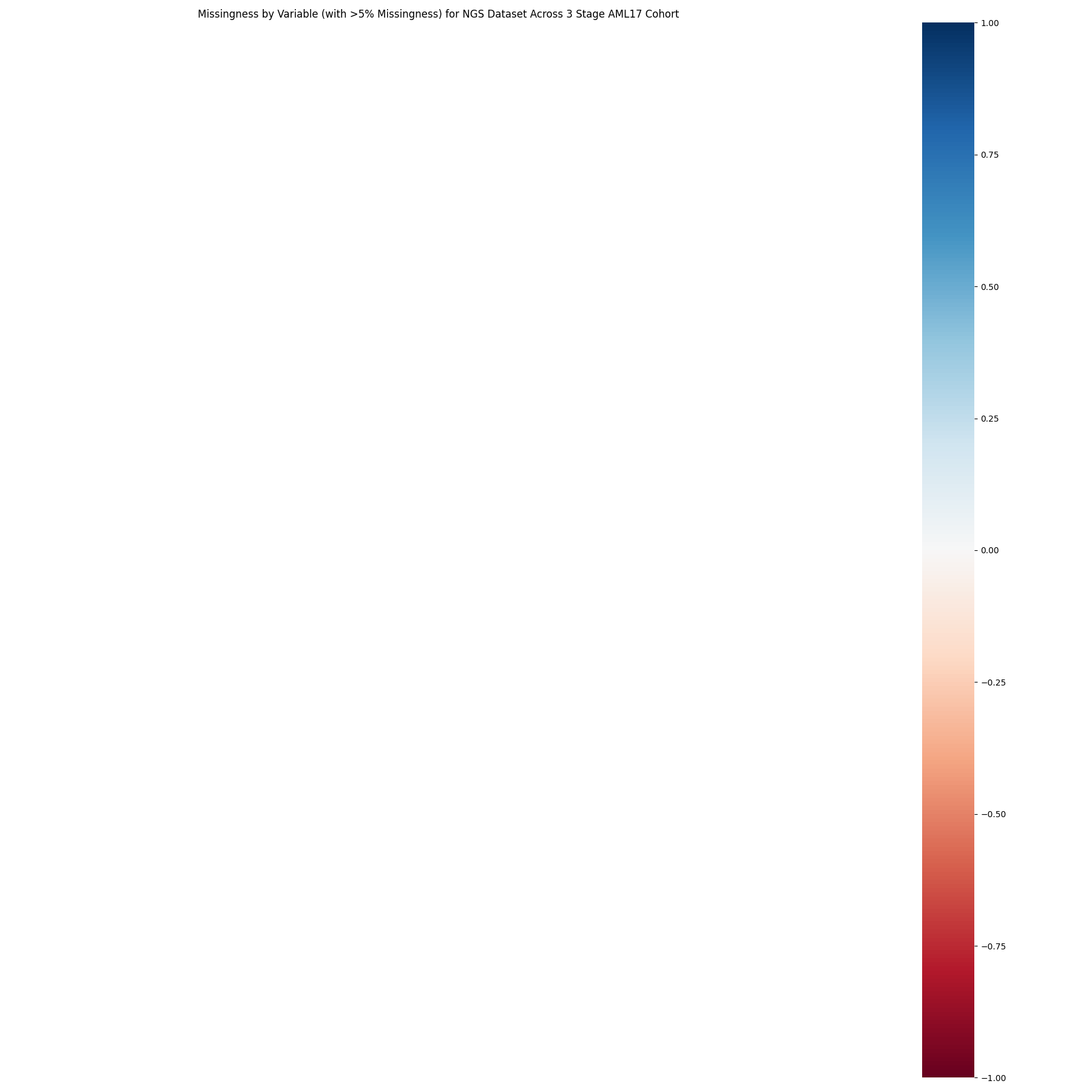

Supplement: Multimedia Appendix 10 [file bioinform-v7-e75678-s010.zip › missingness_visualisation/C3/c3_missingness_NGS_heatmap.png]

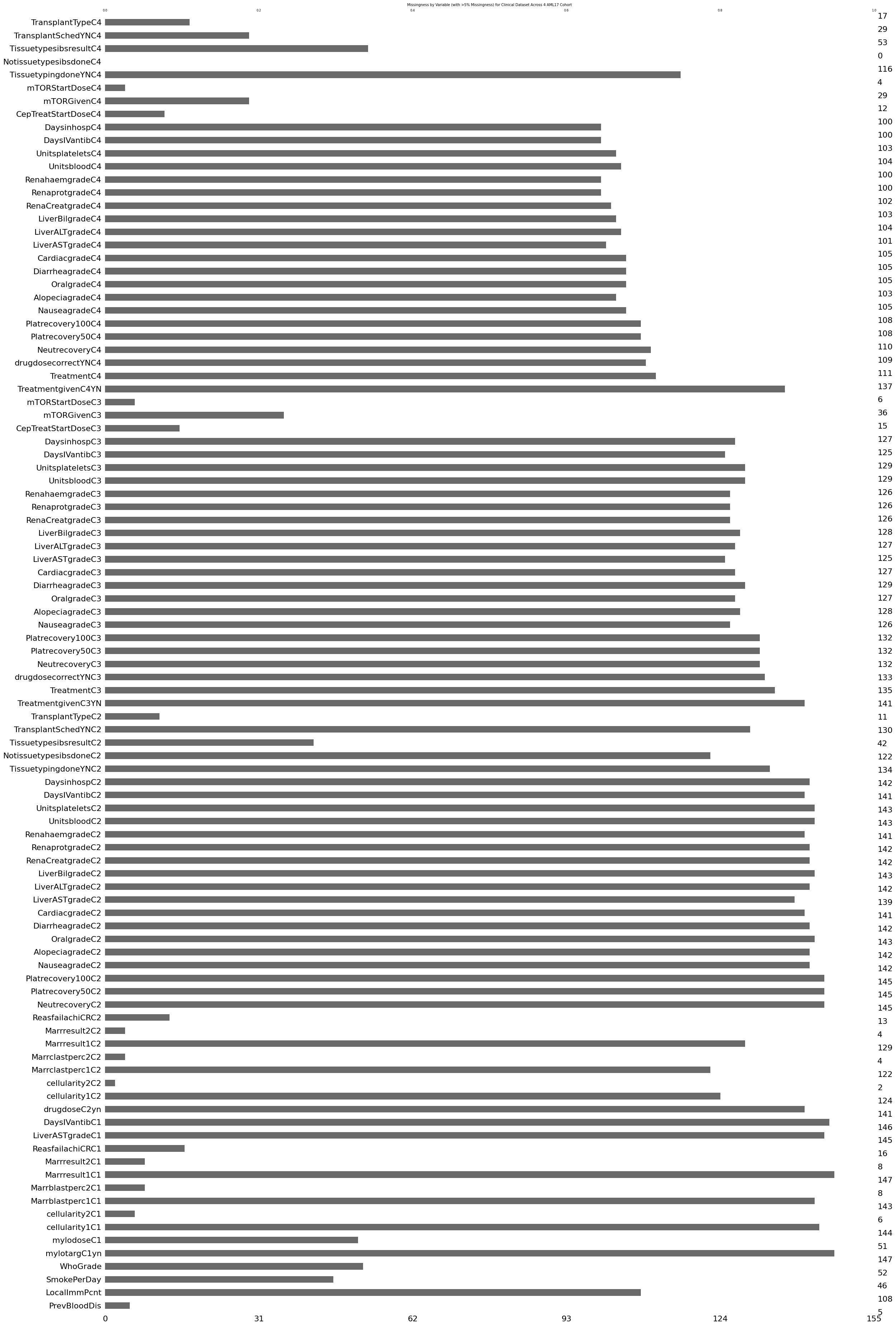

Supplement: Multimedia Appendix 10 [file bioinform-v7-e75678-s010.zip › missingness_visualisation/C4/c4_missingness_Clinical_bar.png]

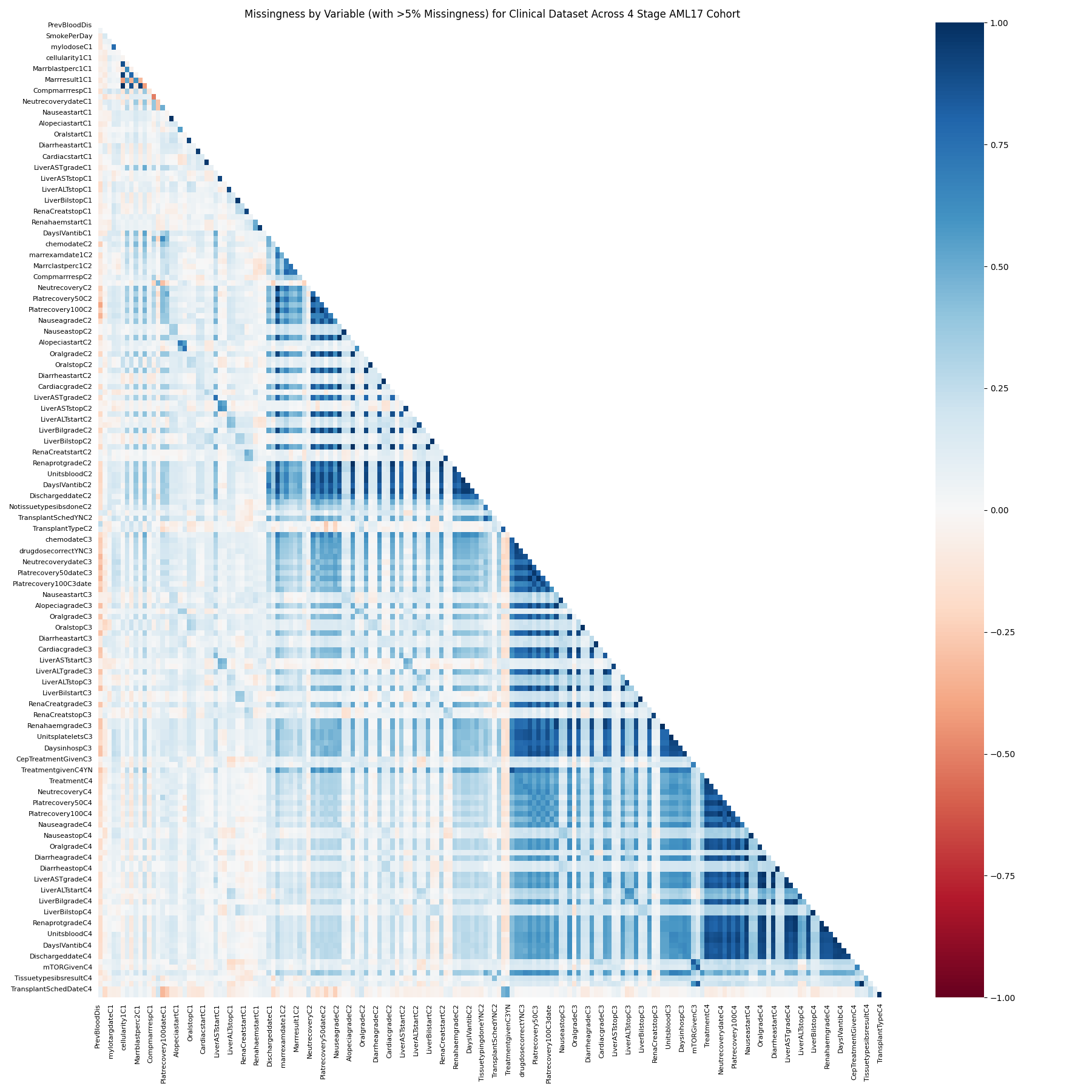

Supplement: Multimedia Appendix 10 [file bioinform-v7-e75678-s010.zip › missingness_visualisation/C4/c4_missingness_Clinical_heatmap.png]

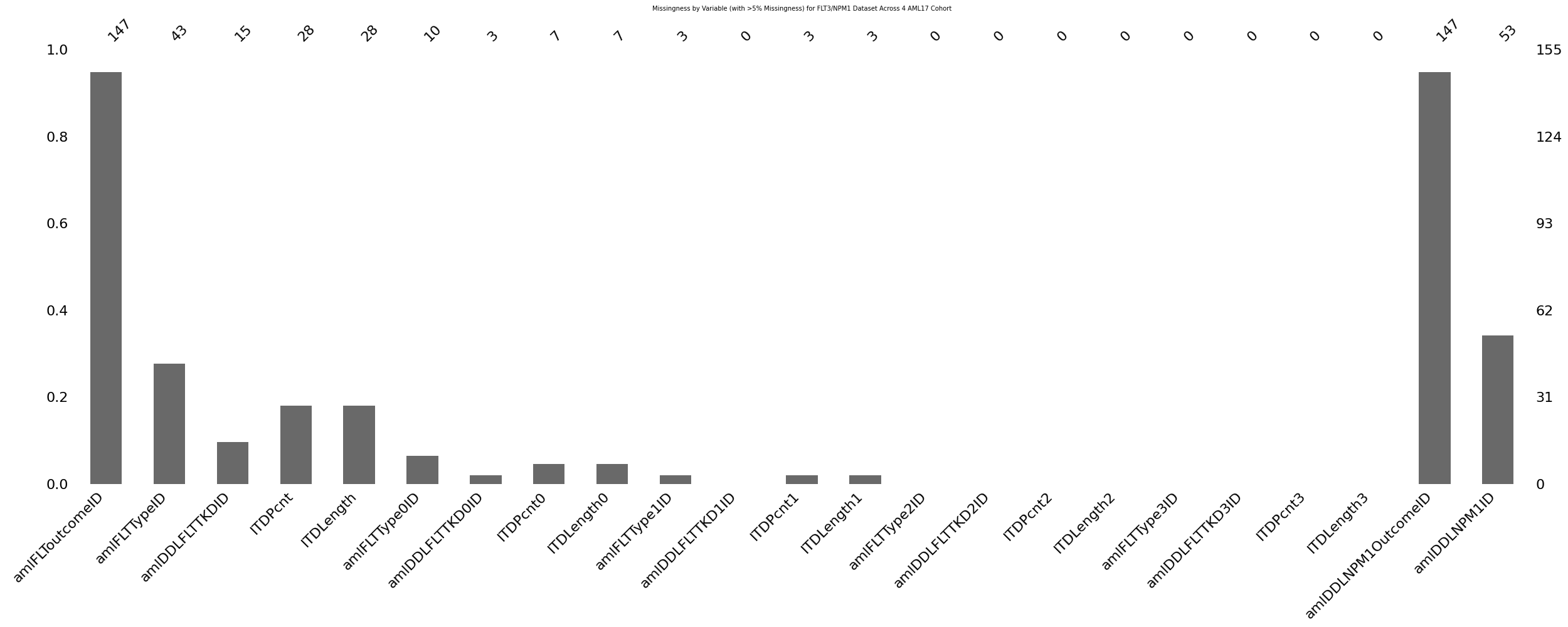

Supplement: Multimedia Appendix 10 [file bioinform-v7-e75678-s010.zip › missingness_visualisation/C4/c4_missingness_FLT3_NPM1_bar.png]

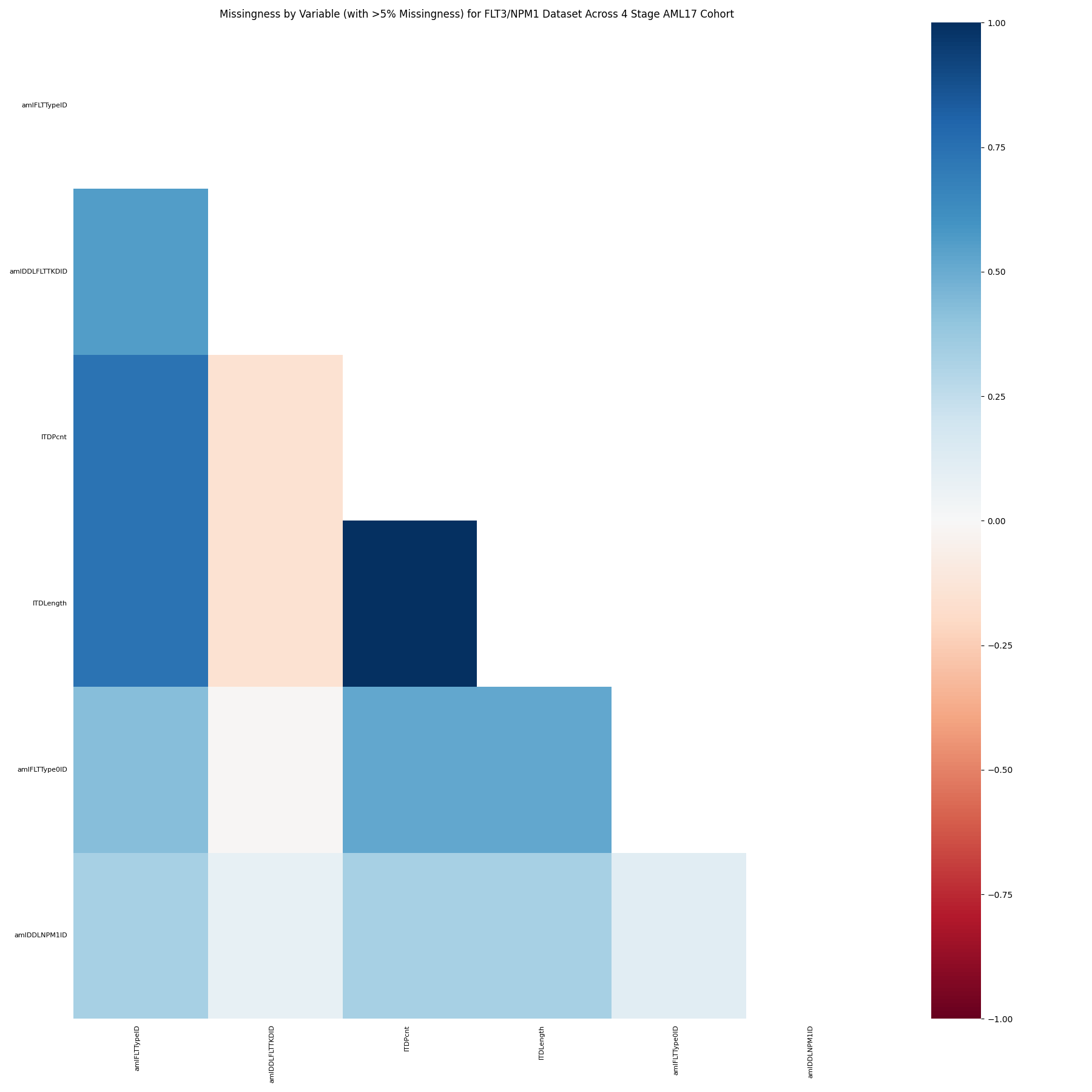

Supplement: Multimedia Appendix 10 [file bioinform-v7-e75678-s010.zip › missingness_visualisation/C4/c4_missingness_FLT3_NPM1_heatmap.png]

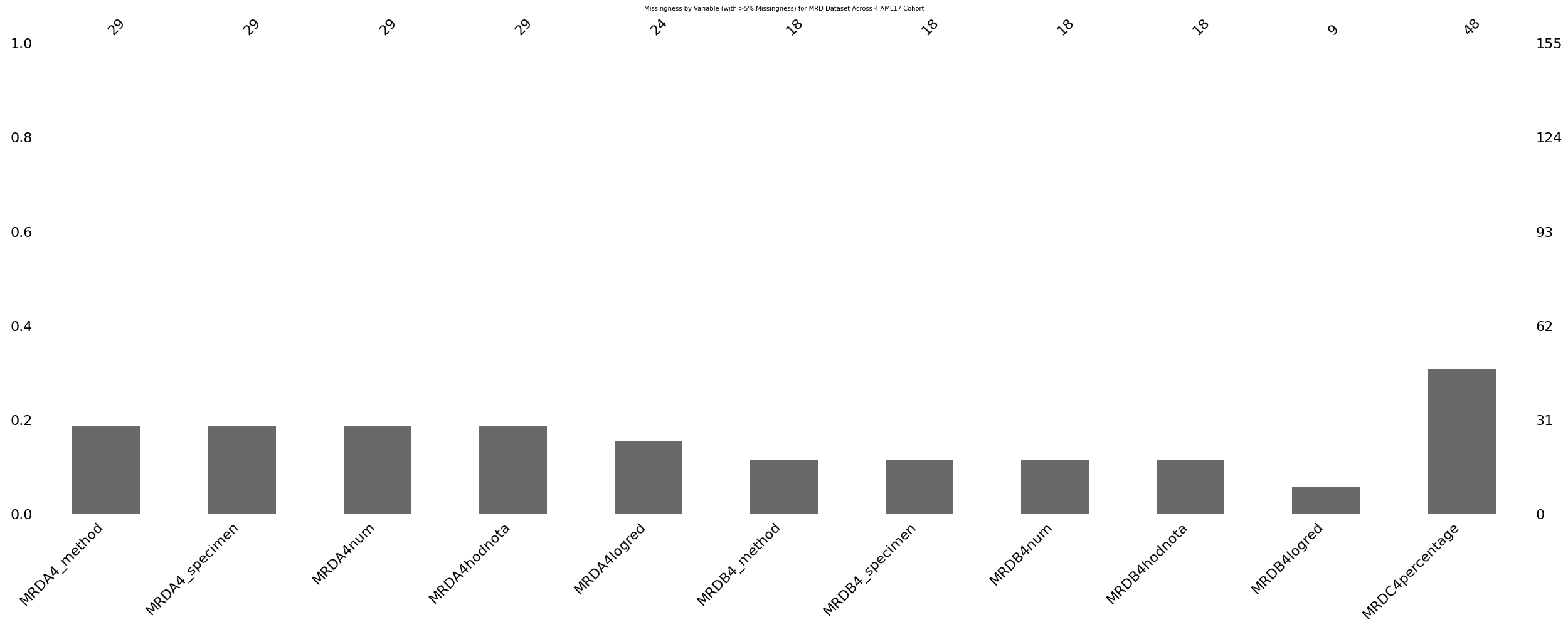

Supplement: Multimedia Appendix 10 [file bioinform-v7-e75678-s010.zip › missingness_visualisation/C4/c4_missingness_MRD_bar.png]

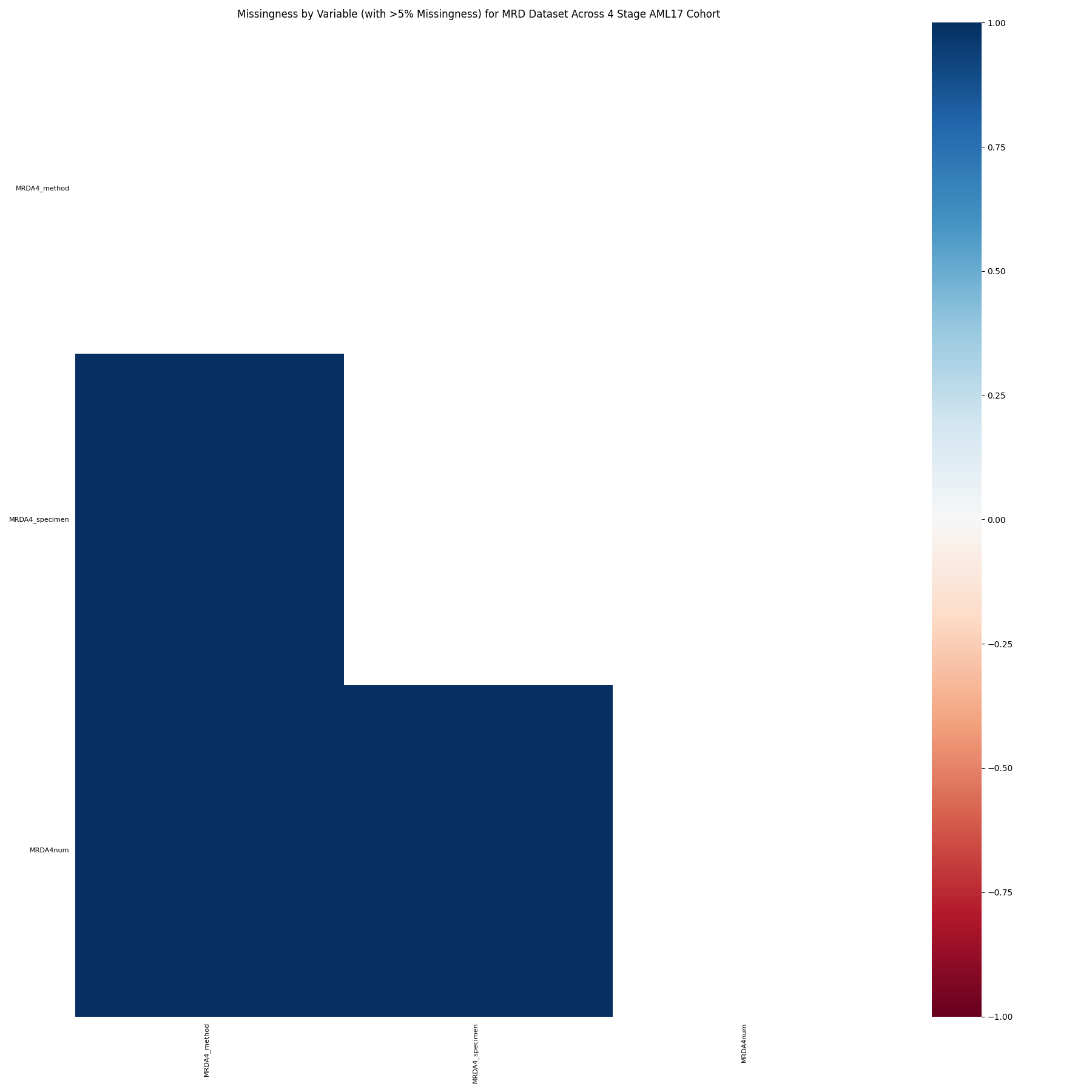

Supplement: Multimedia Appendix 10 [file bioinform-v7-e75678-s010.zip › missingness_visualisation/C4/c4_missingness_MRD_heatmap.png]

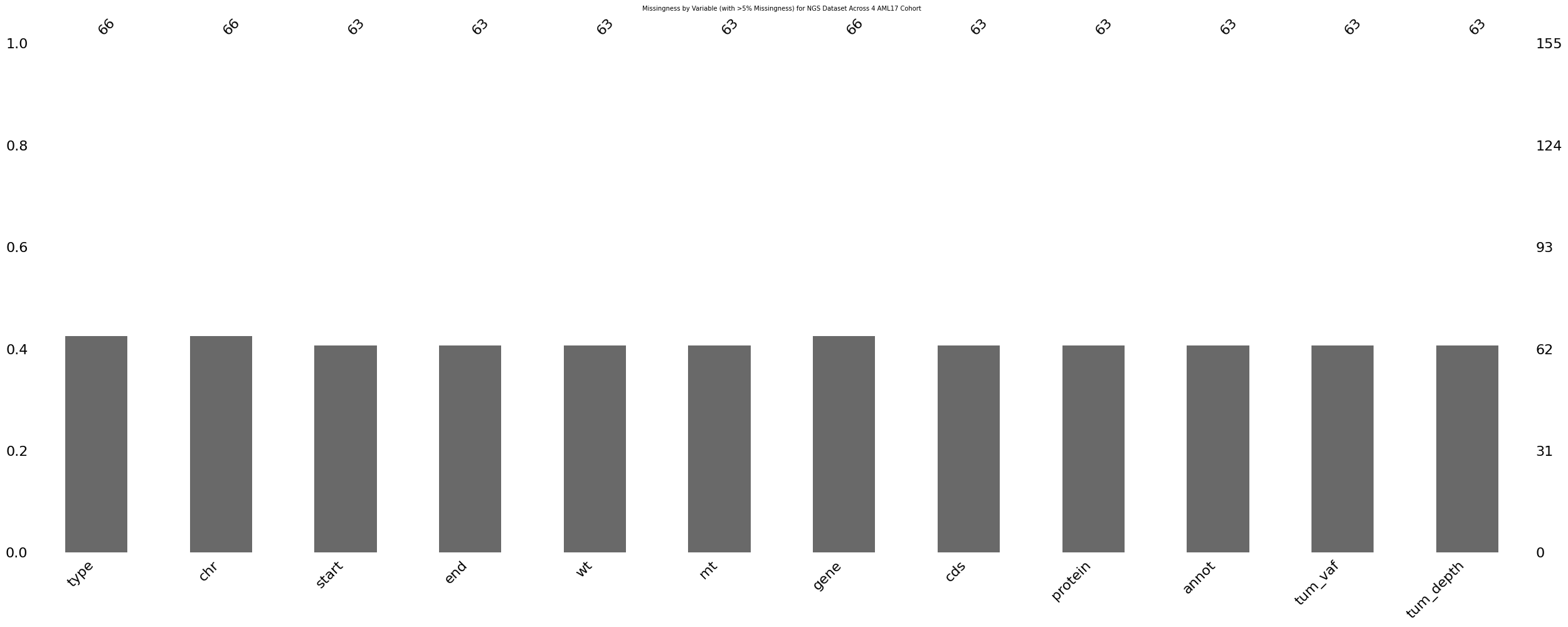

Supplement: Multimedia Appendix 10 [file bioinform-v7-e75678-s010.zip › missingness_visualisation/C4/c4_missingness_NGS_bar.png]

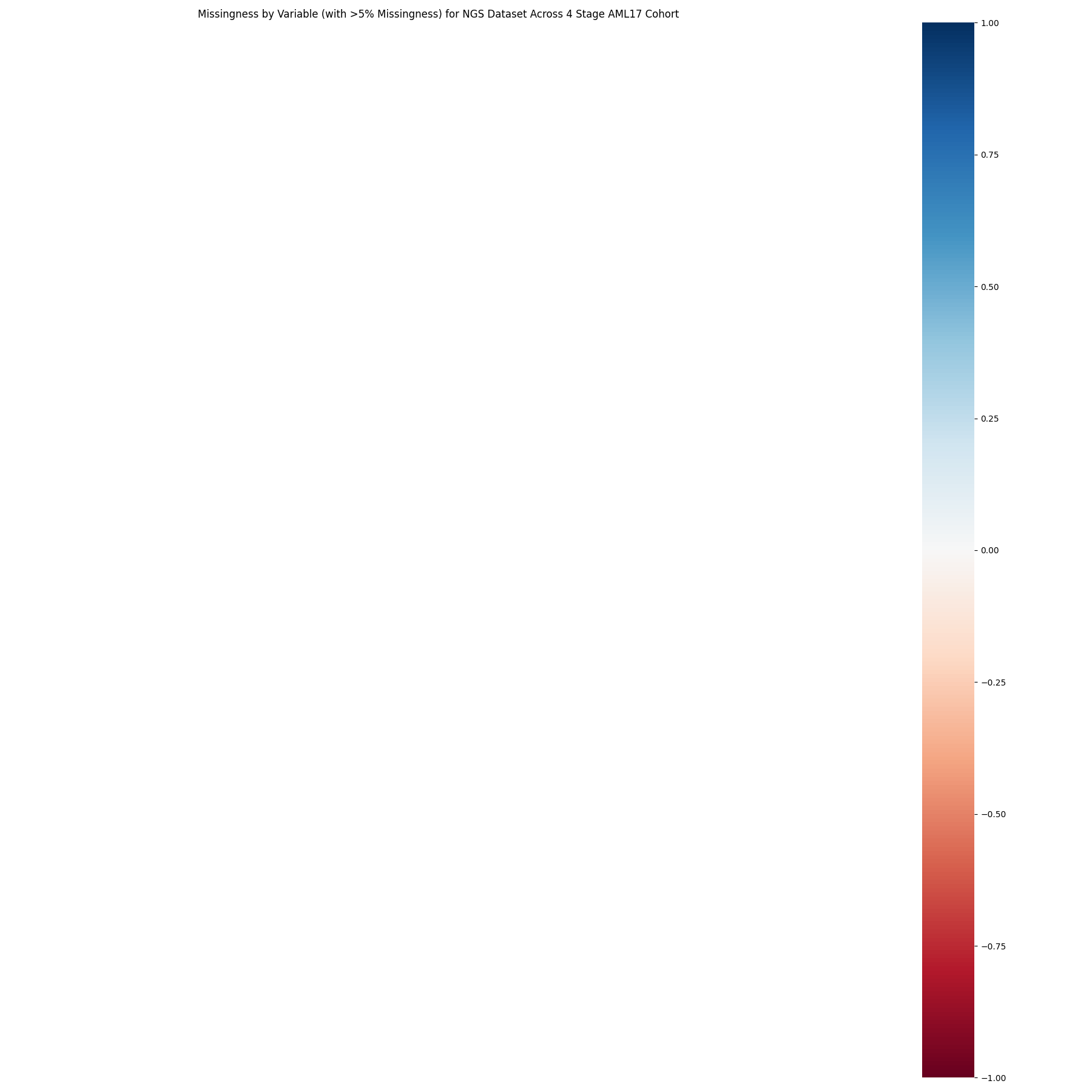

Supplement: Multimedia Appendix 10 [file bioinform-v7-e75678-s010.zip › missingness_visualisation/C4/c4_missingness_NGS_heatmap.png]

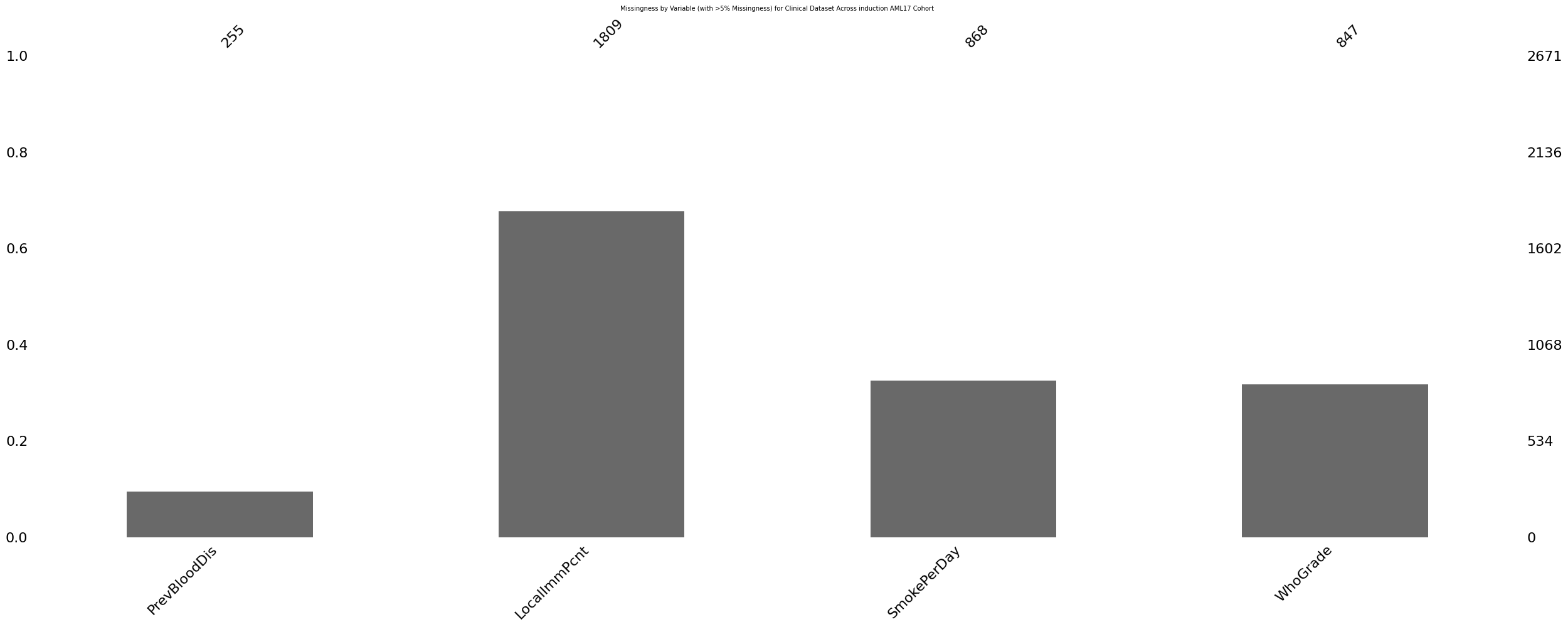

Supplement: Multimedia Appendix 10 [file bioinform-v7-e75678-s010.zip › missingness_visualisation/induction/induction_Clinical_bar.png]

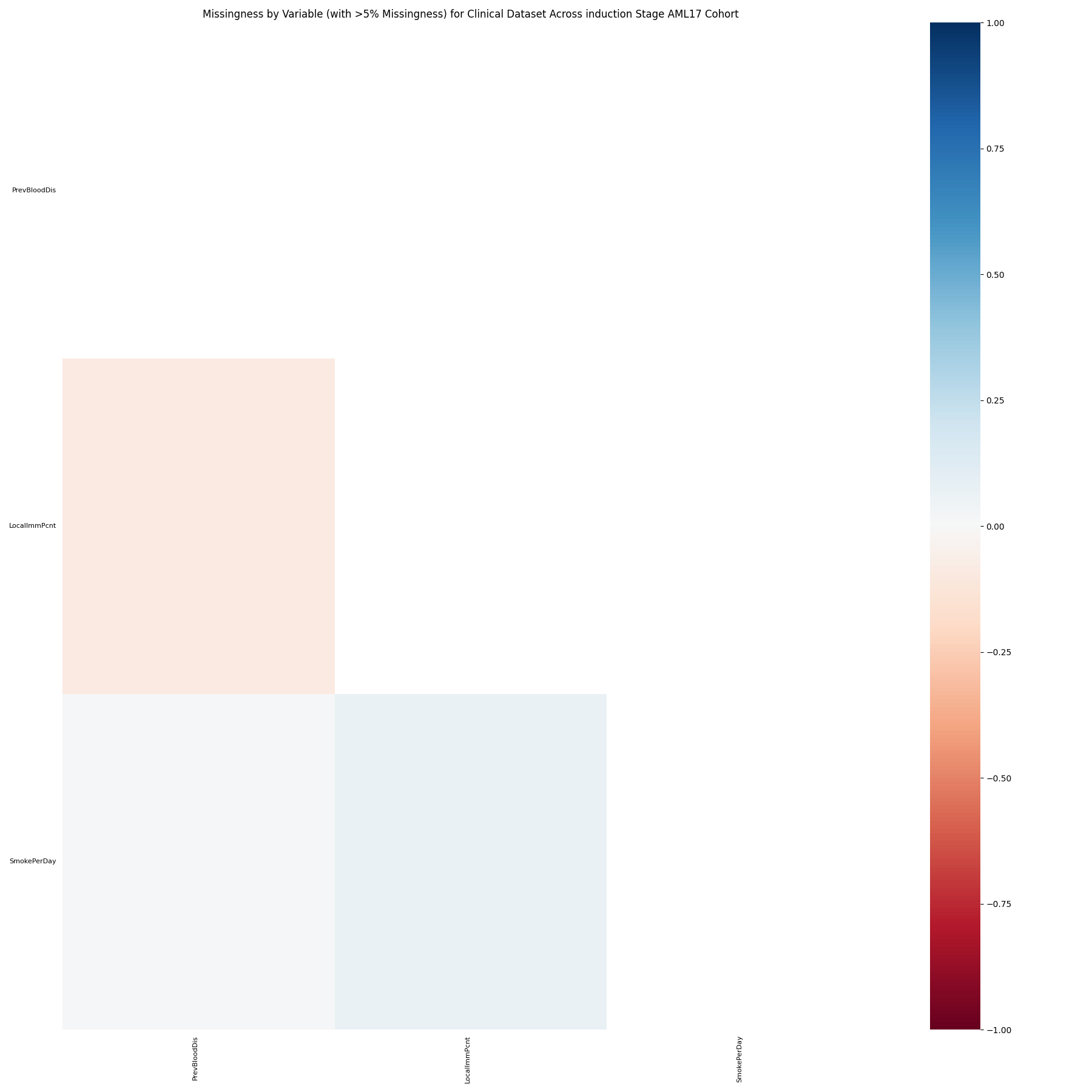

Supplement: Multimedia Appendix 10 [file bioinform-v7-e75678-s010.zip › missingness_visualisation/induction/induction_Clinical_heatmap.png]

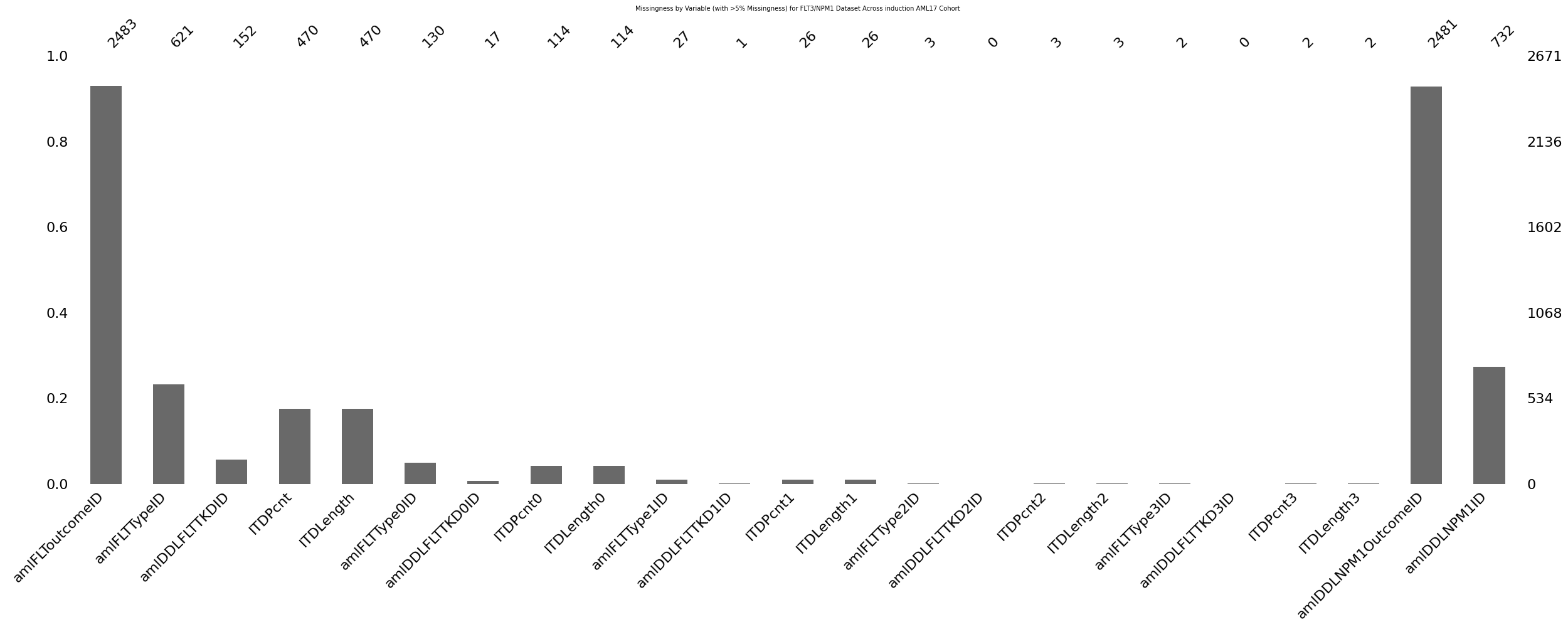

Supplement: Multimedia Appendix 10 [file bioinform-v7-e75678-s010.zip › missingness_visualisation/induction/induction_FLT3_NPM1_bar.png]

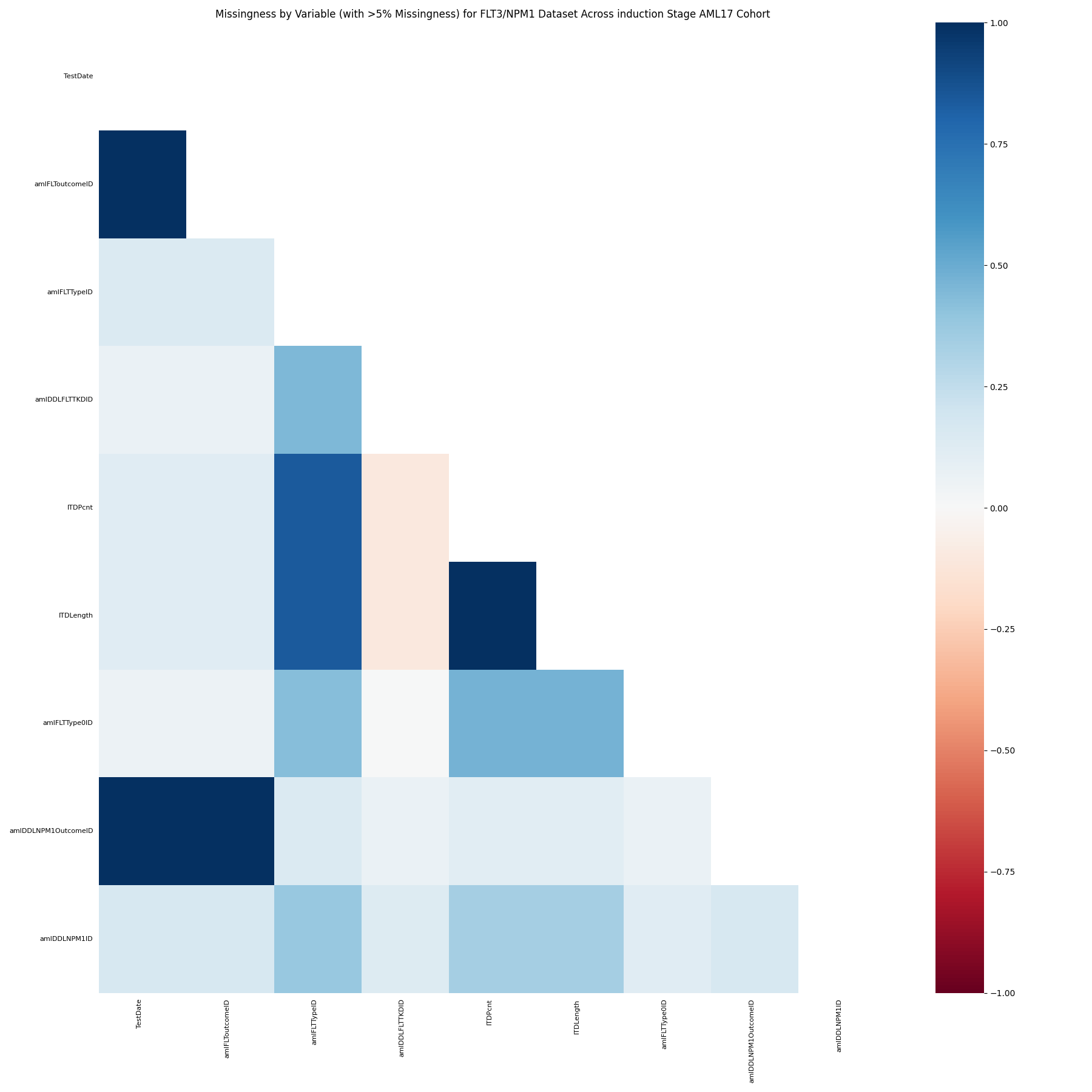

Supplement: Multimedia Appendix 10 [file bioinform-v7-e75678-s010.zip › missingness_visualisation/induction/induction_FLT3_NPM1_heatmap.png]

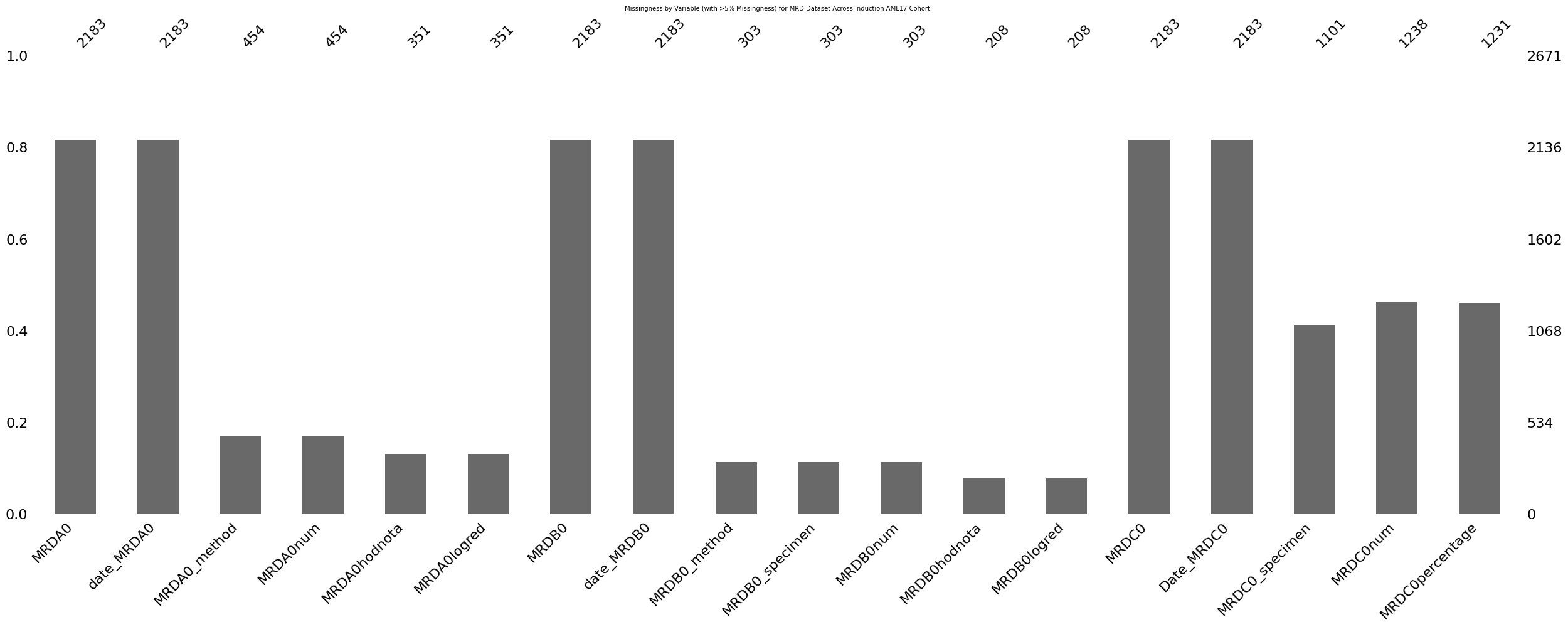

Supplement: Multimedia Appendix 10 [file bioinform-v7-e75678-s010.zip › missingness_visualisation/induction/induction_MRD_bar.png]

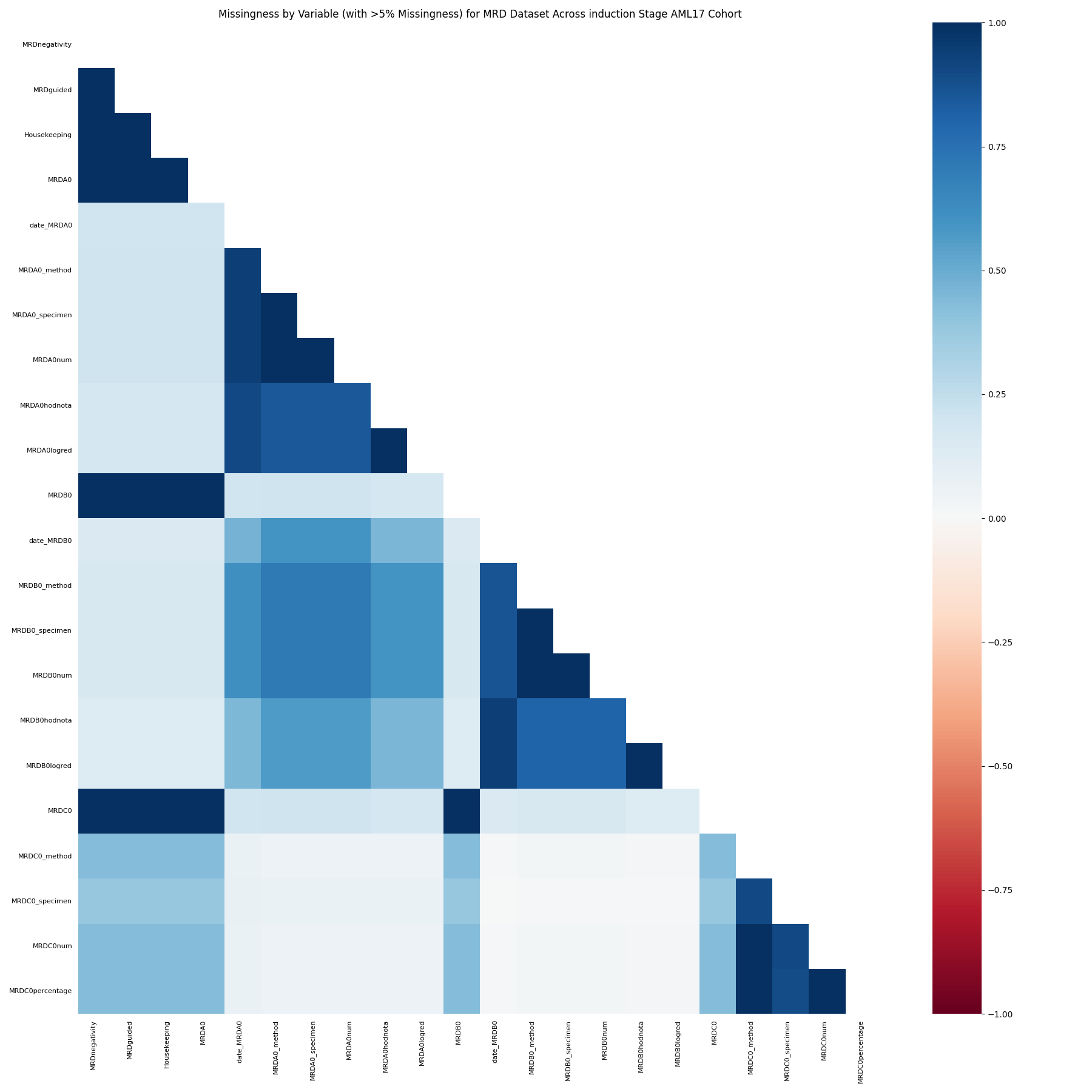

Supplement: Multimedia Appendix 10 [file bioinform-v7-e75678-s010.zip › missingness_visualisation/induction/induction_MRD_heatmap.png]

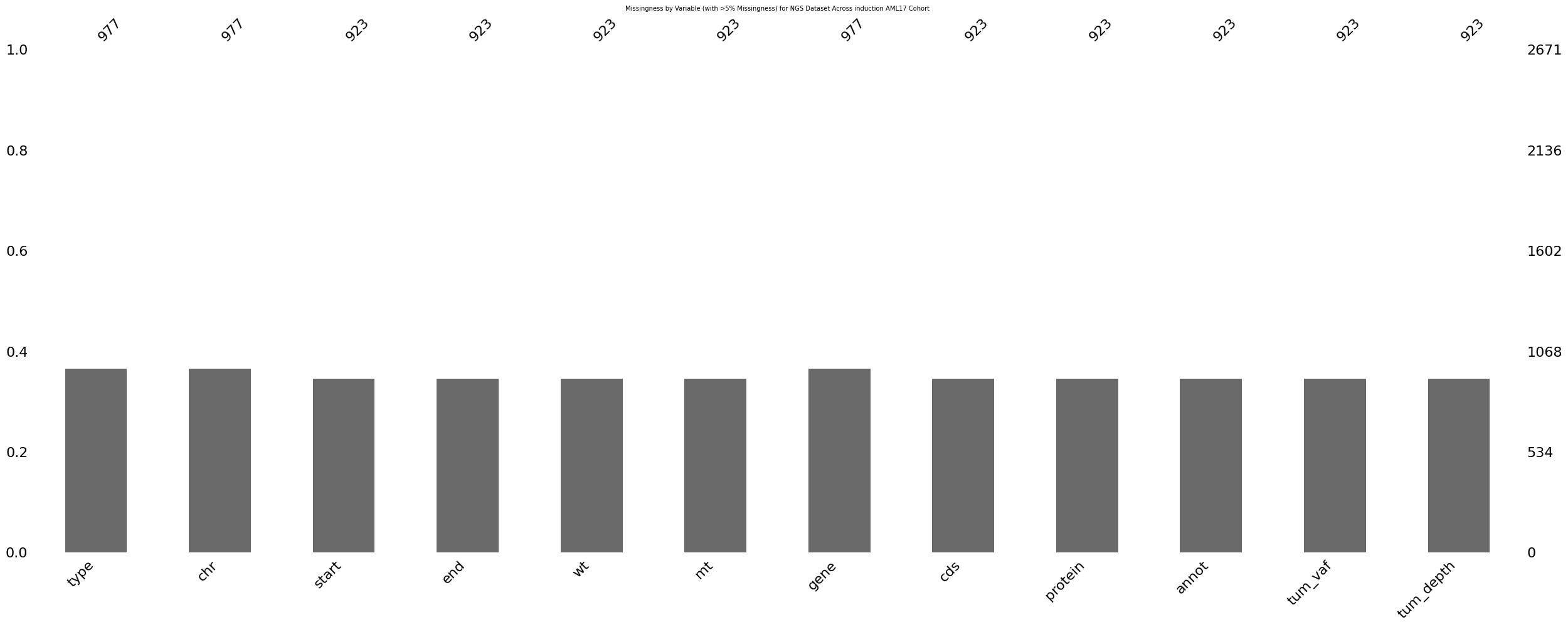

Supplement: Multimedia Appendix 10 [file bioinform-v7-e75678-s010.zip › missingness_visualisation/induction/induction_NGS_bar.png]

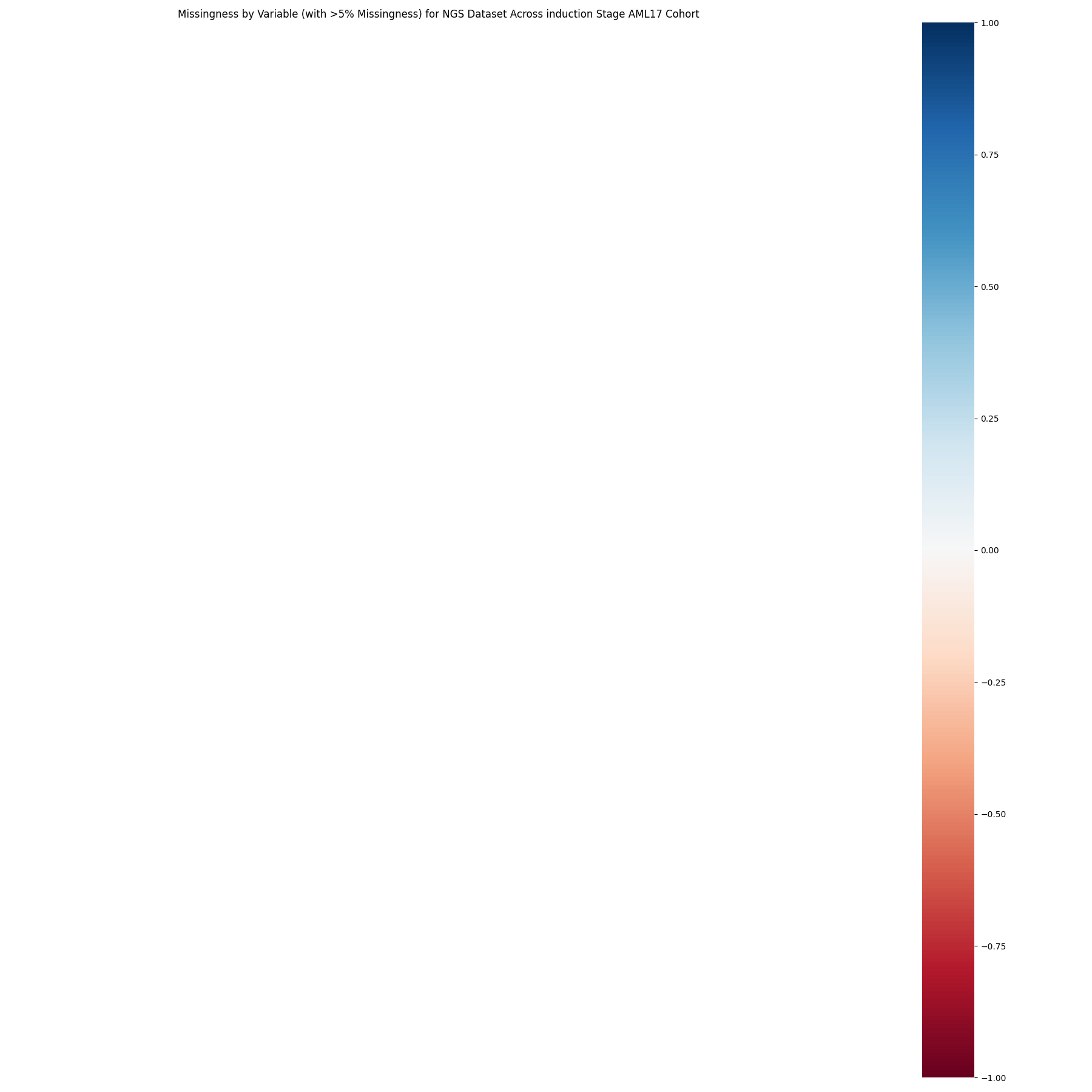

Supplement: Multimedia Appendix 10 [file bioinform-v7-e75678-s010.zip › missingness_visualisation/induction/NGS_heatmap.png]

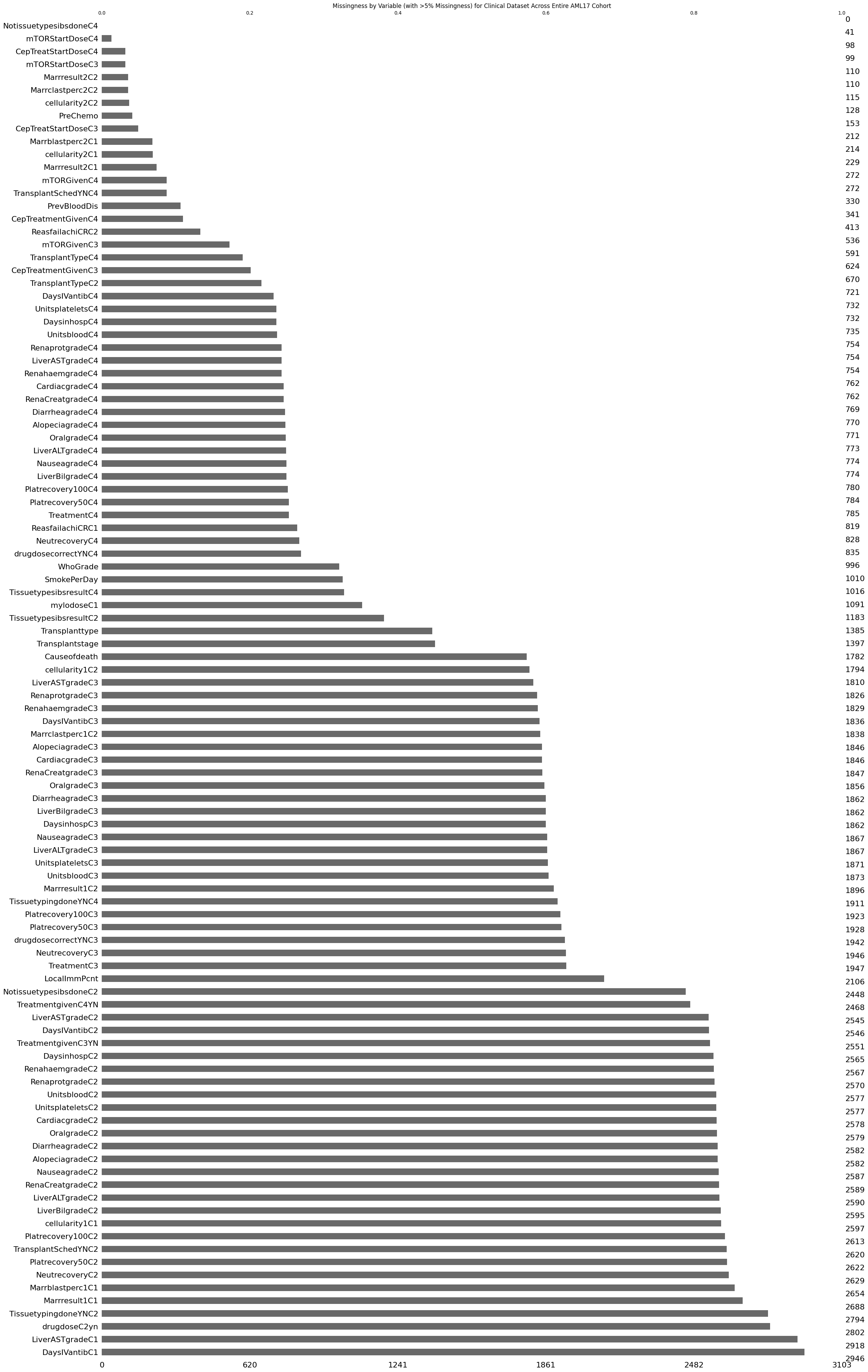

Supplement: Multimedia Appendix 10 [file bioinform-v7-e75678-s010.zip › missingness_visualisation/whole_cohort/Clinical_bar.png]

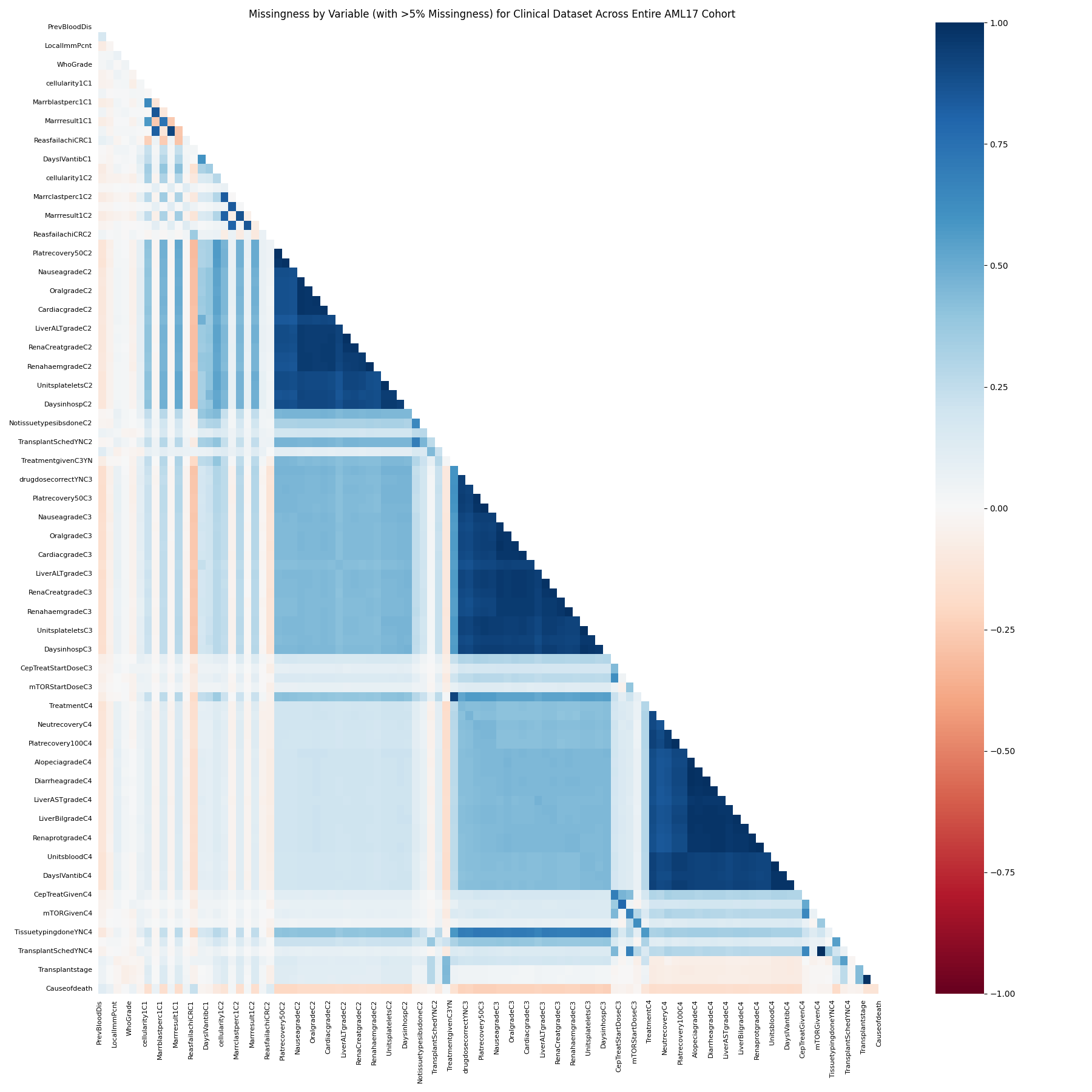

Supplement: Multimedia Appendix 10 [file bioinform-v7-e75678-s010.zip › missingness_visualisation/whole_cohort/Clinical_heatmap.png]

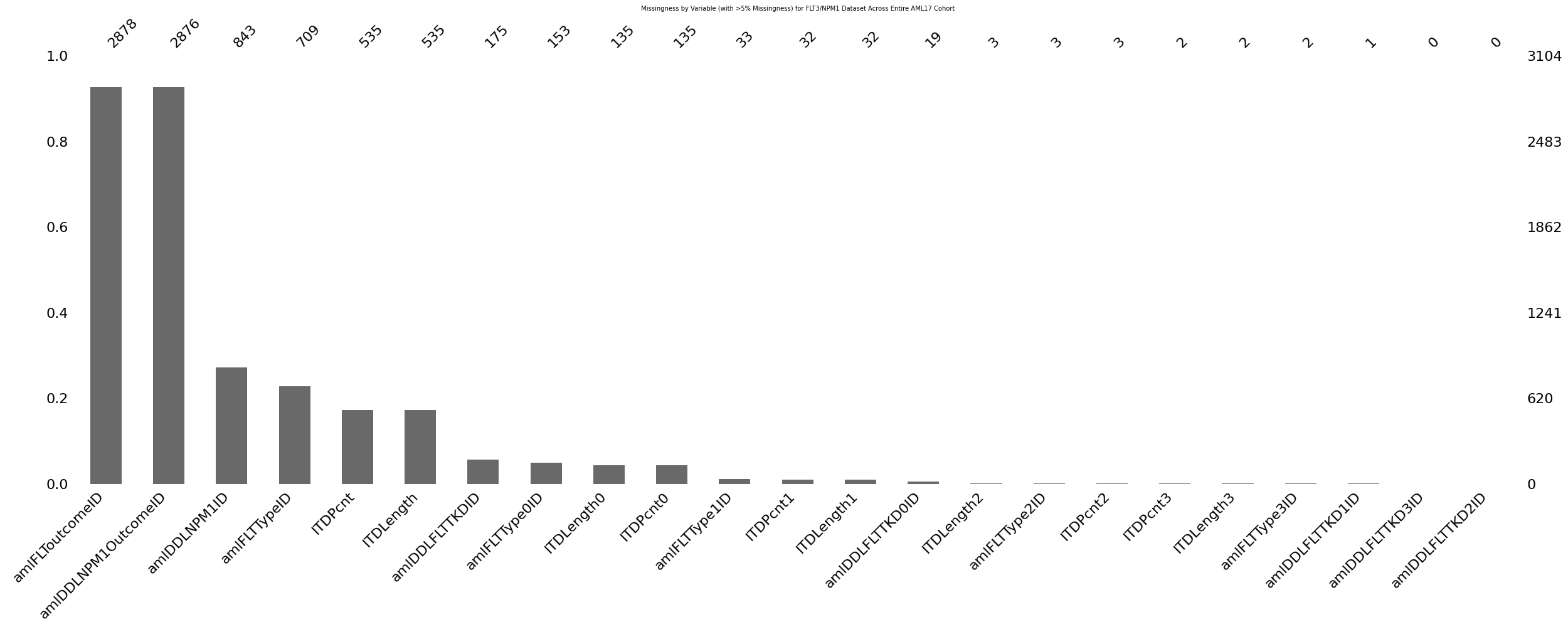

Supplement: Multimedia Appendix 10 [file bioinform-v7-e75678-s010.zip › missingness_visualisation/whole_cohort/FLT3_NPM1_bar.png]

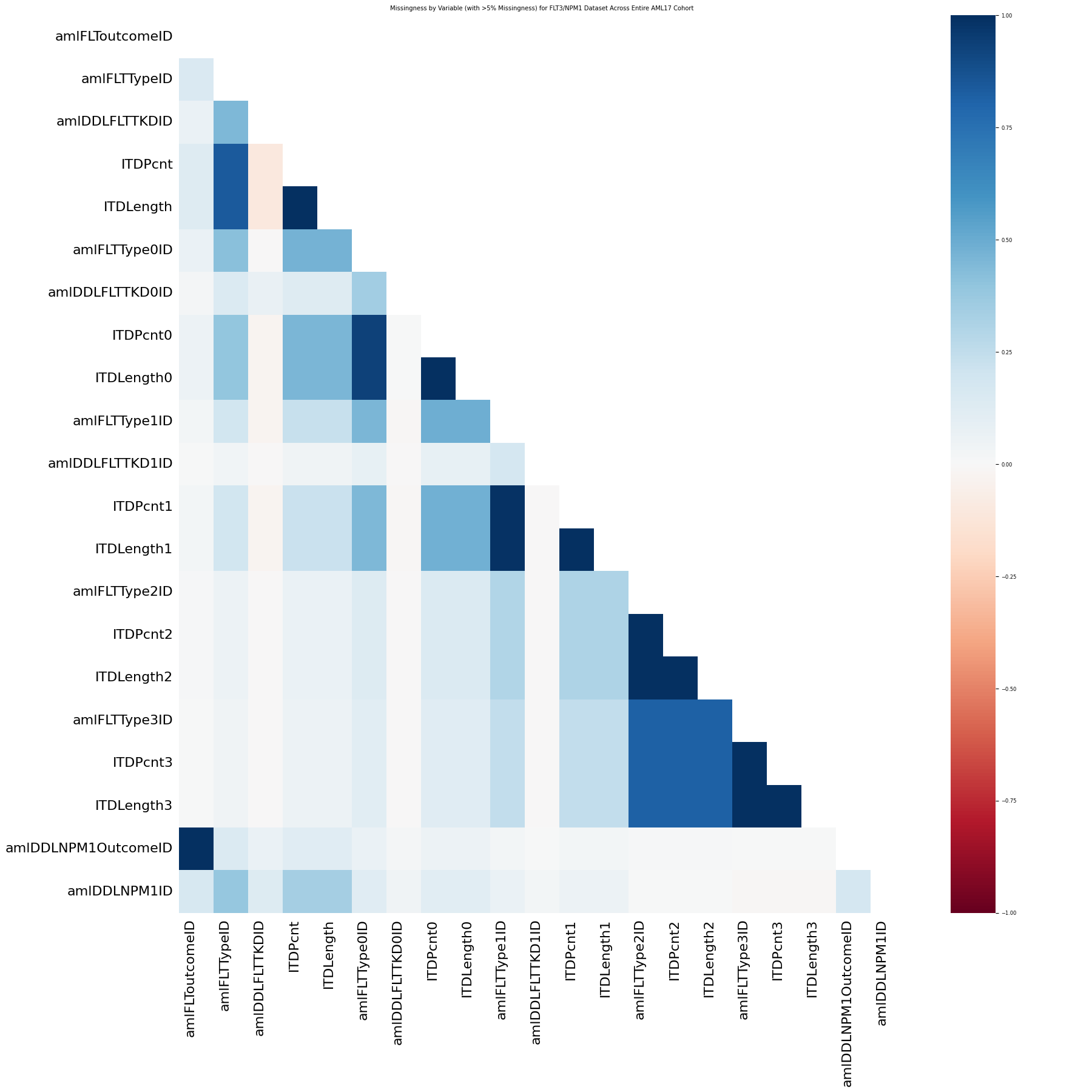

Supplement: Multimedia Appendix 10 [file bioinform-v7-e75678-s010.zip › missingness_visualisation/whole_cohort/FLT3_NPM1_heatmap.png]

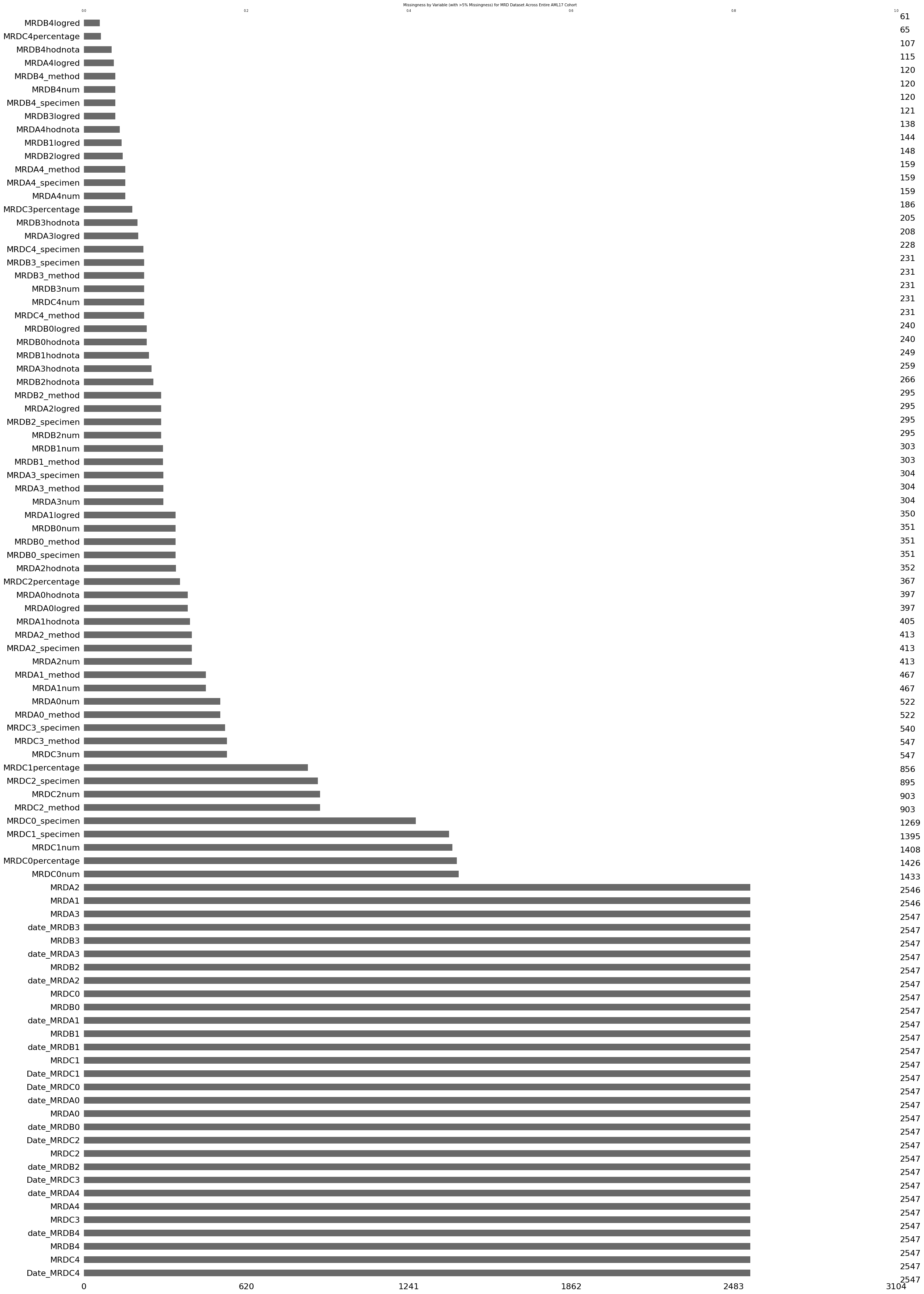

Supplement: Multimedia Appendix 10 [file bioinform-v7-e75678-s010.zip › missingness_visualisation/whole_cohort/MRD_bar.png]

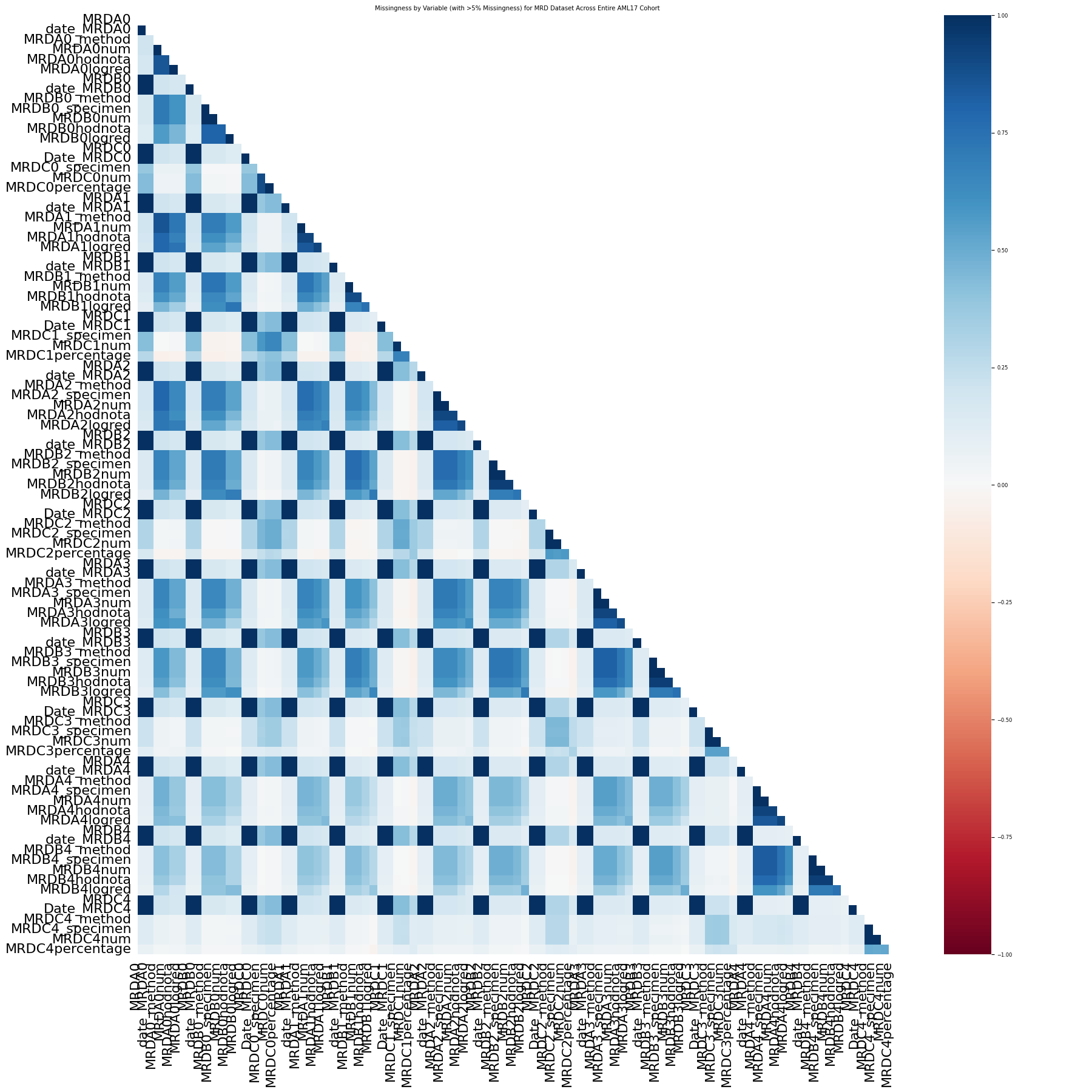

Supplement: Multimedia Appendix 10 [file bioinform-v7-e75678-s010.zip › missingness_visualisation/whole_cohort/MRD_heatmap.png]

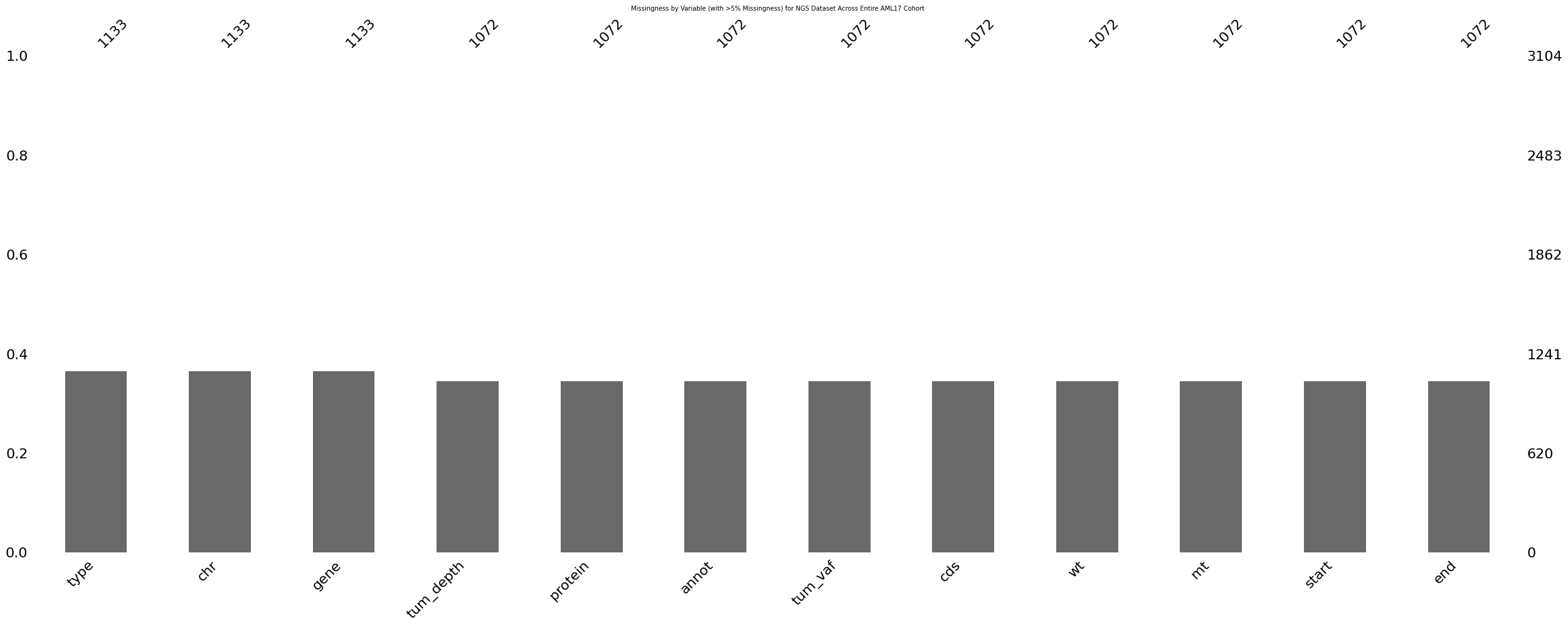

Supplement: Multimedia Appendix 10 [file bioinform-v7-e75678-s010.zip › missingness_visualisation/whole_cohort/NGS_bar.png]

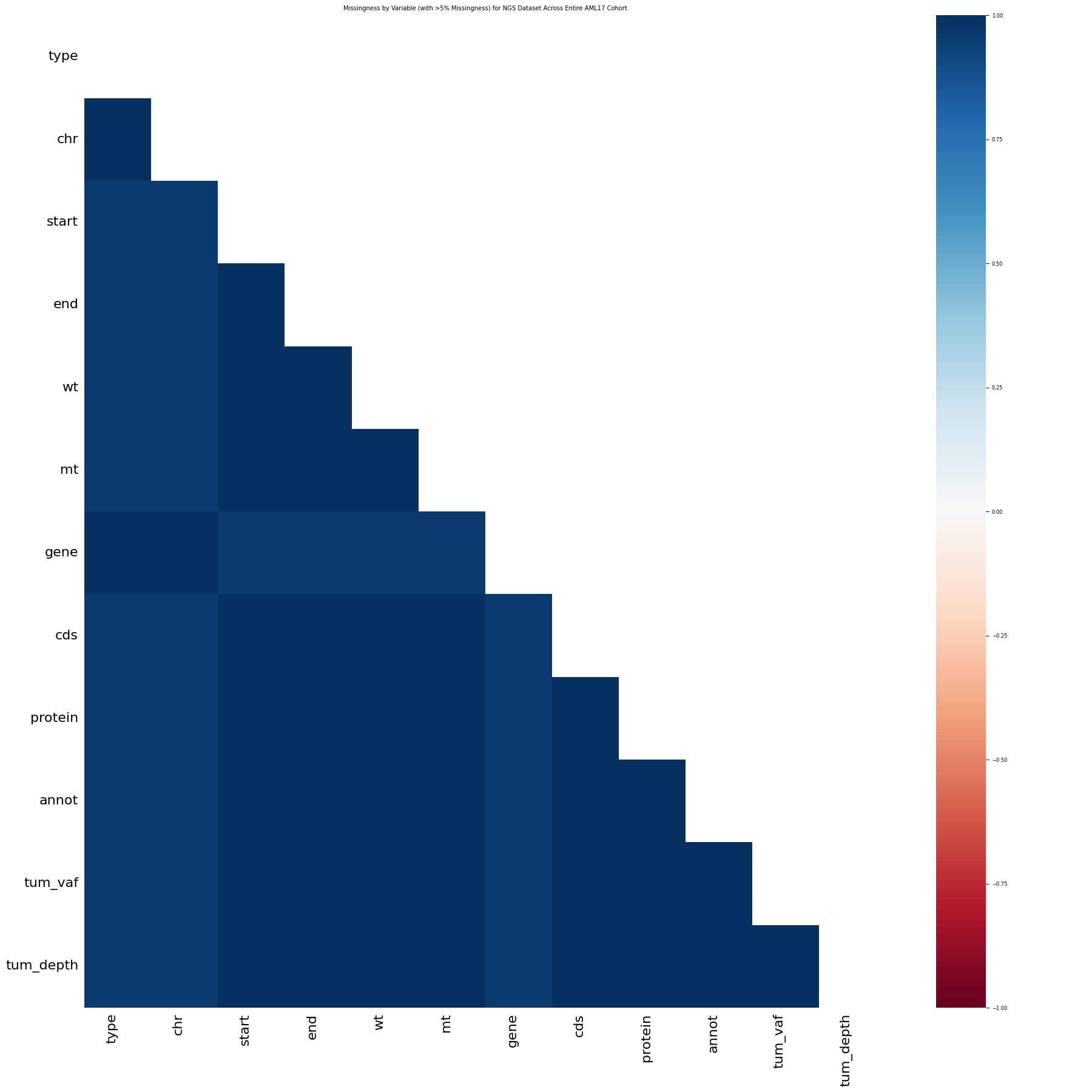

Supplement: Multimedia Appendix 10 [file bioinform-v7-e75678-s010.zip › missingness_visualisation/whole_cohort/NGS_heatmap.png]

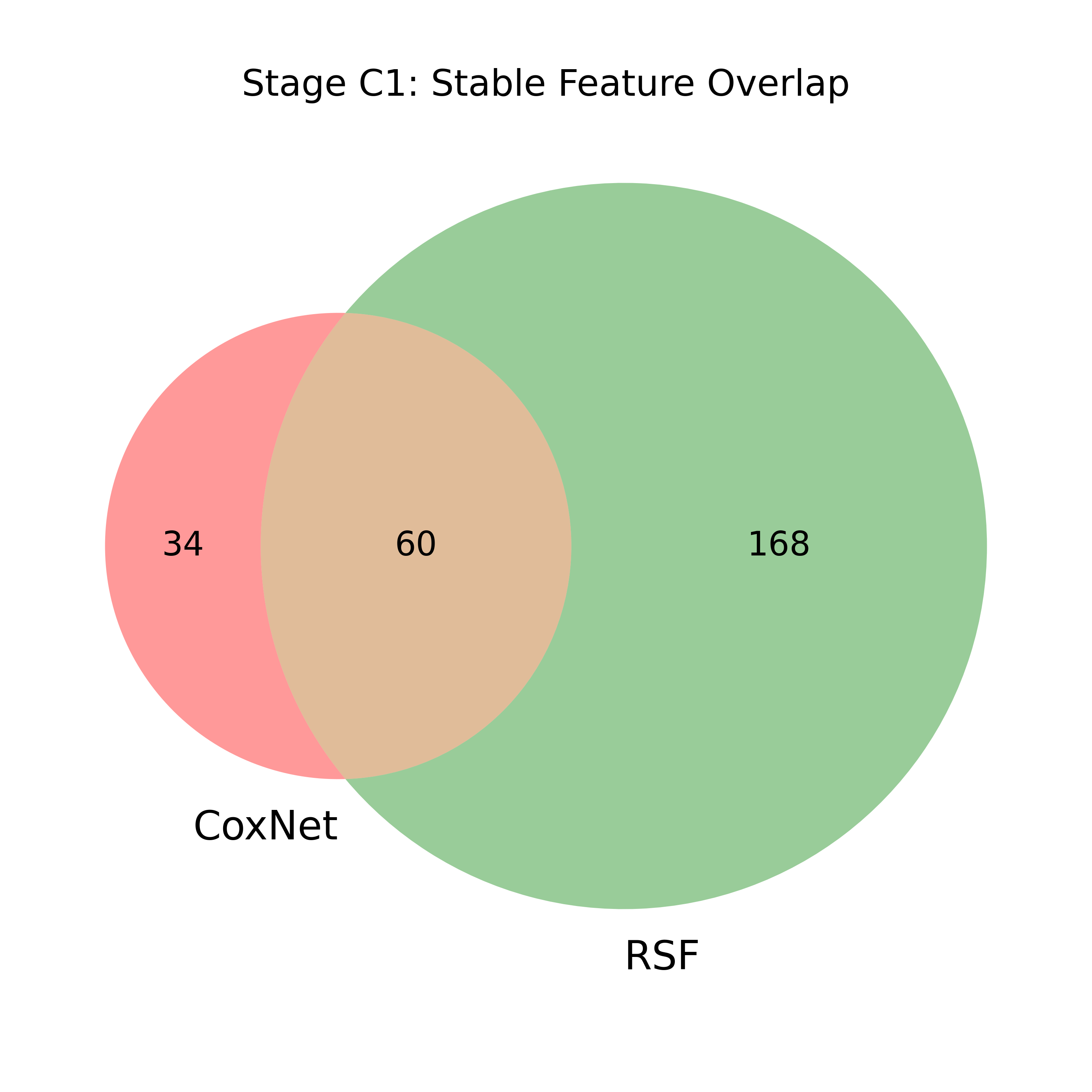

Supplement: Multimedia Appendix 11 [file bioinform-v7-e75678-s011.zip › final_stage_model_measurements/feature_importance/post_feature_reduction_measurements/stageC1_model_overlap.png]

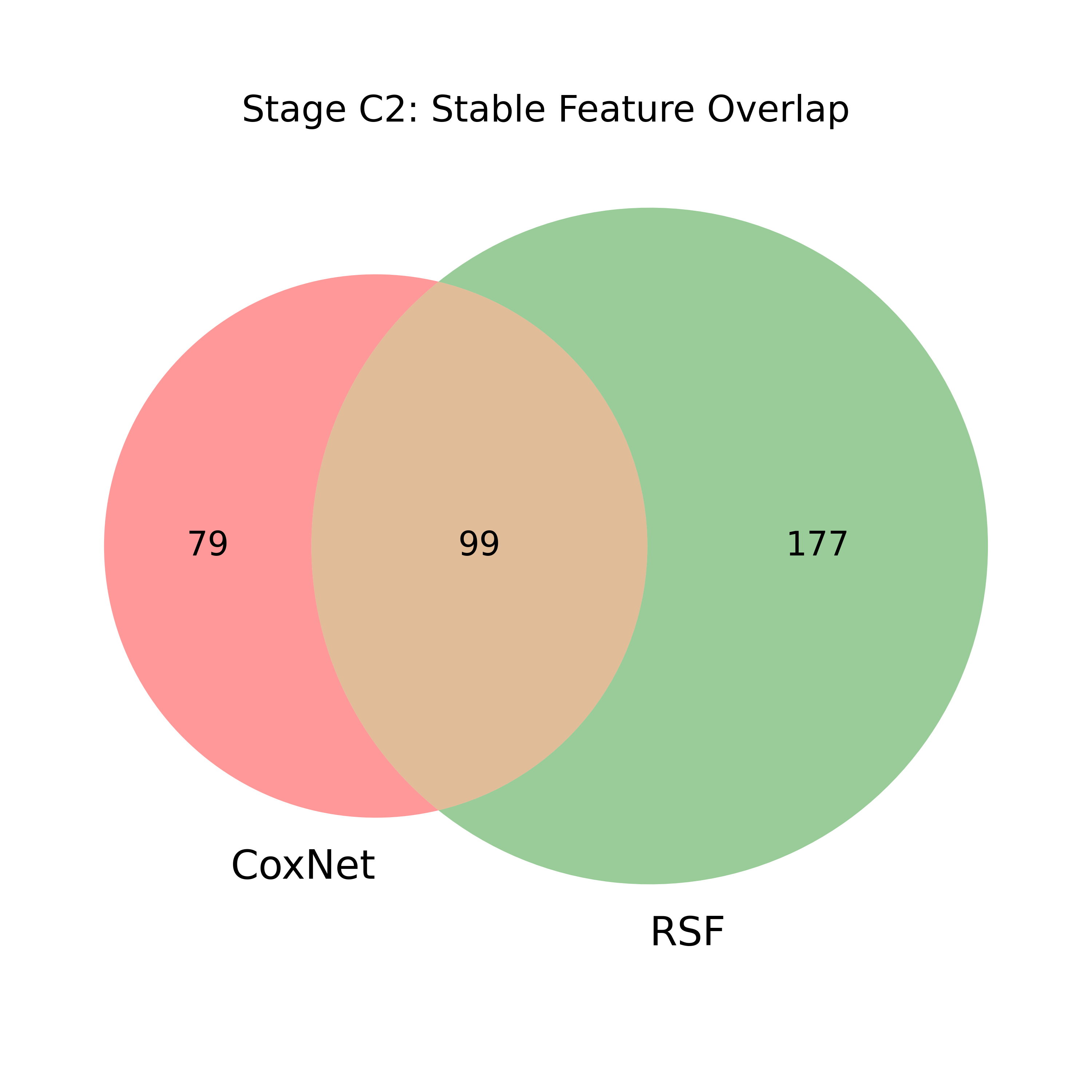

Supplement: Multimedia Appendix 11 [file bioinform-v7-e75678-s011.zip › final_stage_model_measurements/feature_importance/post_feature_reduction_measurements/stageC2_model_overlap.png]

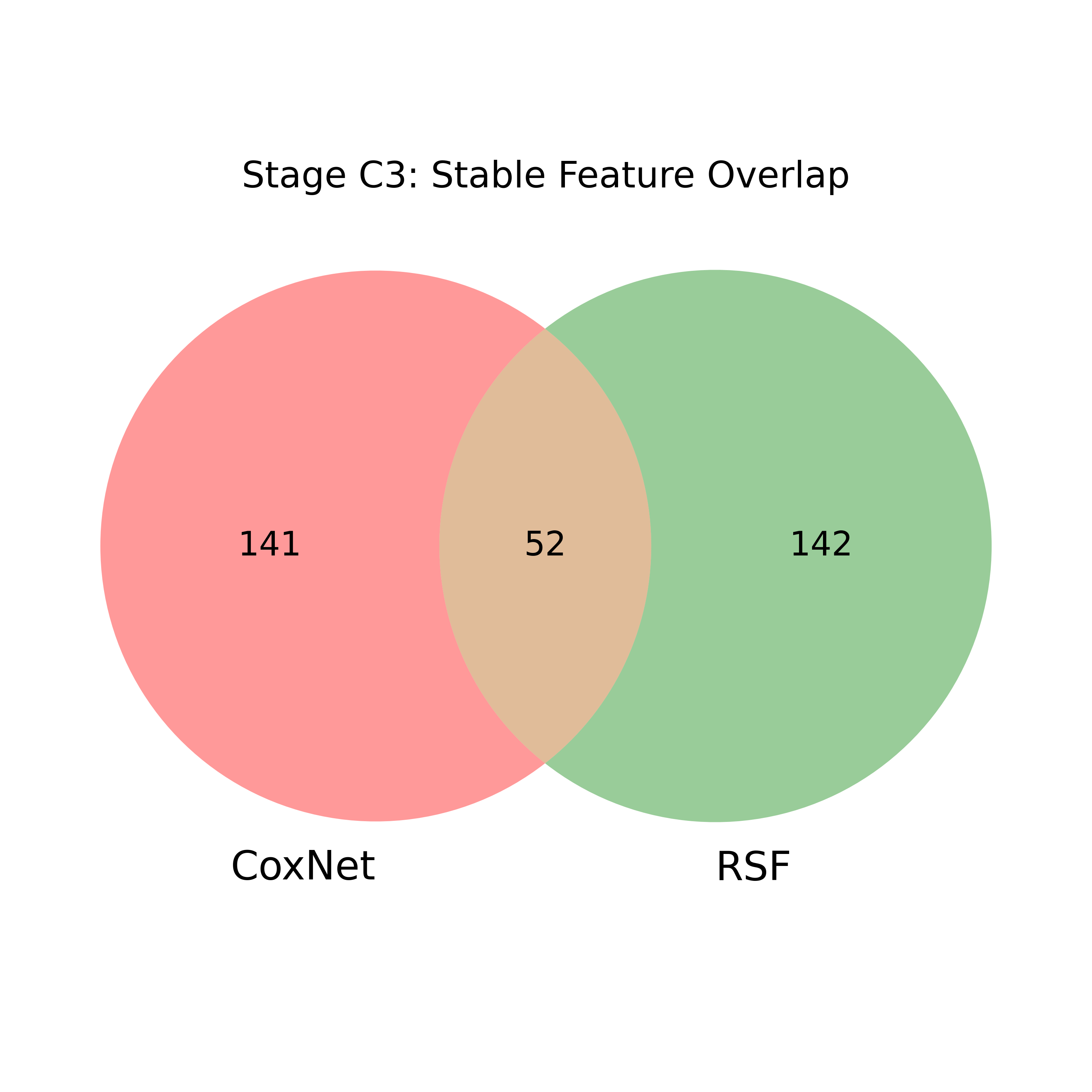

Supplement: Multimedia Appendix 11 [file bioinform-v7-e75678-s011.zip › final_stage_model_measurements/feature_importance/post_feature_reduction_measurements/stageC3_model_overlap.png]

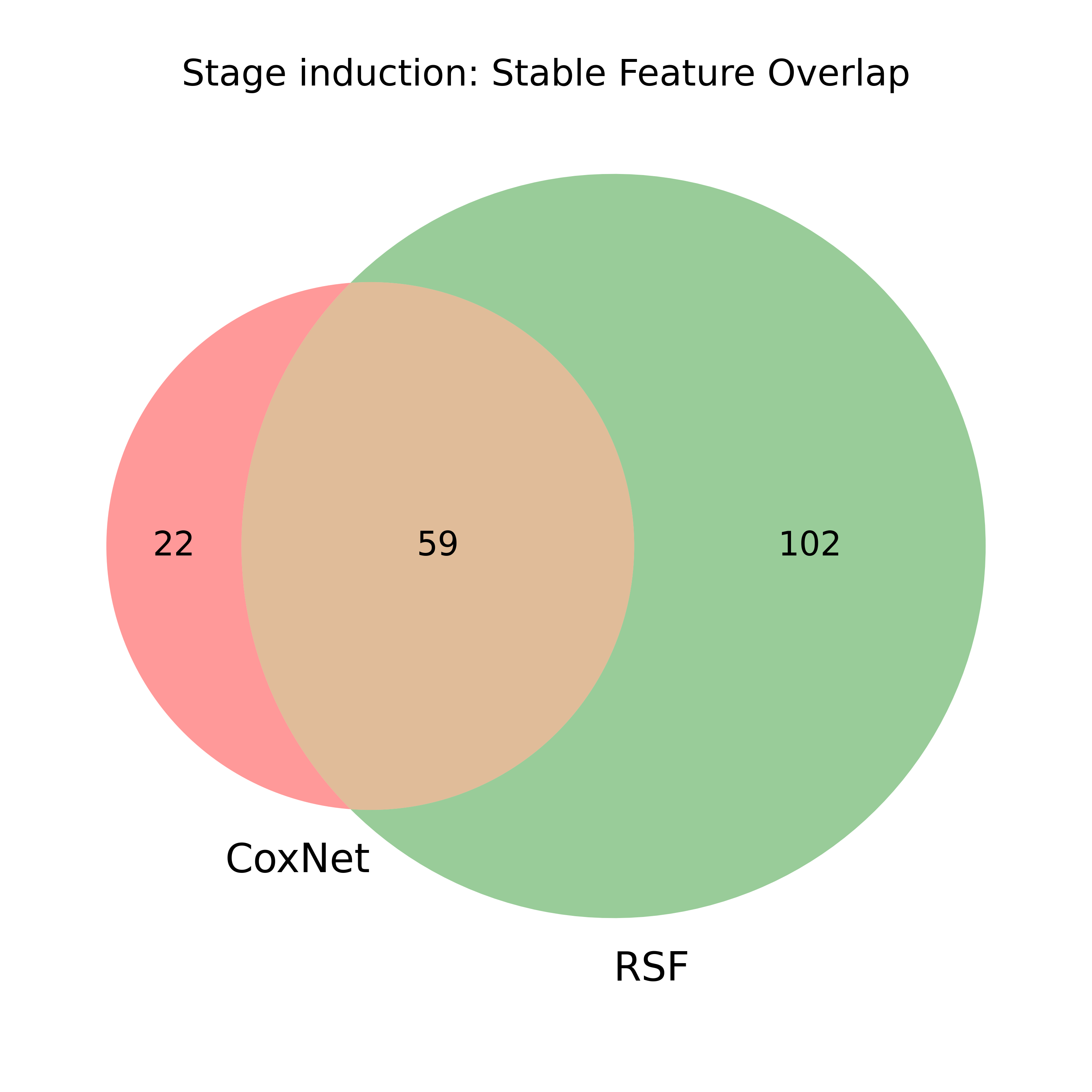

Supplement: Multimedia Appendix 11 [file bioinform-v7-e75678-s011.zip › final_stage_model_measurements/feature_importance/post_feature_reduction_measurements/stageinitial_diagnosis_model_overlap.png]

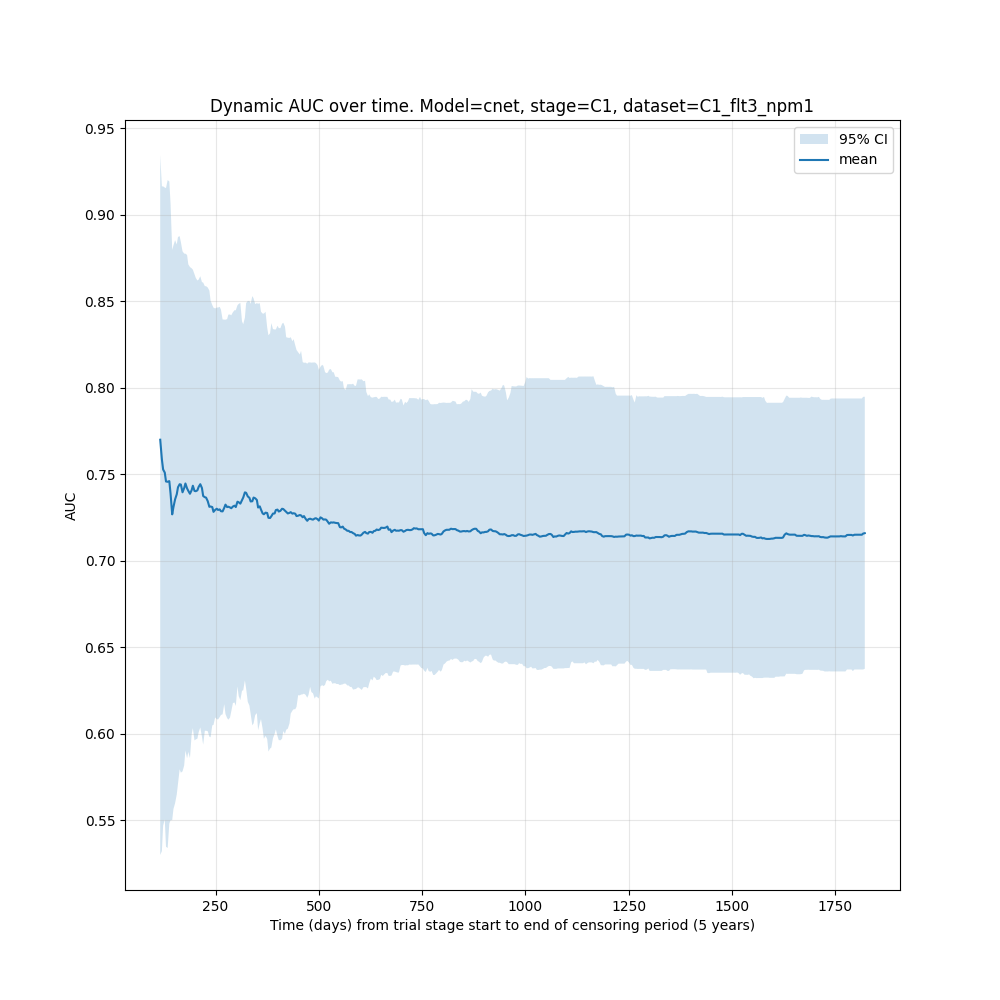

Supplement: Multimedia Appendix 11 [file bioinform-v7-e75678-s011.zip › final_stage_model_measurements/performance_metrics/cnet/dynamic_auc_plots/individual_stages/cnet__post_C1__C1_flt3_npm1__dynamic_auc.png]

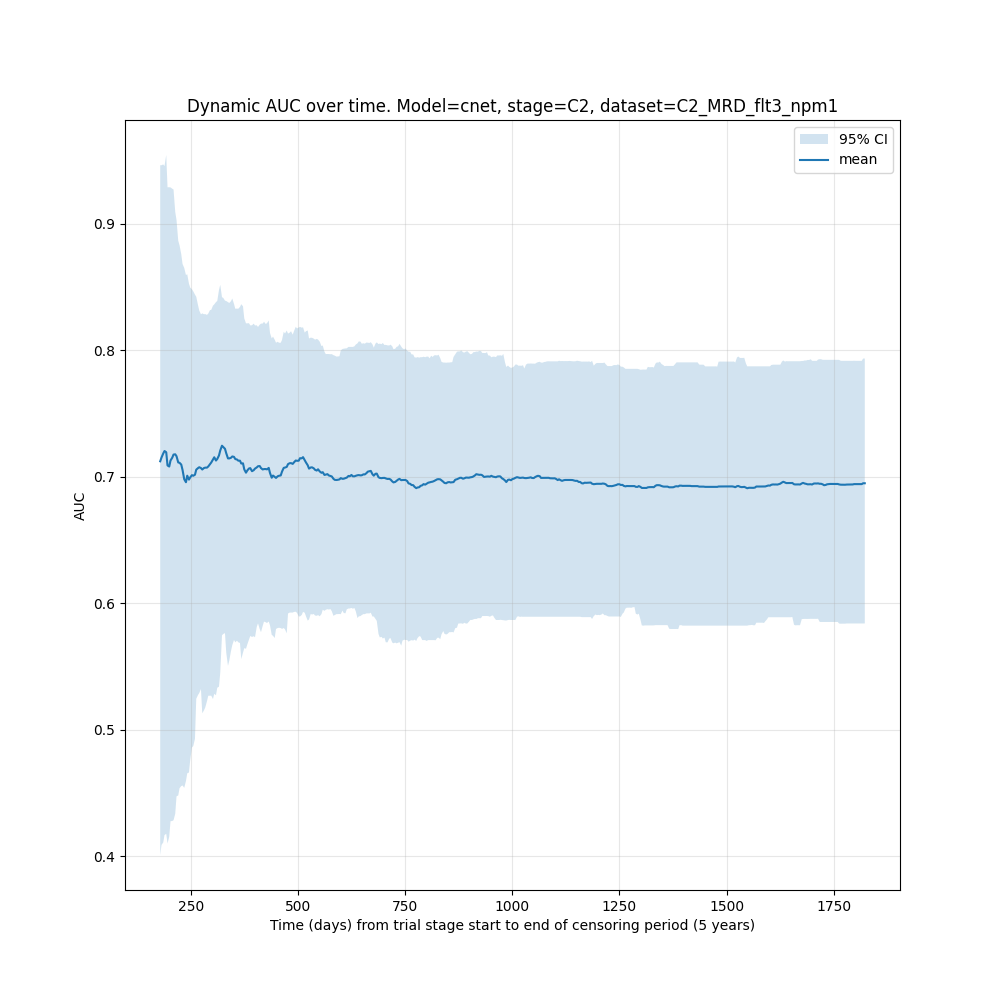

Supplement: Multimedia Appendix 11 [file bioinform-v7-e75678-s011.zip › final_stage_model_measurements/performance_metrics/cnet/dynamic_auc_plots/individual_stages/cnet__post_C2__C2_MRD_flt3_npm1__dynamic_auc.png]

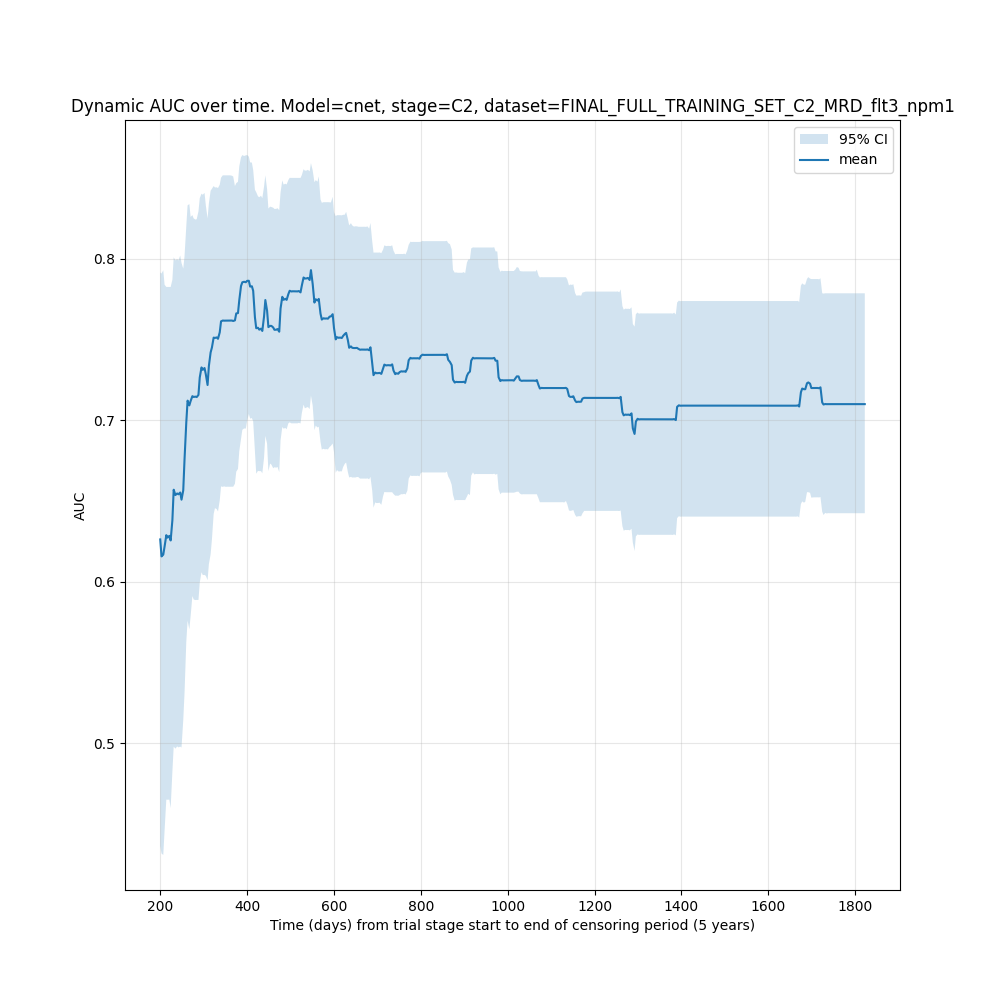

Supplement: Multimedia Appendix 11 [file bioinform-v7-e75678-s011.zip › final_stage_model_measurements/performance_metrics/cnet/dynamic_auc_plots/individual_stages/cnet__post_C2__FINAL_FULL_TRAINING_SET_C2_MRD_flt3_npm1__dynamic_auc.png]

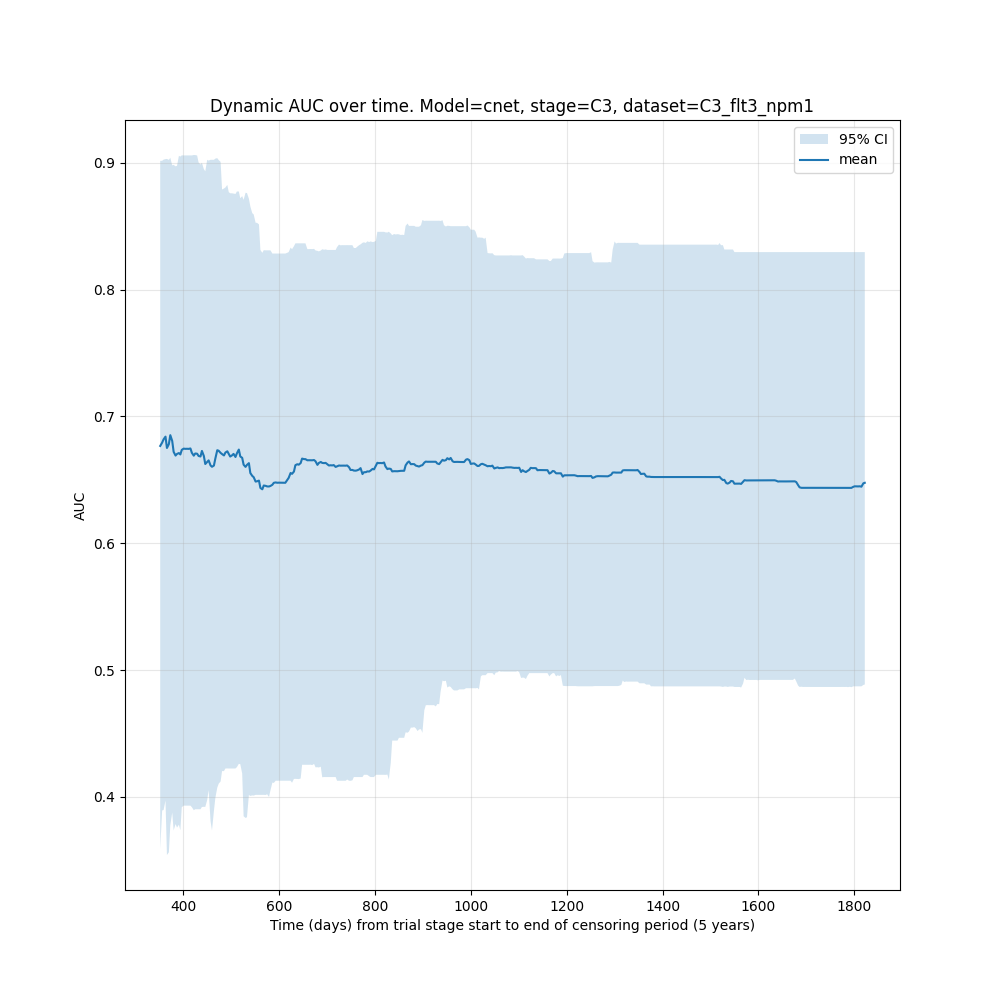

Supplement: Multimedia Appendix 11 [file bioinform-v7-e75678-s011.zip › final_stage_model_measurements/performance_metrics/cnet/dynamic_auc_plots/individual_stages/cnet__post_C3__C3_flt3_npm1__dynamic_auc.png]

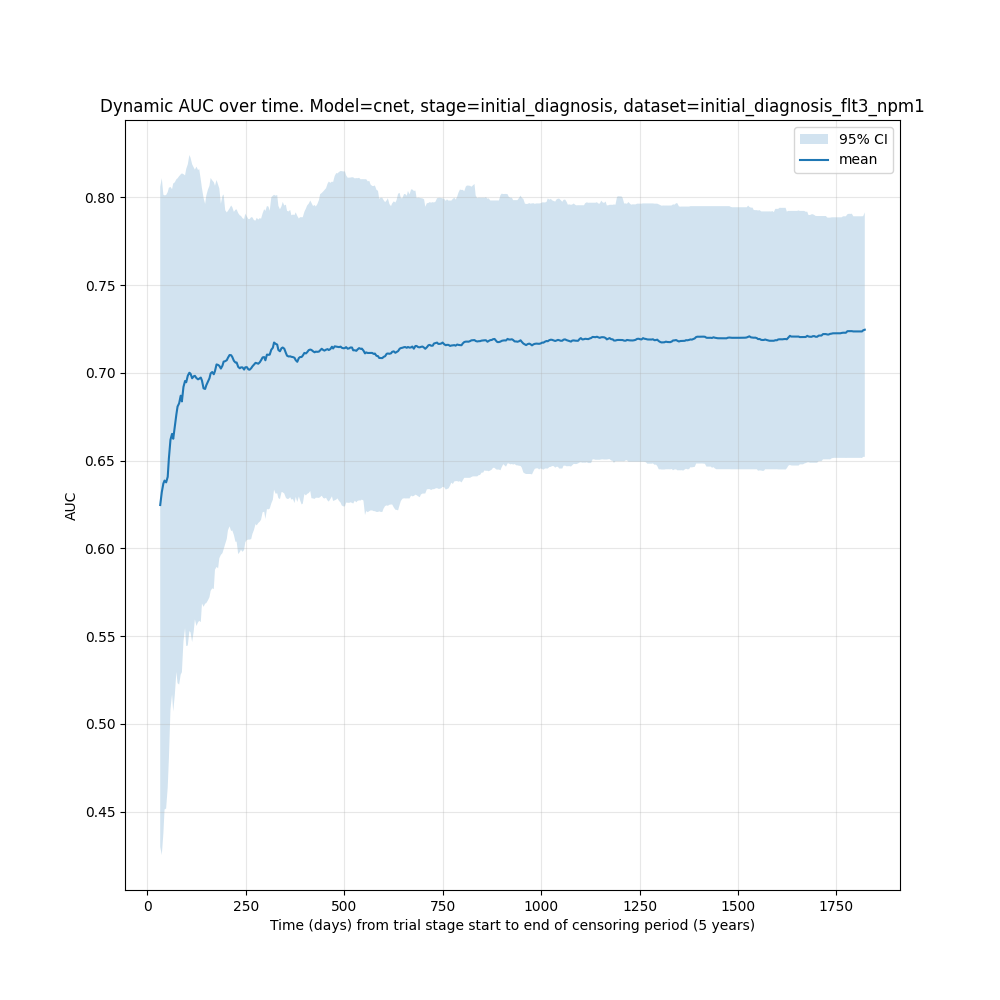

Supplement: Multimedia Appendix 11 [file bioinform-v7-e75678-s011.zip › final_stage_model_measurements/performance_metrics/cnet/dynamic_auc_plots/individual_stages/cnet__post_initial_diagnosis__initial_diagnosis_flt3_npm1__dynamic_auc.png]

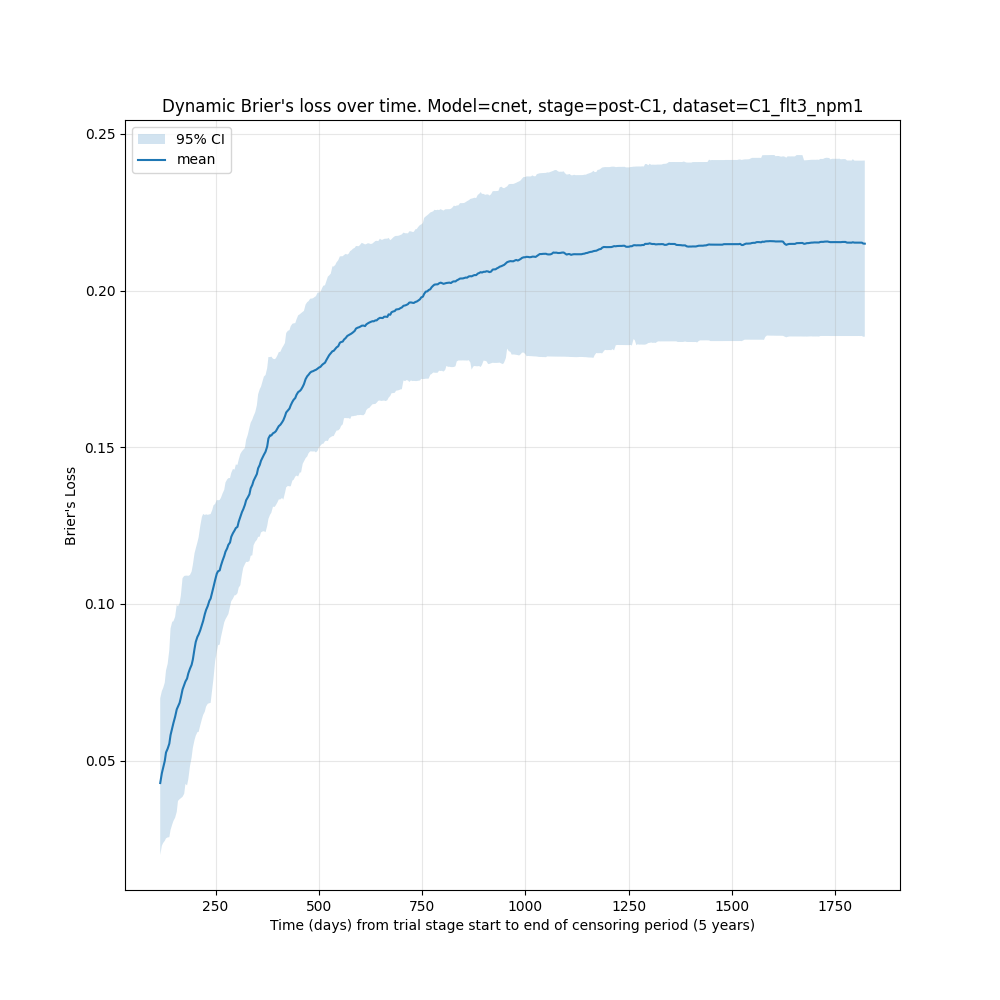

Supplement: Multimedia Appendix 11 [file bioinform-v7-e75678-s011.zip › final_stage_model_measurements/performance_metrics/cnet/dynamic_briers_plots/individual_stages/cnet__post_C1__C1_flt3_npm1__dynamic_briers.png]

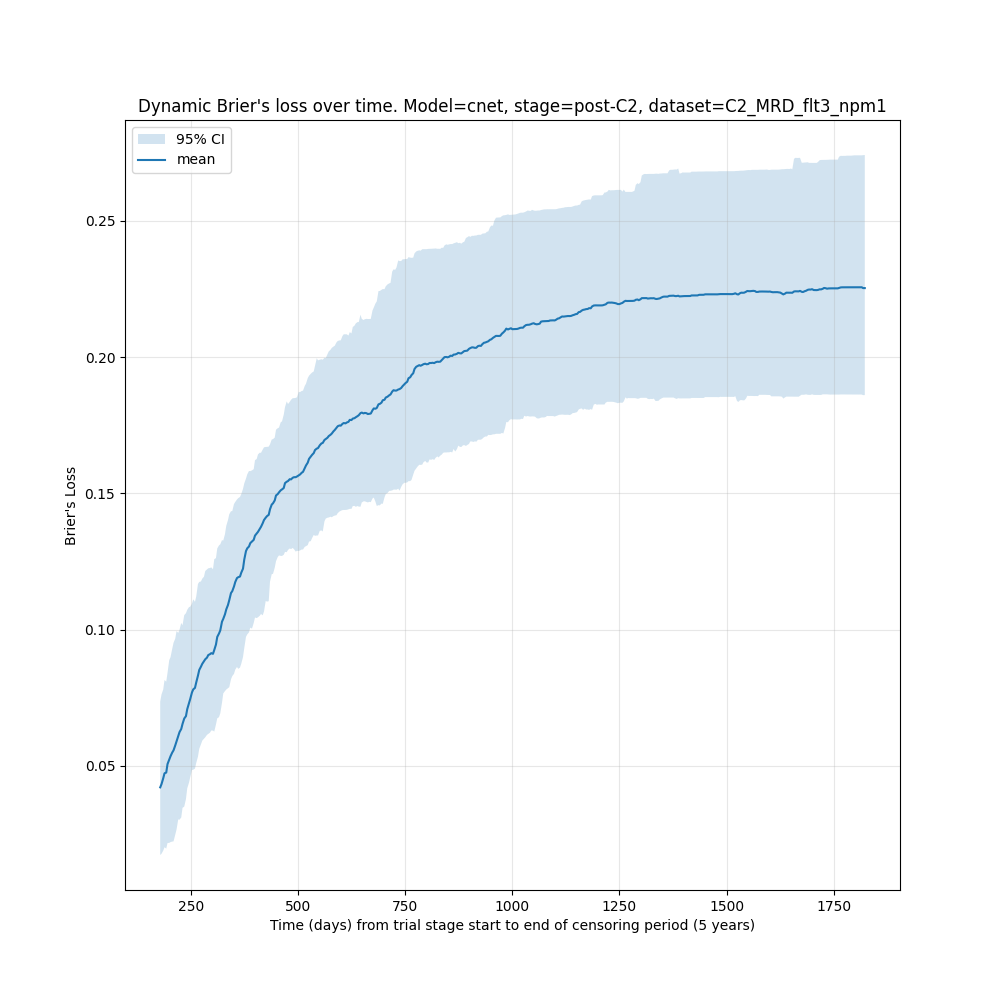

Supplement: Multimedia Appendix 11 [file bioinform-v7-e75678-s011.zip › final_stage_model_measurements/performance_metrics/cnet/dynamic_briers_plots/individual_stages/cnet__post_C2__C2_MRD_flt3_npm1__dynamic_briers.png]

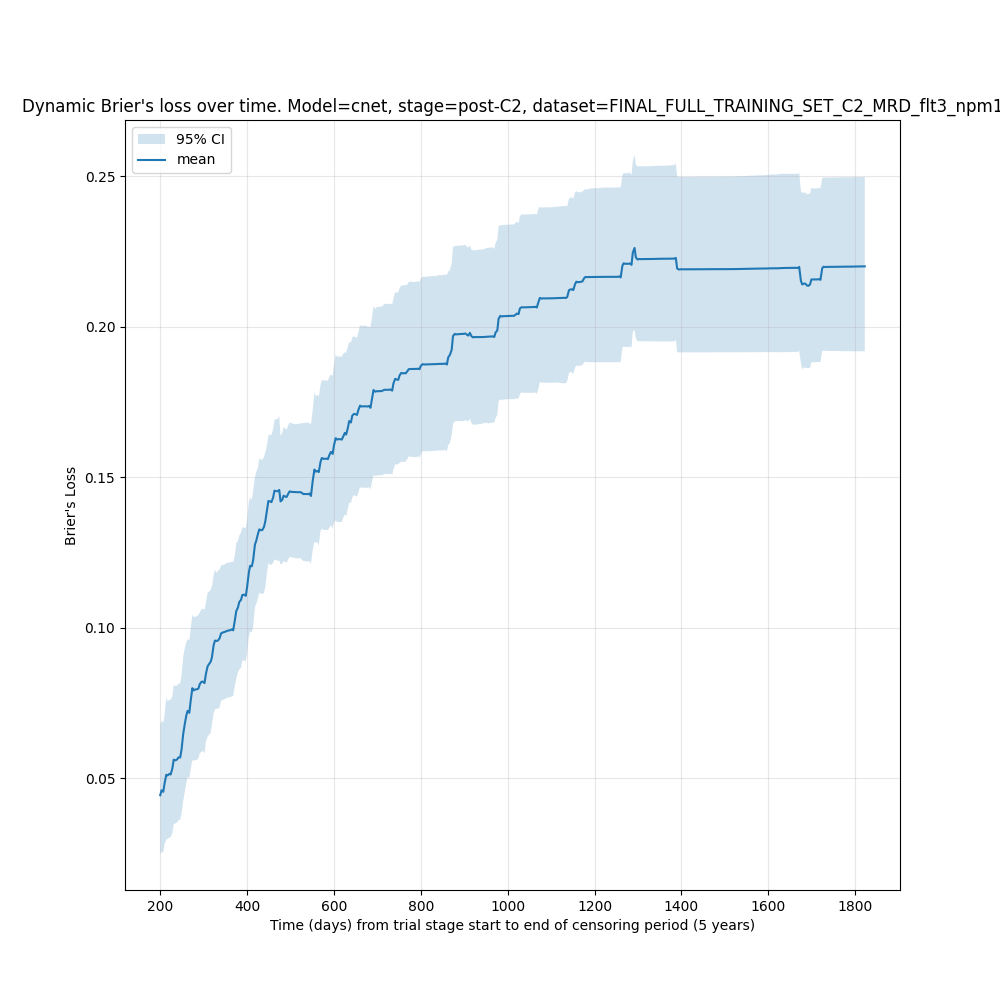

Supplement: Multimedia Appendix 11 [file bioinform-v7-e75678-s011.zip › final_stage_model_measurements/performance_metrics/cnet/dynamic_briers_plots/individual_stages/cnet__post_C2__FINAL_FULL_TRAINING_SET_C2_MRD_flt3_npm1__dynamic_briers.png]

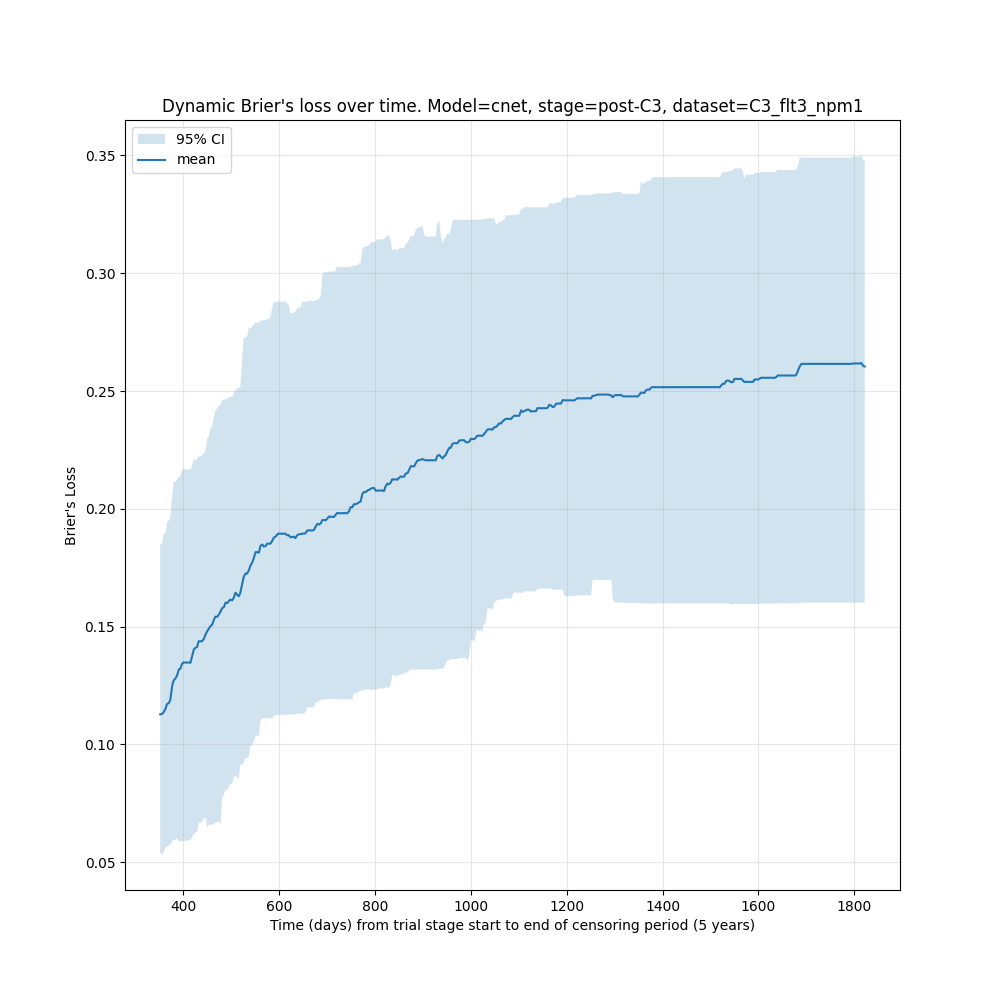

Supplement: Multimedia Appendix 11 [file bioinform-v7-e75678-s011.zip › final_stage_model_measurements/performance_metrics/cnet/dynamic_briers_plots/individual_stages/cnet__post_C3__C3_flt3_npm1__dynamic_briers.png]

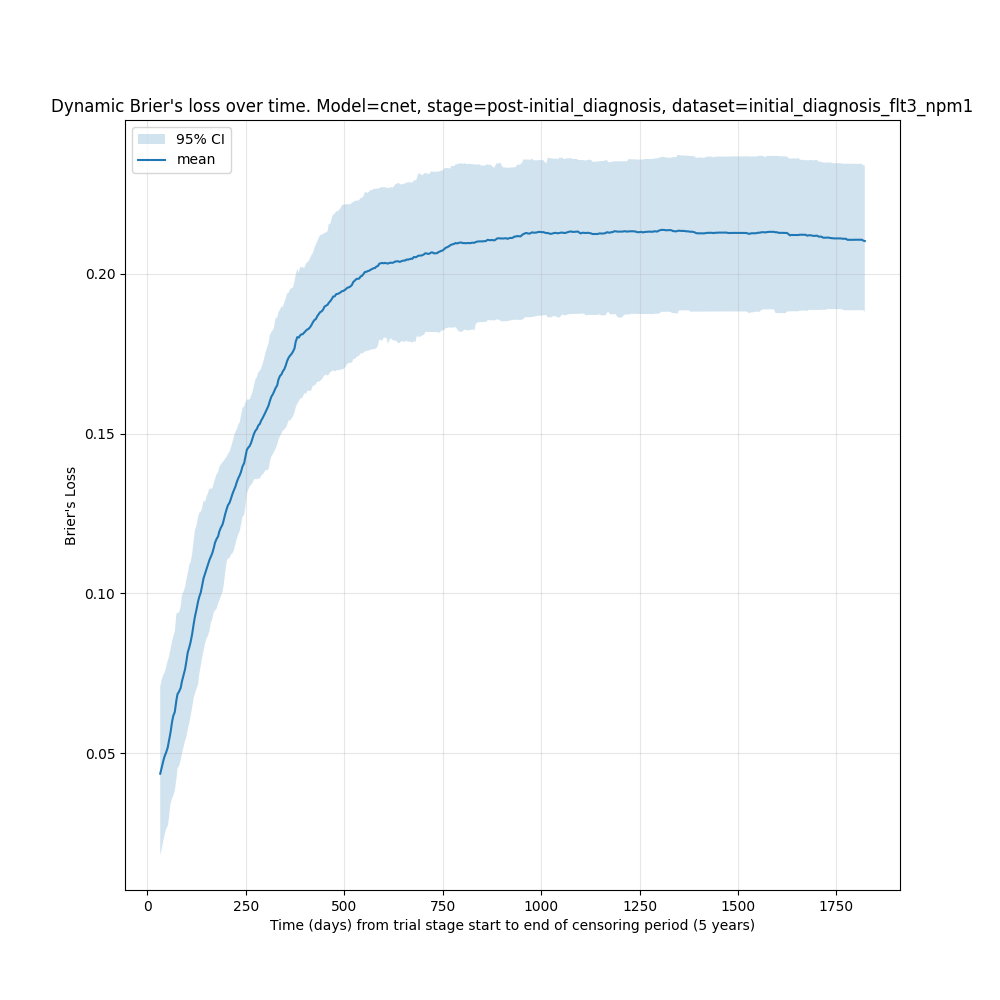

Supplement: Multimedia Appendix 11 [file bioinform-v7-e75678-s011.zip › final_stage_model_measurements/performance_metrics/cnet/dynamic_briers_plots/individual_stages/cnet__post_initial_diagnosis__initial_diagnosis_flt3_npm1__dynamic_briers.png]

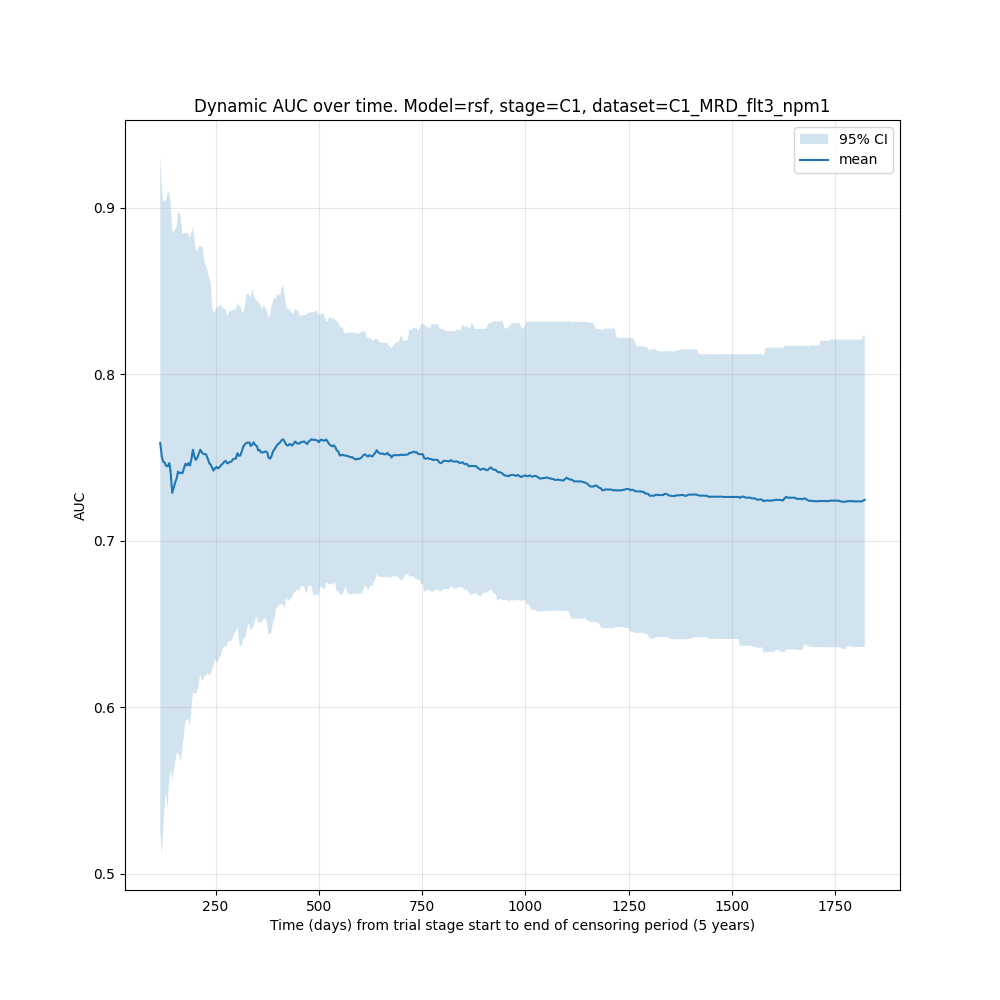

Supplement: Multimedia Appendix 11 [file bioinform-v7-e75678-s011.zip › final_stage_model_measurements/performance_metrics/rsf/dynamic_auc_plots/rsf__post_C1__C1_MRD_flt3_npm1__dynamic_auc.png]

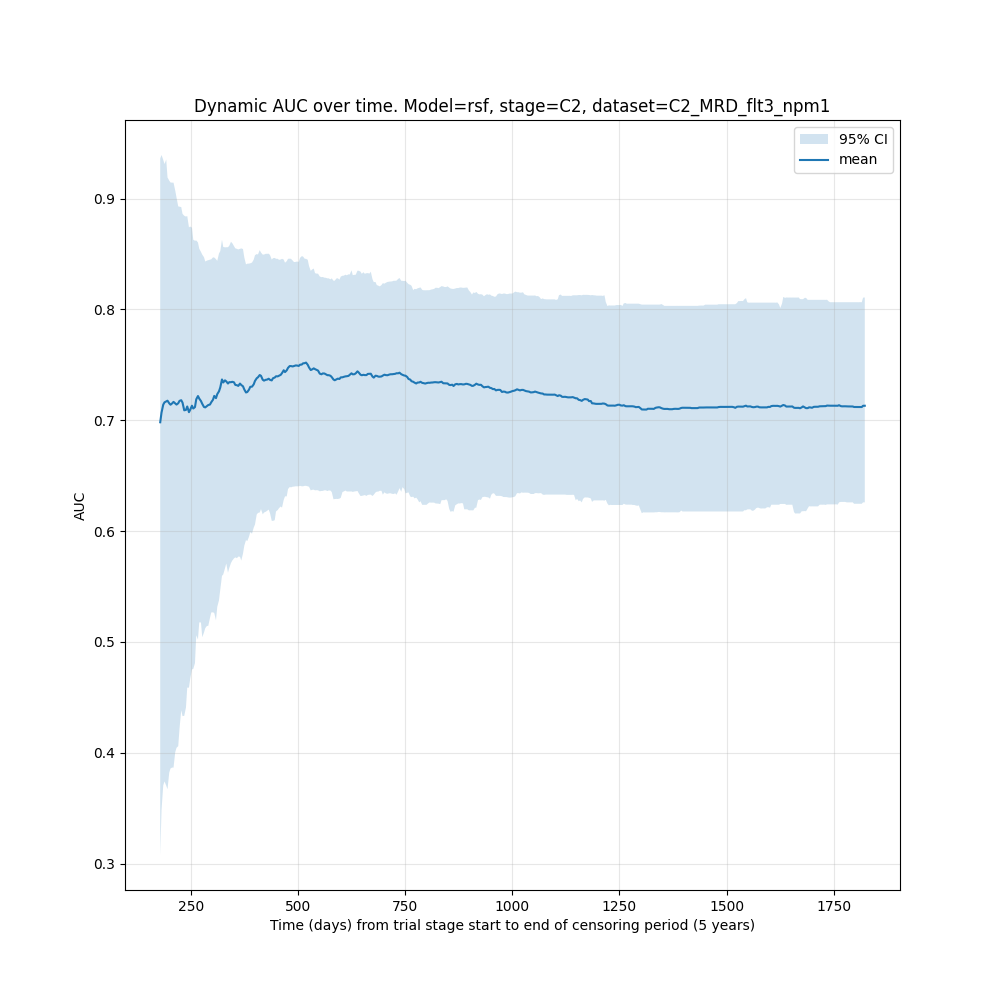

Supplement: Multimedia Appendix 11 [file bioinform-v7-e75678-s011.zip › final_stage_model_measurements/performance_metrics/rsf/dynamic_auc_plots/rsf__post_C2__C2_MRD_flt3_npm1__dynamic_auc.png]

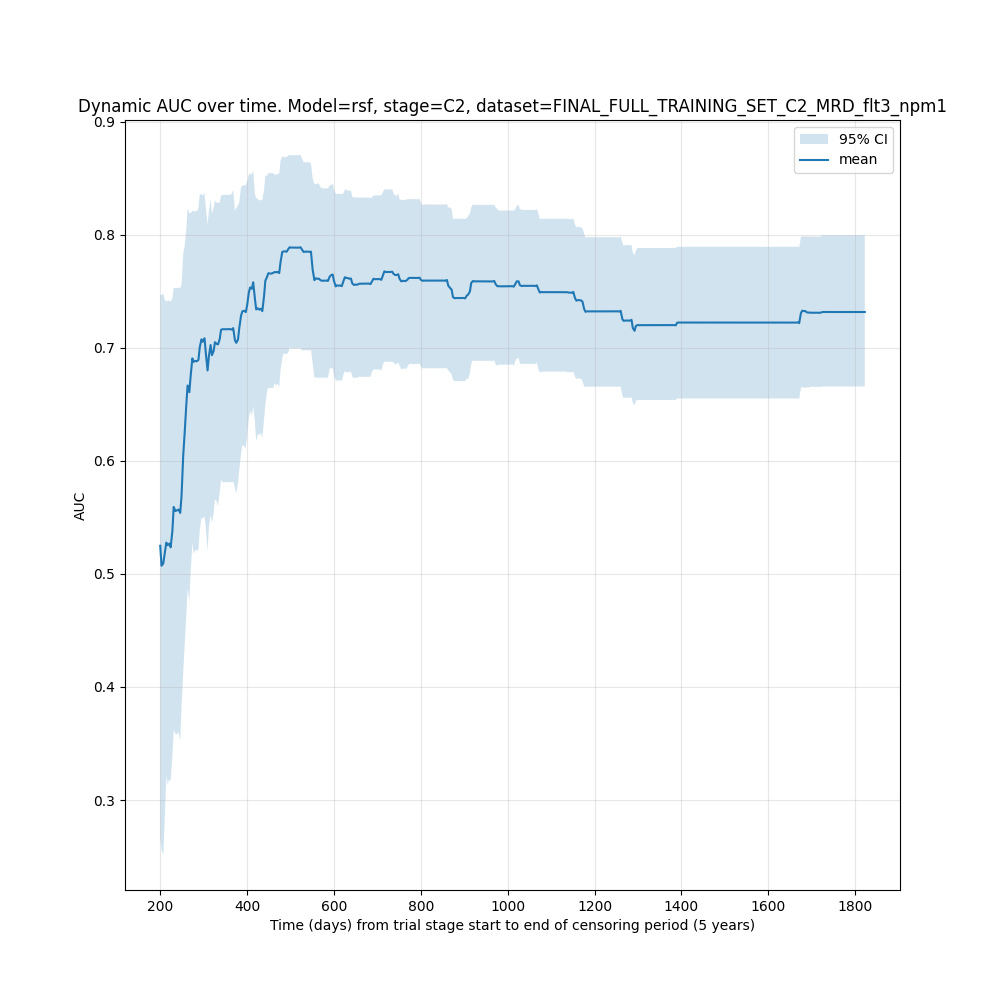

Supplement: Multimedia Appendix 11 [file bioinform-v7-e75678-s011.zip › final_stage_model_measurements/performance_metrics/rsf/dynamic_auc_plots/rsf__post_C2__FINAL_FULL_TRAINING_SET_C2_MRD_flt3_npm1__dynamic_auc.png]

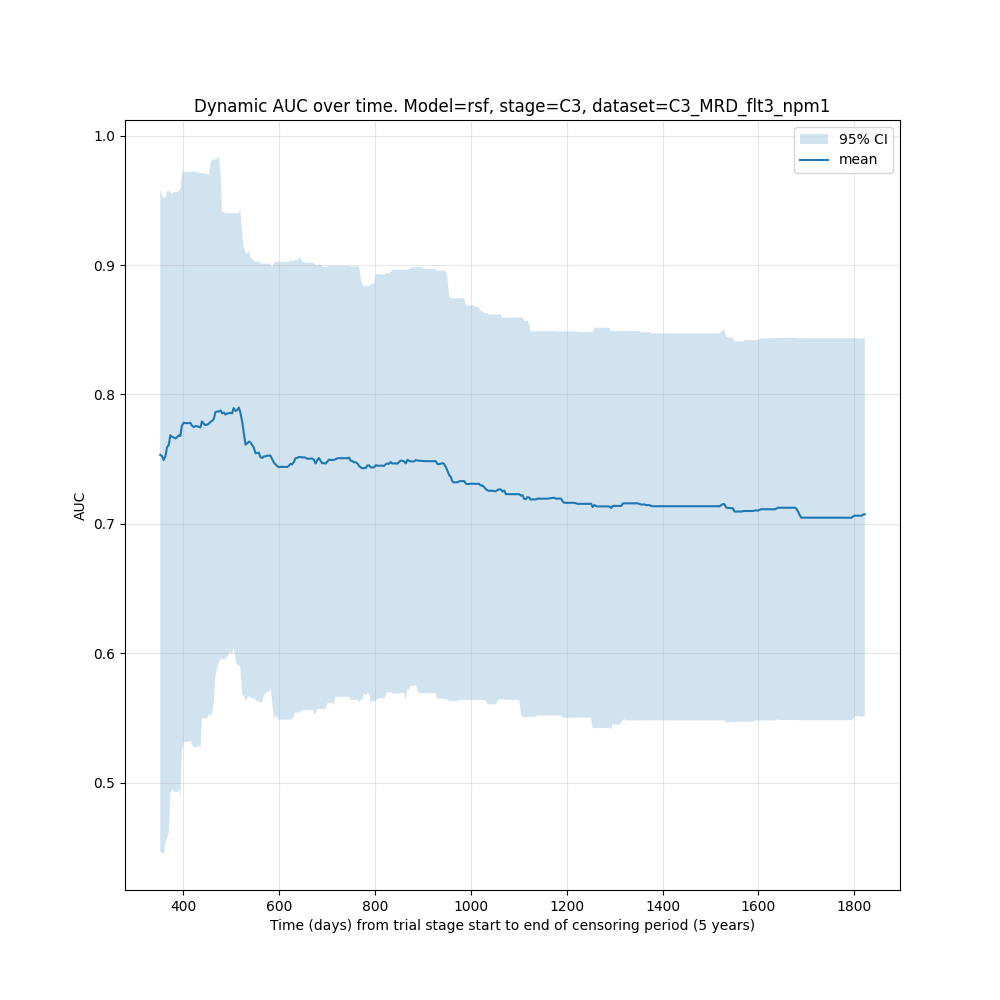

Supplement: Multimedia Appendix 11 [file bioinform-v7-e75678-s011.zip › final_stage_model_measurements/performance_metrics/rsf/dynamic_auc_plots/rsf__post_C3__C3_MRD_flt3_npm1__dynamic_auc.png]

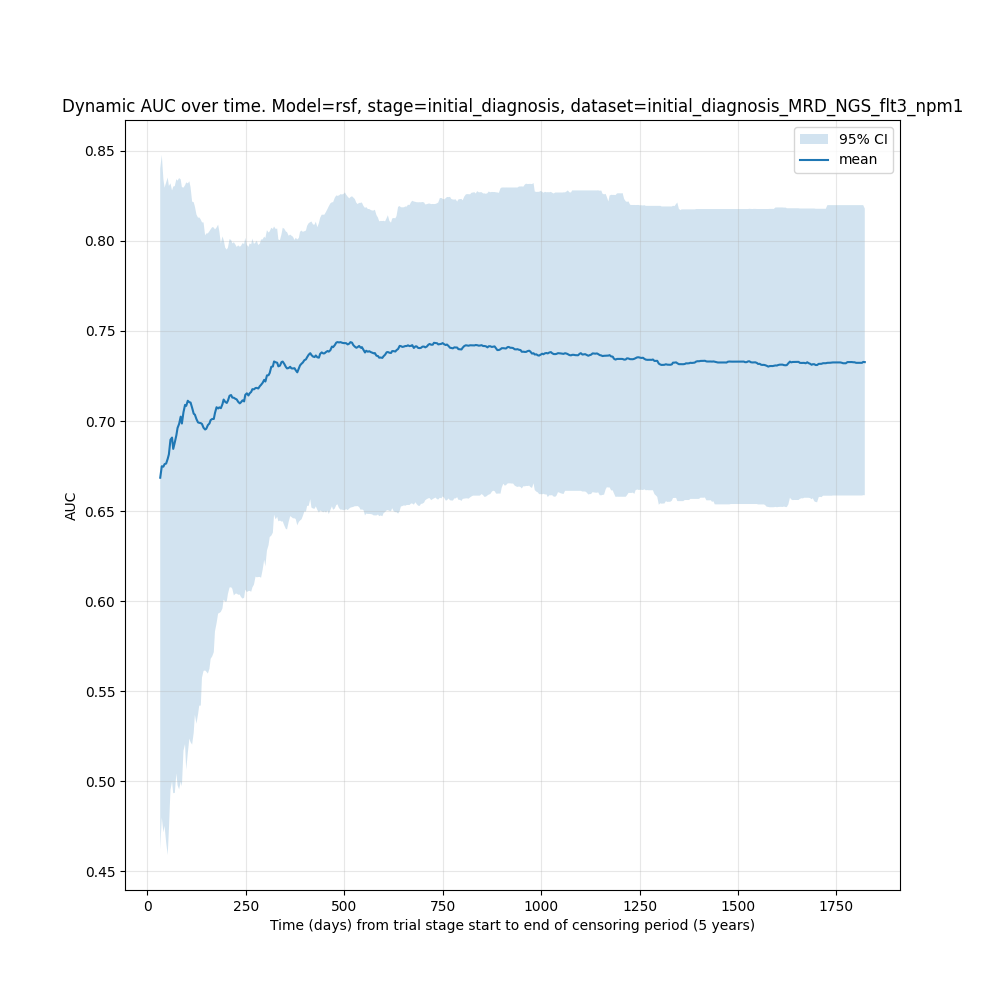

Supplement: Multimedia Appendix 11 [file bioinform-v7-e75678-s011.zip › final_stage_model_measurements/performance_metrics/rsf/dynamic_auc_plots/rsf__post_initial_diagnosis__initial_diagnosis_MRD_NGS_flt3_npm1__dynamic_auc.png]

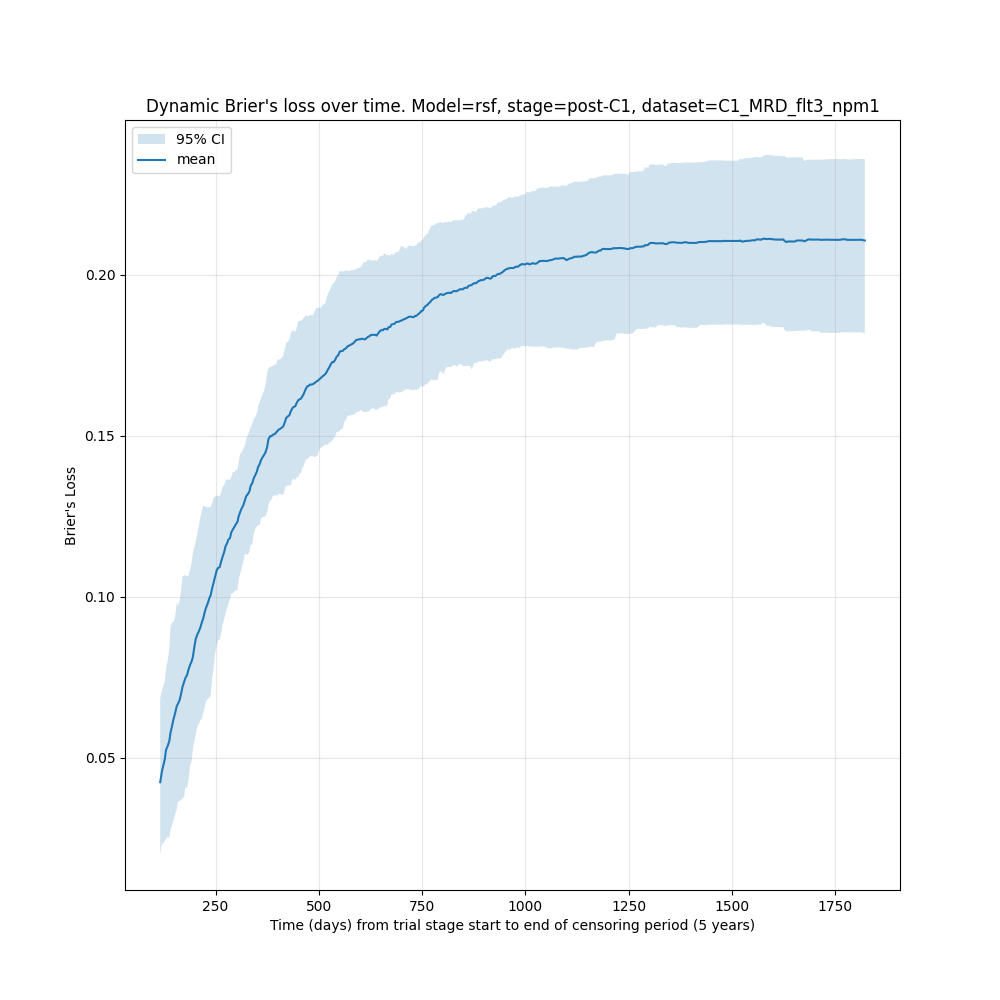

Supplement: Multimedia Appendix 11 [file bioinform-v7-e75678-s011.zip › final_stage_model_measurements/performance_metrics/rsf/dynamic_briers_plots/rsf__post_C1__C1_MRD_flt3_npm1__dynamic_briers.png]

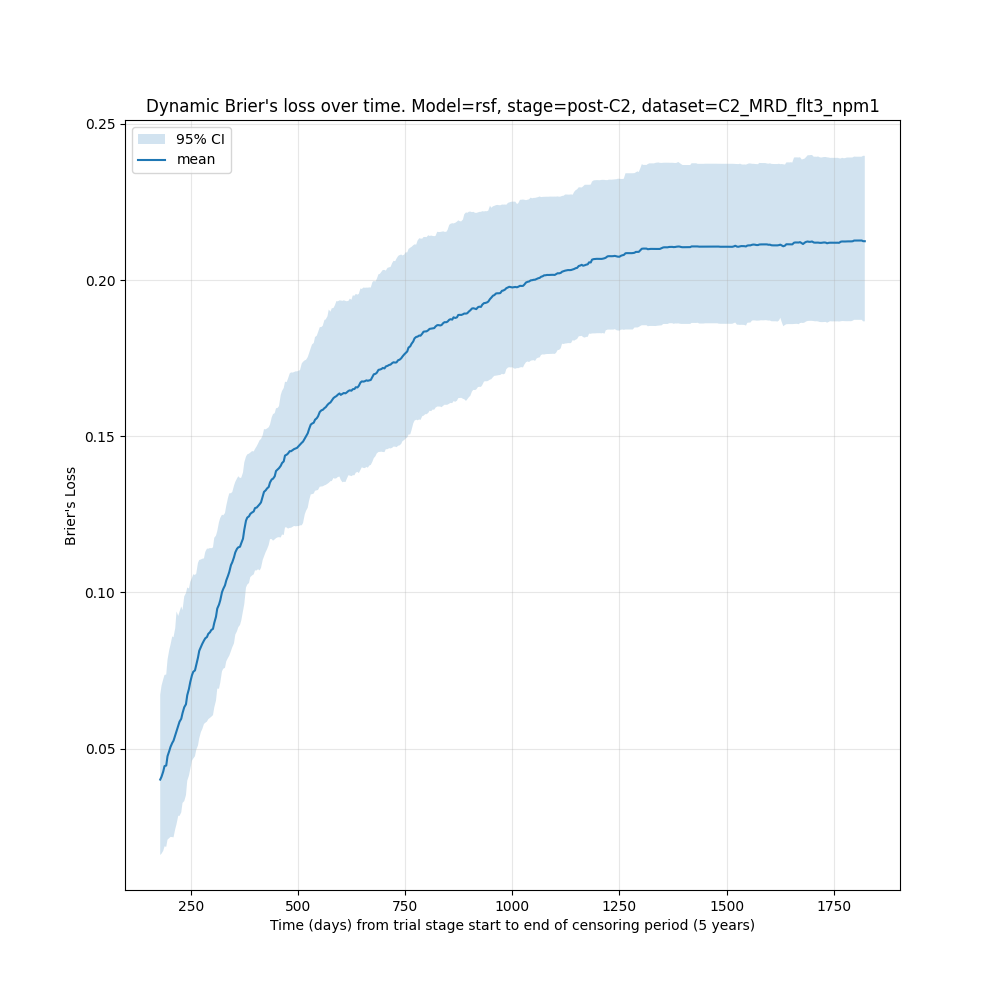

Supplement: Multimedia Appendix 11 [file bioinform-v7-e75678-s011.zip › final_stage_model_measurements/performance_metrics/rsf/dynamic_briers_plots/rsf__post_C2__C2_MRD_flt3_npm1__dynamic_briers.png]

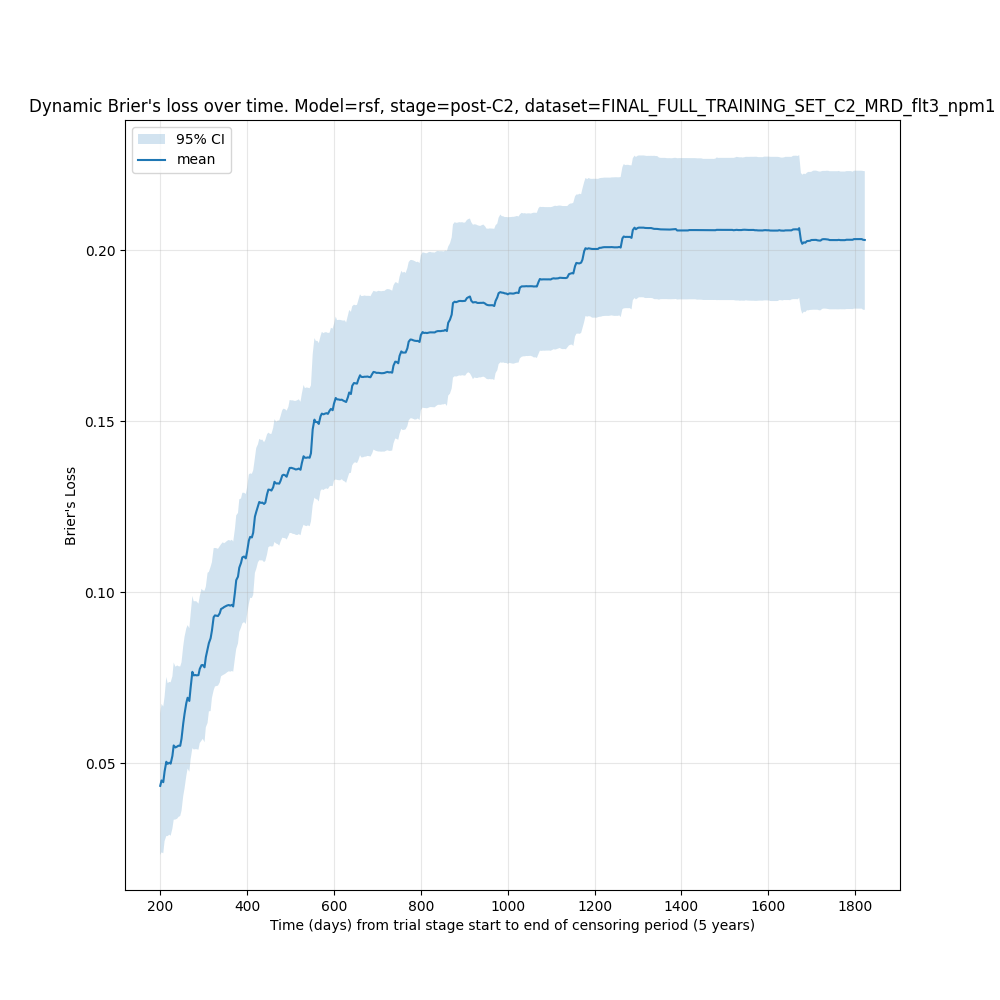

Supplement: Multimedia Appendix 11 [file bioinform-v7-e75678-s011.zip › final_stage_model_measurements/performance_metrics/rsf/dynamic_briers_plots/rsf__post_C2__FINAL_FULL_TRAINING_SET_C2_MRD_flt3_npm1__dynamic_briers.png]

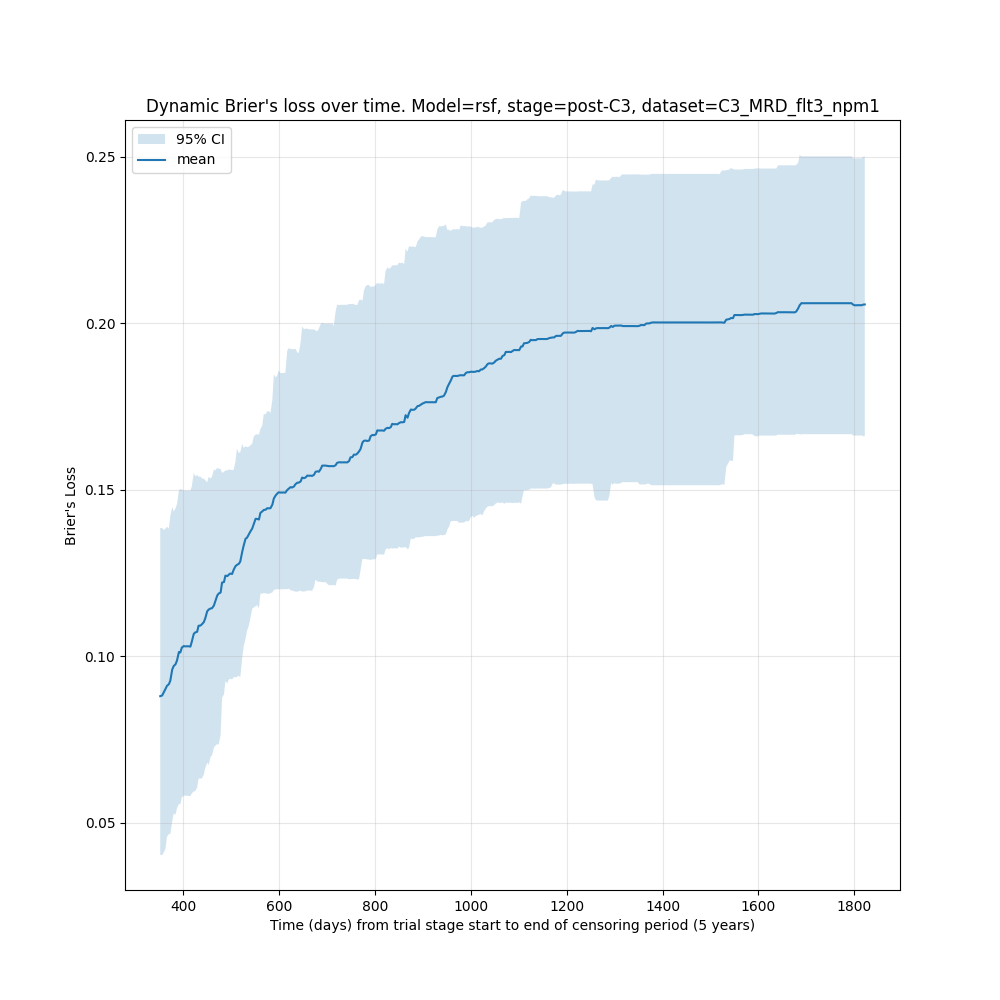

Supplement: Multimedia Appendix 11 [file bioinform-v7-e75678-s011.zip › final_stage_model_measurements/performance_metrics/rsf/dynamic_briers_plots/rsf__post_C3__C3_MRD_flt3_npm1__dynamic_briers.png]

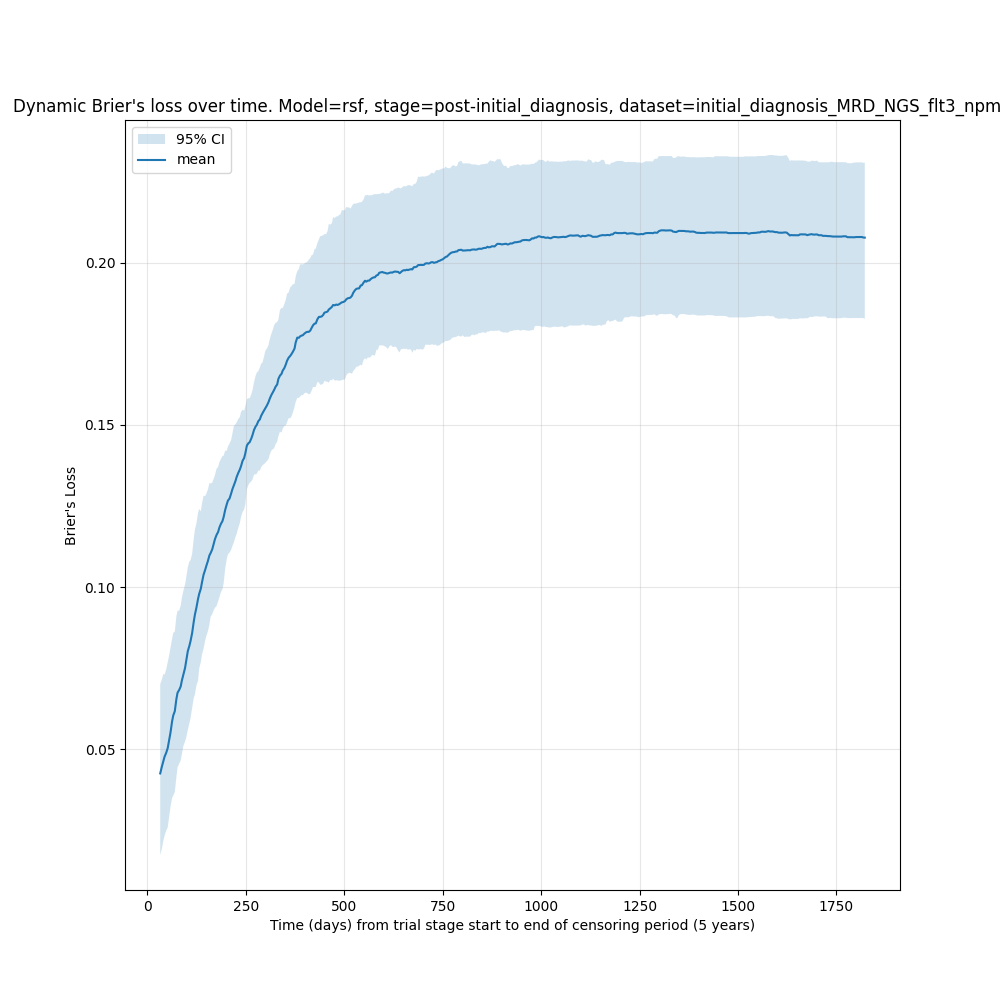

Supplement: Multimedia Appendix 11 [file bioinform-v7-e75678-s011.zip › final_stage_model_measurements/performance_metrics/rsf/dynamic_briers_plots/rsf__post_initial_diagnosis__initial_diagnosis_MRD_NGS_flt3_npm1__dynamic_briers.png]
